# Supplementary material for: Orthogonal Factorial Designs for Trials of Therapist‐Delivered Interventions: Randomizing Intervention‐Therapist Combinations to Patients
Source: Stat Med. 2026 Nov 10;45(25-27):e70813. doi: 10.1002/sim.70813 (PMC13749633; doi:10.1002/sim.70813)
Supplement: Supplementary file 1 [file SIM-45-0-s001.docx]

**Supplementary File H – Additional Simulation Results**

Columns:

$\hat{\delta}_{1}:$	the estimated fixed intervention effect,

SE($\hat{\delta}_{1}$):	the estimated standard error of $\hat{\delta}_{1}$,

$\hat{\sigma}_{u1}^{2}:$	the estimated between-therapist variance,

$\hat{\sigma}_{v1}^{2}:$	the estimated between-therapist variance in the intervention effect,

$\delta_{2}$:	the fixed coefficient of the baseline patient covariate associated with how therapists are allocated to patients, and,

$\delta_{3}:$	the fixed coefficient of baseline patient covariate associated with how therapists are allocated to patients with knowledge of the randomised intervention.

Note: The true values of the parameters $\delta_{1},$ $\sigma_{u1}^{2}$ and $\sigma_{v1}^{2}$ are given in Table 2 in the main text of the paper.

**Table H1. Simulation results for the randomised block factorial design.**

| $\boldsymbol{\delta}_{\boldsymbol{3}}$ | $\boldsymbol{\delta}_{\boldsymbol{2}}$ | ${\hat{\boldsymbol{\delta}}}_{\boldsymbol{1}}$ | | **SE**$\boldsymbol{(}{\hat{\boldsymbol{\delta}}}_{\boldsymbol{1}}\boldsymbol{)}$ | | ${\hat{\boldsymbol{\sigma}}}_{\boldsymbol{u}\boldsymbol{1}}^{\boldsymbol{2}}$ | | ${\hat{\boldsymbol{\sigma}}}_{\boldsymbol{v}\boldsymbol{1}}^{\boldsymbol{2}}$ | | **Type I Error** | | **Type II Error** | | **Boundary Estimates** | |
| --- | --- | --- | --- | --- | --- | --- | --- | --- | --- | --- | --- | --- | --- | --- | --- |
| 1) Comparison of assigning therapists to patients *after* ${(D}_{12})$and *before* ${(D}_{22})$ randomising interventions to patients | | | | | | | | | | | | | | | |
|  | | $\boldsymbol{D}_{\boldsymbol{12}}$ | $\boldsymbol{D}_{\boldsymbol{22}}$ | $\boldsymbol{D}_{\boldsymbol{12}}$ | $\boldsymbol{D}_{\boldsymbol{22}}$ | $\boldsymbol{D}_{\boldsymbol{12}}$ | $\boldsymbol{D}_{\boldsymbol{22}}$ | $\boldsymbol{D}_{\boldsymbol{12}}$ | $\boldsymbol{D}_{\boldsymbol{22}}$ | $\boldsymbol{D}_{\boldsymbol{12}}$ | $\boldsymbol{D}_{\boldsymbol{22}}$ | $\boldsymbol{D}_{\boldsymbol{12}}$ | $\boldsymbol{D}_{\boldsymbol{22}}$ | $\boldsymbol{D}_{\boldsymbol{12}}$ | $\boldsymbol{D}_{\boldsymbol{22}}$ |
| 0 | 0 | 0.315 | 0.316 | 0.122 | 0.122 | 0.08 | 0.08 | 0.14 | 0.13 | 0.05 | 0.06 | 0.41 | 0.41 | 15% | 15% |
| 0 | 0.2 | 0.315 | 0.315 | 0.137 | 0.137 | 0.07 | 0.07 | 0.11 | 0.11 | 0.05 | 0.05 | 0.54 | 0.54 | 43% | 43% |
| 0.2 | 0 | 0.313 | - | 0.214 | - | 0.10 | - | 1.01 | - | 0.05 | - | 0.73 | - | 44% | - |
| 0.2 | 0.2 | 0.311 | - | 0.225 | - | 0.11 | - | 0.98 | - | 0.05 | - | 0.76 | - | 58% | - |
| 2) Comparison of assigning therapists to patients *before* ${(D}_{22})$ randomising interventions to patients with blocking the randomisation of interventions to patients by therapists${(D}_{32})$ | | | | | | | | | | | | | | | |
|  | | $\boldsymbol{D}_{\boldsymbol{22}}$ | $\boldsymbol{D}_{\boldsymbol{32}}$ | $\boldsymbol{D}_{\boldsymbol{22}}$ | $\boldsymbol{D}_{\boldsymbol{32}}$ | $\boldsymbol{D}_{\boldsymbol{22}}$ | $\boldsymbol{D}_{\boldsymbol{32}}$ | $\boldsymbol{D}_{\boldsymbol{22}}$ | $\boldsymbol{D}_{\boldsymbol{32}}$ | $\boldsymbol{D}_{\boldsymbol{22}}$ | $\boldsymbol{D}_{\boldsymbol{32}}$ | $\boldsymbol{D}_{\boldsymbol{22}}$ | $\boldsymbol{D}_{\boldsymbol{32}}$ | $\boldsymbol{D}_{\boldsymbol{22}}$ | $\boldsymbol{D}_{\boldsymbol{32}}$ |
| 0 | 0 | 0.316 | 0.317 | 0.122 | 0.121 | 0.08 | 0.08 | 0.14 | 0.14 | 0.06 | 0.06 | 0.41 | 0.40 | 15% | 14% |
| 0 | 0.2 | 0.315 | 0.316 | 0.137 | 0.133 | 0.07 | 0.07 | 0.11 | 0.11 | 0.05 | 0.05 | 0.54 | 0.51 | 43% | 41% |
| 0.2 | 0 | - | - | - | - | - | - | - | - | - | - | - | - | - | - |
| 0.2 | 0.2 | - | - | - | - | - | - | - | - | - | - | - | - | - | - |
| 3) Comparison of blocking the randomisation of interventions to patients by therapists${(D}_{32})$ and randomising the therapist-intervention combination to patients (our proposed method) ${(D}_{42})$ | | | | | | | | | | | | | | | |
|  | | $\boldsymbol{D}_{\boldsymbol{32}}$ | $\boldsymbol{D}_{\boldsymbol{42}}$ | $\boldsymbol{D}_{\boldsymbol{32}}$ | $\boldsymbol{D}_{\boldsymbol{42}}$ | $\boldsymbol{D}_{\boldsymbol{32}}$ | $\boldsymbol{D}_{\boldsymbol{42}}$ | $\boldsymbol{D}_{\boldsymbol{32}}$ | $\boldsymbol{D}_{\boldsymbol{42}}$ | $\boldsymbol{D}_{\boldsymbol{32}}$ | $\boldsymbol{D}_{\boldsymbol{42}}$ | $\boldsymbol{D}_{\boldsymbol{32}}$ | $\boldsymbol{D}_{\boldsymbol{42}}$ | $\boldsymbol{D}_{\boldsymbol{32}}$ | $\boldsymbol{D}_{\boldsymbol{42}}$ |
| 0 | 0 | 0.317 | 0.314 | 0.121 | 0.121 | 0.08 | 0.08 | 0.14 | 0.14 | 0.06 | 0.05 | 0.40 | 0.40 | 14% | 14% |
| 0 | 0.2 | 0.316 | 0.319 | 0.133 | 0.142 | 0.07 | 0.08 | 0.11 | 0.10 | 0.05 | 0.05 | 0.51 | 0.55 | 41% | 38% |
| 0.2 | 0 | - | - | - | - | - | - | - | - | - | - | - | - | - | - |
| 0.2 | 0.2 | - | - | - | - | - | - | - | - | - | - | - | - | - | - |
| 4) Comparison of randomising the therapist-intervention combination to patients (our proposed method) ${(D}_{42})$ and randomising interventions to patients, then randomising therapists to patients ${(D}_{52})$ | | | | | | | | | | | | | | | |
|  |  | $\boldsymbol{D}_{\boldsymbol{42}}$ | $\boldsymbol{D}_{\boldsymbol{52}}$ | $\boldsymbol{D}_{\boldsymbol{42}}$ | $\boldsymbol{D}_{\boldsymbol{52}}$ | $\boldsymbol{D}_{\boldsymbol{42}}$ | $\boldsymbol{D}_{\boldsymbol{52}}$ | $\boldsymbol{D}_{\boldsymbol{42}}$ | $\boldsymbol{D}_{\boldsymbol{52}}$ | $\boldsymbol{D}_{\boldsymbol{42}}$ | $\boldsymbol{D}_{\boldsymbol{52}}$ | $\boldsymbol{D}_{\boldsymbol{42}}$ | $\boldsymbol{D}_{\boldsymbol{52}}$ | $\boldsymbol{D}_{\boldsymbol{42}}$ | $\boldsymbol{D}_{\boldsymbol{52}}$ |
| 0 | 0 | 0.314 | 0.314 | 0.121 | 0.122 | 0.08 | 0.08 | 0.14 | 0.14 | 0.05 | 0.05 | 0.40 | 0.40 | 14% | 14% |
| 0 | 0.2 | 0.319 | 0.316 | 0.142 | 0.229 | 0.08 | 0.08 | 0.10 | 0.11 | 0.05 | 0.05 | 0.55 | 0.80 | 38% | 41% |
| 0.2 | 0 | - | 0.315 | - | 0.142 | - | 0.07 | - | 0.10 | - | 0.05 | - | 0.56 | - | 41% |
| 0.2 | 0.2 | - | 0.314 | - | 0.163 | - | 0.07 | - | 0.09 | - | 0.05 | - | 0.63 | - | 58% |

Note: $\hat{\delta}_{1}$ is the fixed intervention effect, SE($\hat{\delta}_{1}$) is the standard error of $\hat{\delta}_{1}$, $\hat{\sigma}_{u1}^{2}$ is the between-therapist variance, $\hat{\sigma}_{v1}^{2}$ is the between-therapist

variance in the intervention effect, $\delta_{2}$ is the fixed coefficient of the baseline patient covariate associated with how therapists are allocated to patients

and $\delta_{3}$ is the fixed coefficient of baseline patient covariate associated with how therapists are allocated to patients with knowledge of the randomised

intervention. Boundary estimates are where $\sigma_{u1}^{2}$ or $\sigma_{v1}^{2}$ is estimated to be zero and a warning is given by the software. The model converged in every case.

**Table H2. Simulation results for the multicentre randomised block factorial design.**

| $\boldsymbol{\delta}_{\boldsymbol{3}}$ | $\boldsymbol{\delta}_{\boldsymbol{2}}$ | ${\hat{\boldsymbol{\delta}}}_{\boldsymbol{1}}$ | | **SE**$\boldsymbol{(}{\hat{\boldsymbol{\delta}}}_{\boldsymbol{1}}\boldsymbol{)}$ | | ${\hat{\boldsymbol{\sigma}}}_{\boldsymbol{u}\boldsymbol{1}}^{\boldsymbol{2}}$ | | ${\hat{\boldsymbol{\sigma}}}_{\boldsymbol{v}\boldsymbol{1}}^{\boldsymbol{2}}$ | | **Type I Error** | | **Type II Error** | | **Boundary Estimates** | |
| --- | --- | --- | --- | --- | --- | --- | --- | --- | --- | --- | --- | --- | --- | --- | --- |
| 1) Comparison of assigning therapists to patients *after* ${(D}_{13})$and *before* ${(D}_{23})$ randomising interventions to patients | | | | | | | | | | | | | | | |
|  | | $\boldsymbol{D}_{\boldsymbol{13}}$ | $\boldsymbol{D}_{\boldsymbol{23}}$ | $\boldsymbol{D}_{\boldsymbol{13}}$ | $\boldsymbol{D}_{\boldsymbol{23}}$ | $\boldsymbol{D}_{\boldsymbol{13}}$ | $\boldsymbol{D}_{\boldsymbol{23}}$ | $\boldsymbol{D}_{\boldsymbol{13}}$ | $\boldsymbol{D}_{\boldsymbol{23}}$ | $\boldsymbol{D}_{\boldsymbol{13}}$ | $\boldsymbol{D}_{\boldsymbol{23}}$ | $\boldsymbol{D}_{\boldsymbol{13}}$ | $\boldsymbol{D}_{\boldsymbol{23}}$ | $\boldsymbol{D}_{\boldsymbol{13}}$ | $\boldsymbol{D}_{\boldsymbol{23}}$ |
| 0 | 0 | 0.180 | 0.181 | 0.136 | 0.135 | 0.09 | 0.09 | 0.10 | 0.10 | 0.05 | 0.05 | 0.80 | 0.79 | 0% | 1% |
| 0 | 0.2 | 0.181 | 0.182 | 0.140 | 0.140 | 0.05 | 0.05 | 0.08 | 0.08 | 0.04 | 0.05 | 0.82 | 0.82 | 24% | 26% |
| 0.2 | 0 | 0.179 | - | 0.172 | - | 0.08 | - | 1.04 | - | 0.06 | - | 0.85 | - | 33% | - |
| 0.2 | 0.2 | 0.181 | - | 0.177 | - | 0.07 | - | 1.01 | - | 0.05 | - | 0.86 | - | 40% | - |
| 2) Comparison of assigning therapists to patients *before* ${(D}_{23})$ randomising interventions to patients with blocking the randomisation of interventions to patients by therapists${(D}_{33})$ | | | | | | | | | | | | | | | |
|  | | $\boldsymbol{D}_{\boldsymbol{23}}$ | $\boldsymbol{D}_{\boldsymbol{33}}$ | $\boldsymbol{D}_{\boldsymbol{23}}$ | $\boldsymbol{D}_{\boldsymbol{33}}$ | $\boldsymbol{D}_{\boldsymbol{23}}$ | $\boldsymbol{D}_{\boldsymbol{33}}$ | $\boldsymbol{D}_{\boldsymbol{23}}$ | $\boldsymbol{D}_{\boldsymbol{33}}$ | $\boldsymbol{D}_{\boldsymbol{23}}$ | $\boldsymbol{D}_{\boldsymbol{33}}$ | $\boldsymbol{D}_{\boldsymbol{23}}$ | $\boldsymbol{D}_{\boldsymbol{33}}$ | $\boldsymbol{D}_{\boldsymbol{23}}$ | $\boldsymbol{D}_{\boldsymbol{33}}$ |
| 0 | 0 | 0.181 | 0.181 | 0.135 | 0.135 | 0.09 | 0.10 | 0.10 | 0.10 | 0.05 | 0.05 | 0.79 | 0.80 | 1% | 0% |
| 0 | 0.2 | 0.182 | 0.182 | 0.139 | 0.139 | 0.05 | 0.05 | 0.08 | 0.09 | 0.05 | 0.04 | 0.82 | 0.81 | 26% | 23% |
| 0.2 | 0 | - | - | - | - | - | - | - | - | - | - | - | - | - | - |
| 0.2 | 0.2 | - | - | - | - | - | - | - | - | - | - | - | - | - | - |
| 3) Comparison of blocking the randomisation of interventions to patients by therapists${(D}_{33})$ and randomising the therapist-intervention combination to patients (our proposed method) ${(D}_{43})$ | | | | | | | | | | | | | | | |
|  | | $\boldsymbol{D}_{\boldsymbol{33}}$ | $\boldsymbol{D}_{\boldsymbol{43}}$ | $\boldsymbol{D}_{\boldsymbol{33}}$ | $\boldsymbol{D}_{\boldsymbol{43}}$ | $\boldsymbol{D}_{\boldsymbol{33}}$ | $\boldsymbol{D}_{\boldsymbol{43}}$ | $\boldsymbol{D}_{\boldsymbol{33}}$ | $\boldsymbol{D}_{\boldsymbol{43}}$ | $\boldsymbol{D}_{\boldsymbol{33}}$ | $\boldsymbol{D}_{\boldsymbol{43}}$ | $\boldsymbol{D}_{\boldsymbol{33}}$ | $\boldsymbol{D}_{\boldsymbol{43}}$ | $\boldsymbol{D}_{\boldsymbol{33}}$ | $\boldsymbol{D}_{\boldsymbol{43}}$ |
| 0 | 0 | 0.181 | 0.180 | 0.135 | 0.135 | 0.10 | 0.09 | 0.10 | 0.10 | 0.05 | 0.05 | 0.80 | 0.80 | 0% | 0% |
| 0 | 0.2 | 0.182 | 0.180 | 0.139 | 0.140 | 0.05 | 0.09 | 0.09 | 0.09 | 0.04 | 0.04 | 0.81 | 0.82 | 23% | 11% |
| 0.2 | 0 | - | - | - | - | - | - | - | - | - | - | - | - | - | - |
| 0.2 | 0.2 | - | - | - | - | - | - | - | - | - | - | - | - | - | - |
| 4) Comparison of randomising the therapist-intervention combination to patients (our proposed method) ${(D}_{43})$ and randomising interventions to patients, then randomising therapists to patients ${(D}_{53})$ | | | | | | | | | | | | | | | |
|  |  | $\boldsymbol{D}_{\boldsymbol{43}}$ | $\boldsymbol{D}_{\boldsymbol{53}}$ | $\boldsymbol{D}_{\boldsymbol{43}}$ | $\boldsymbol{D}_{\boldsymbol{53}}$ | $\boldsymbol{D}_{\boldsymbol{43}}$ | $\boldsymbol{D}_{\boldsymbol{53}}$ | $\boldsymbol{D}_{\boldsymbol{43}}$ | $\boldsymbol{D}_{\boldsymbol{53}}$ | $\boldsymbol{D}_{\boldsymbol{43}}$ | $\boldsymbol{D}_{\boldsymbol{53}}$ | $\boldsymbol{D}_{\boldsymbol{43}}$ | $\boldsymbol{D}_{\boldsymbol{53}}$ | $\boldsymbol{D}_{\boldsymbol{43}}$ | $\boldsymbol{D}_{\boldsymbol{53}}$ |
| 0 | 0 | 0.180 | 0.179 | 0.135 | 0.135 | 0.09 | 0.09 | 0.10 | 0.10 | 0.05 | 0.05 | 0.80 | 0.80 | 0% | 0% |
| 0 | 0.2 | 0.180 | 0.183 | 0.140 | 0.159 | 0.09 | 0.09 | 0.09 | 0.09 | 0.04 | 0.04 | 0.82 | 0.86 | 11% | 16% |
| 0.2 | 0 | - | 0.179 | - | 0.142 | - | 0.08 | - | 0.08 | - | 0.05 | - | 0.84 | - | 17% |
| 0.2 | 0.2 | - | 0.180 | - | 0.149 | - | 0.08 | - | 0.07 | - | 0.04 | - | 0.85 | - | 37% |

Note: $\hat{\delta}_{1}$ is the fixed intervention effect, SE($\hat{\delta}_{1}$) is the standard error of $\hat{\delta}_{1}$, $\hat{\sigma}_{u1}^{2}$ is the between-therapist variance, $\hat{\sigma}_{v1}^{2}$ is the between-therapist

variance in the intervention effect, $\delta_{2}$ is the fixed coefficient of the baseline patient covariate associated with how therapists are allocated to patients

and $\delta_{3}$ is the fixed coefficient of baseline patient covariate associated with how therapists are allocated to patients with knowledge of the randomised

intervention. Boundary estimates are where $\sigma_{u1}^{2}$ or $\sigma_{v1}^{2}$ is estimated to be zero and a warning is given by the software. The model converged in every case
