# Supplementary material for: Orthogonal Factorial Designs for Trials of Therapist‐Delivered Interventions: Randomizing Intervention‐Therapist Combinations to Patients
Source: Stat Med. 2026 Nov 10;45(25-27):e70813. doi: 10.1002/sim.70813 (PMC13749633; doi:10.1002/sim.70813)
Supplement: Supplementary file 2 [file SIM-45-0-s002.docx]

**Supplementary File D – Eigenvalues of** $Cov\left( \boldsymbol{Y} \right)$

1. **Completely Randomised Factorial Design**

The variance-covariance matrix of **Y** can be written as

$Cov\left( \boldsymbol{Y} \right)=\sigma_{0}^{2}\boldsymbol{J+}\sigma_{T}^{2}\boldsymbol{J}_{T}+\sigma_{I\wedge T}^{2}\boldsymbol{J}_{I\wedge T}+\sigma^{2}\boldsymbol{I}$	(D.1)

where $\boldsymbol{J}$ is the $N\times N$ matrix of ones, $\boldsymbol{J}_{T}$ is the $N\times N$ matrix whose entry is equal to one if any two patients receive treatment from the same therapist but zero otherwise, and $\boldsymbol{J}_{I\wedge T}$ is the $N\times N$ matrix whose entry is equal to one if any two patients receive the same intervention from the same therapist but zero otherwise.

The matrix $n_{I}^{-1}n_{T}^{-1}n_{R}^{-1}\boldsymbol{J}$ is equal to the matrix $\boldsymbol{P}_{V_{0}}$ of orthogonal projection onto the subspace $V_{0}$. As $V_{0}=W_{0},$ we may replace $\sigma_{0}^{2}\boldsymbol{J}$ in (D.1) by $\sigma_{0}^{2}{n_{I}n_{T}n_{R}\boldsymbol{P}}_{W_{0}}$. The matrix ${n_{I}^{-1}n_{R}^{-1}\boldsymbol{J}}_{T}$ is equal to the matrix $\boldsymbol{P}_{V_{T}}$ of orthogonal projection onto the subspace $V_{T}$. As $V_{T}=W_{0}\bigoplus W_{T}$, we may replace $\sigma_{T}^{2}\boldsymbol{J}_{T}$ in (D.1) by ${\sigma_{T}^{2}n_{I}n_{R}\boldsymbol{(P}}_{W_{0}}+\boldsymbol{P}_{W_{T}})$. The matrix $n_{R}^{-1}\boldsymbol{J}_{I\wedge T}$ is equal to matrix $\boldsymbol{P}_{V_{I\wedge T}}$ of orthogonal projection onto $V_{I\wedge T}$. As $V_{I\wedge T}=$ $W_{0}\bigoplus W_{T}\bigoplus W_{I\wedge T}$, $\sigma_{I\wedge T}^{2}\boldsymbol{J}_{I\wedge T}$ in (D.1) can be replaced by ${\sigma_{I\wedge T}^{2}n_{R}\boldsymbol{(P}}_{W_{0}}+\boldsymbol{P}_{W_{T}}+\boldsymbol{P}_{W_{I\wedge T}})$. $\boldsymbol{I}$ is equal to the matrix $\boldsymbol{P}_{V}$ of orthogonal projection onto whole space $V$. As $V=W_{0}\bigoplus W_{T}\bigoplus W_{I\wedge T}\bigoplus W_{E}$, $\sigma^{2}\boldsymbol{I}$ can be replaced by $\sigma^{2}\boldsymbol{(P}_{W_{0}}+\boldsymbol{P}_{W_{T}}+\boldsymbol{P}_{W_{I\wedge T}}+\boldsymbol{P}_{W_{E}}),$ and (D.1) can be re-written as

$$Cov\left( \boldsymbol{Y} \right)={\sigma_{0}^{2}{n_{I}n_{T}n_{R}\boldsymbol{P}}_{W_{0}}+\sigma_{T}^{2}n_{I}n_{R}\boldsymbol{(P}}_{W_{0}}+\boldsymbol{P}_{W_{T}})+{\sigma_{I\wedge T}^{2}n_{R}\boldsymbol{(P}}_{W_{0}}+\boldsymbol{P}_{W_{T}}+\boldsymbol{P}_{W_{I\wedge T}})$$

$$+\sigma^{2}\boldsymbol{(P}_{W_{0}}+\boldsymbol{P}_{W_{T}}+\boldsymbol{P}_{W_{I\wedge T}}+\boldsymbol{P}_{W_{E}})$$

$$={({\sigma_{0}^{2}n}_{I}n_{T}n_{R}+\sigma_{T}^{2}n_{I}n_{R}\boldsymbol{+}\sigma_{I\wedge T}^{2}n_{R}+\sigma^{2}\boldsymbol{)P}}_{W_{0}}+{\left( \sigma_{T}^{2}n_{I}n_{R}\boldsymbol{+}\sigma_{I\wedge T}^{2}n_{R}+\sigma^{2} \right)\boldsymbol{P}}_{W_{T}} +\left( \sigma_{I\wedge T}^{2}n_{R}+\sigma^{2} \right)\boldsymbol{P}_{W_{I\wedge T}}+\sigma^{2}\boldsymbol{P}_{W_{E}}$$

$=\xi_{0}\boldsymbol{P}_{W_{0}}+\xi_{T}\boldsymbol{P}_{W_{T}}+\xi_{I\wedge T}\boldsymbol{P}_{W_{I\wedge T}}+\xi_{E}\boldsymbol{P}_{W_{E}}$	(D.2)

where the $\xi$ are the eigenvalues of $Cov\left( \boldsymbol{Y} \right)$. There are four eigenvalues, $\xi_{0}, \xi_{T}$, $\xi_{I\wedge T}$ and $\xi_{E}$ that relate to the mean, to therapists, to treatments and to patients, respectively.

2. **Randomised Block Factorial Design**

Here, the variance-covariance matrix of **Y** can be written as

$Cov\left( \boldsymbol{Y} \right)=\sigma_{0}^{2}\boldsymbol{J}+\sigma_{T}^{2}\boldsymbol{J}_{T}+\sigma_{B}^{2}\boldsymbol{J}_{B}+\sigma_{I\wedge T}^{2}\boldsymbol{J}_{I\wedge T}+\sigma_{I\wedge B}^{2}\boldsymbol{J}_{I\wedge B}+\sigma_{T\wedge B}^{2}\boldsymbol{J}_{T\wedge B}+\sigma_{I\wedge T\wedge B}^{2}\boldsymbol{J}_{I\wedge T\wedge B}+\sigma^{2}\boldsymbol{I}\boldsymbol{.}$	(D.3)

Note the absence of $\sigma_{I}^{2}\boldsymbol{J}_{I}$ follows because $I$ is a fixed factor, not a random factor, and the dependent random effect of $\left( I\wedge T \right)\vee\left( I\wedge B \right)$ does not contribute any new variance parameters. Replacing each term on the right of (D.3), with the matrices of orthogonal projection, using Figure B4, gives

$$Cov\left( \boldsymbol{Y} \right)={{\sigma_{0}^{2}{n_{I}n_{T}n_{B}n_{R}\boldsymbol{P}}_{W_{0}}+\sigma}_{T}^{2}n_{I}n_{B}n_{R}\boldsymbol{(P}}_{W_{0}}+\boldsymbol{P}_{W_{T}}) +{\sigma_{B}^{2}n_{I}n_{T}n_{R}\boldsymbol{(P}}_{W_{0}}+\boldsymbol{P}_{W_{B}}) +{\sigma_{I\wedge T}^{2}n_{B}n_{R}\boldsymbol{(P}}_{W_{0}}+\boldsymbol{P}_{W_{I}}+\boldsymbol{P}_{W_{T}}+\boldsymbol{P}_{W_{I\wedge T}}) +{\sigma_{I\wedge B}^{2}n_{T}n_{R}\boldsymbol{(P}}_{W_{0}}+\boldsymbol{P}_{W_{I}}+\boldsymbol{P}_{W_{B}}+\boldsymbol{P}_{W_{I\wedge B}}) +{\sigma_{T\wedge B}^{2}n_{I}n_{R}\boldsymbol{(P}}_{W_{0}}+\boldsymbol{P}_{W_{T}}+\boldsymbol{P}_{W_{B}}+\boldsymbol{P}_{W_{T\wedge B}}) +\sigma_{I\wedge T\wedge B}^{2}n_{R}\boldsymbol{(P}_{W_{0}}+\boldsymbol{P}_{W_{I}}+\boldsymbol{P}_{W_{T}}+\boldsymbol{P}_{W_{B}}+\boldsymbol{P}_{W_{I\wedge T}}+\boldsymbol{P}_{W_{I\wedge B}}+\boldsymbol{P}_{W_{T\wedge B}}+\boldsymbol{P}_{W_{I\wedge T\wedge B}})$$

$$+\sigma^{2}\boldsymbol{(P}_{W_{0}}+\boldsymbol{P}_{W_{I}}+\boldsymbol{P}_{W_{T}}+\boldsymbol{P}_{W_{B}}+\boldsymbol{P}_{W_{I\wedge T}}+\boldsymbol{P}_{W_{I\wedge B}}+\boldsymbol{P}_{W_{T\wedge B}}+\boldsymbol{P}_{W_{I\wedge T\wedge B}}+\boldsymbol{P}_{W_{E}})$$

$$={\left( \sigma_{0}^{2}n_{I}n_{T}n_{B}n_{R}+\sigma_{T}^{2}n_{I}n_{B}n_{R}+\sigma_{B}^{2}n_{I}n_{T}n_{R}\boldsymbol{+}\sigma_{I\wedge T}^{2}n_{B}n_{R}\boldsymbol{+}\sigma_{I\wedge B}^{2}n_{T}n_{R}\boldsymbol{+}\sigma_{T\wedge B}^{2}n_{I}n_{R} \right)\boldsymbol{P}}_{W_{0}} +(\sigma_{I\wedge T\wedge B}^{2}n_{R}+\sigma^{2})\boldsymbol{P}_{W_{0}}+{\boldsymbol{(}\sigma_{I\wedge T}^{2}n_{B}n_{R}\boldsymbol{+}\sigma_{I\wedge B}^{2}n_{T}n_{R}+\sigma_{I\wedge T\wedge B}^{2}n_{R}+\sigma^{2}\boldsymbol{)P}}_{W_{I}}+{\boldsymbol{(}\sigma_{T}^{2}n_{I}n_{B}n_{R}\boldsymbol{+}\sigma_{I\wedge T}^{2}n_{B}n_{R}\boldsymbol{+}\sigma_{T\wedge B}^{2}n_{I}n_{R}+\sigma_{I\wedge T\wedge B}^{2}n_{R}+\sigma^{2}\boldsymbol{)P}}_{W_{T}}+\left( \sigma_{B}^{2}n_{I}n_{T}n_{R}+\sigma_{I\wedge B}^{2}n_{T}n_{R}+\sigma_{T\wedge B}^{2}n_{I}n_{R}+\sigma_{I\wedge T\wedge B}^{2}n_{R}+\sigma^{2} \right)\boldsymbol{P}_{W_{B}}+\left( \sigma_{I\wedge T}^{2}n_{B}n_{R}+\sigma_{I\wedge T\wedge B}^{2}n_{R}+\sigma^{2} \right)\boldsymbol{P}_{W_{I\wedge T}}+\left( \sigma_{I\wedge B}^{2}n_{T}n_{R}+\sigma_{I\wedge T\wedge B}^{2}n_{R}+\sigma^{2} \right)\boldsymbol{P}_{W_{I\wedge B}}+\left( \sigma_{T\wedge B}^{2}n_{I}n_{R}+\sigma_{I\wedge T\wedge B}^{2}n_{R}+\sigma^{2} \right)\boldsymbol{P}_{W_{T\wedge B}}+\left( \sigma_{I\wedge T\wedge B}^{2}n_{R}+\sigma^{2} \right)\boldsymbol{P}_{W_{I\wedge T\wedge B}}+\sigma^{2}\boldsymbol{P}_{W_{E}}$$

$$=\xi_{0}\boldsymbol{P}_{W_{0}}+\xi_{I}\boldsymbol{P}_{W_{I}}+\xi_{T}\boldsymbol{P}_{W_{T}}+\xi_{B}\boldsymbol{P}_{W_{B}}+\xi_{I\wedge T}\boldsymbol{P}_{W_{I\wedge T}}+\xi_{I\wedge B}\boldsymbol{P}_{W_{I\wedge B}}+\xi_{T\wedge B}\boldsymbol{P}_{W_{T\wedge B}}$$

$+\xi_{I\wedge T\wedge B}\boldsymbol{P}_{W_{I\wedge T\wedge B}}+\xi_{E}\boldsymbol{P}_{W_{E}}$	(D.4)

where, as before, the $\xi$ are the eigenvalues of $Cov\left( \boldsymbol{Y} \right)$. There are nine eigenvalues: $\xi_{0},$ $\xi_{I},$ $\xi_{T}, \xi_{B}, \xi_{I\wedge T},$ $\xi_{I\wedge B}$, $\xi_{T\wedge B}$, $\xi_{I\wedge T\wedge B}$ and $\xi_{E}$ that relate to the mean, to interventions, therapists, batches, treatments, intervention-by-batch combinations, therapist-by-batch combinations, intervention-by-therapist-by-batch combinations and patients, respectively.

3. **Multicentre Randomised Block Factorial Design**

Here, the variance-covariance matrix of **Y** can be written as

$$Cov\left( \boldsymbol{Y} \right)=\sigma_{0}^{2}\boldsymbol{J}+\sigma_{C}^{2}\boldsymbol{J}_{C}+{\sigma_{B}^{2}\boldsymbol{J}_{B}+\sigma}_{I\wedge C}^{2}\boldsymbol{J}_{I\wedge C}+\sigma_{T}^{2}\boldsymbol{J}_{T}+\sigma_{I\wedge B}^{2}\boldsymbol{J}_{I\wedge B}+\sigma_{C\wedge B}^{2}\boldsymbol{J}_{C\wedge B}+\sigma_{I\wedge T}^{2}\boldsymbol{J}_{I\wedge T}$$

$+{\sigma_{I\wedge C\wedge B}^{2}\boldsymbol{J}}_{I\wedge C\wedge B}+\sigma_{T\wedge B}^{2}\boldsymbol{J}_{T\wedge B}+\sigma_{I\wedge T\wedge B}^{2}\boldsymbol{J}_{I\wedge T\wedge B}+\sigma^{2}\boldsymbol{I}$ . (D.5)

Note again the absence of $\sigma_{I}^{2}\boldsymbol{J}_{I}$. Replacing each term on the right of (D.5), with the matrices of orthogonal projection, using Figure B5, gives

$$Cov\left( \boldsymbol{Y} \right)={{\sigma_{0}^{2}n_{I}{n_{C}n_{B}n}_{T}n_{R}\boldsymbol{P}}_{W_{0}}+{{\sigma_{C}^{2}n_{I}n_{B}n_{T}n_{R}\boldsymbol{(P}}_{W_{0}}+\boldsymbol{P}_{W_{C}})+{\sigma_{B}^{2}n_{I}{n_{C}n}_{T}n_{R}\boldsymbol{(P}}_{W_{0}}+\boldsymbol{P}_{W_{B}}) +{\sigma_{I\wedge C}^{2}n_{B}n_{T}n_{R}\boldsymbol{(P}}_{W_{0}}+\boldsymbol{P}_{W_{I}}+\boldsymbol{P}_{W_{C}}+\boldsymbol{P}_{W_{I\wedge C}})+\sigma}_{T}^{2}n_{I}n_{B}n_{R}\boldsymbol{(P}}_{W_{0}}+\boldsymbol{P}_{W_{C}}+\boldsymbol{P}_{W_{T}})+{\sigma_{I\wedge B}^{2}{n_{C}n}_{T}n_{R}\boldsymbol{(P}}_{W_{0}}+\boldsymbol{P}_{W_{I}}+\boldsymbol{P}_{W_{B}}+\boldsymbol{P}_{W_{I\wedge B}}) +{{\sigma_{C\wedge B}^{2}n}_{I}n_{T}n_{R}\boldsymbol{(P}}_{W_{0}}+\boldsymbol{P}_{W_{C}}+\boldsymbol{P}_{W_{B}}+\boldsymbol{P}_{W_{C\wedge B}}) +{\sigma_{I\wedge T}^{2}n_{B}n_{R}\boldsymbol{(P}}_{W_{0}}+\boldsymbol{P}_{W_{I}}+\boldsymbol{P}_{W_{C}}+\boldsymbol{P}_{W_{I\wedge C}}+\boldsymbol{P}_{W_{T}}+\boldsymbol{P}_{W_{I\wedge T}}) +{\sigma_{I\wedge C\wedge B}^{2}n_{T}n_{R}\boldsymbol{(P}}_{W_{0}}+\boldsymbol{P}_{W_{I}}+\boldsymbol{P}_{W_{C}}+\boldsymbol{P}_{W_{B}}+\boldsymbol{P}_{W_{I\wedge C}}+\boldsymbol{P}_{W_{I\wedge B}}+\boldsymbol{P}_{W_{C\wedge B}}+\boldsymbol{P}_{W_{I\wedge C\wedge B}})+{\sigma_{T\wedge B}^{2}n_{I}n_{R}\boldsymbol{(P}}_{W_{0}}+\boldsymbol{P}_{W_{C}}+\boldsymbol{P}_{W_{B}}+\boldsymbol{P}_{W_{T}}+\boldsymbol{P}_{W_{C\wedge B}}+\boldsymbol{P}_{W_{T\wedge B}}) +\sigma_{I\wedge T\wedge B}^{2}n_{R}\boldsymbol{(P}_{W_{0}}+\boldsymbol{P}_{W_{I}}+\boldsymbol{P}_{W_{C}}+\boldsymbol{P}_{W_{B}}+\boldsymbol{P}_{W_{I\wedge C}}+\boldsymbol{P}_{W_{T}}+\boldsymbol{P}_{W_{I\wedge B}}+\boldsymbol{P}_{W_{C\wedge B}}+\boldsymbol{P}_{W_{I\wedge T}}+\boldsymbol{P}_{W_{I\wedge C\wedge B}}+\boldsymbol{P}_{W_{T\wedge B}}+\boldsymbol{P}_{W_{I\wedge T\wedge B}})+\sigma^{2}\boldsymbol{(P}_{W_{0}}+\boldsymbol{P}_{W_{I}}+\boldsymbol{P}_{W_{C}}+\boldsymbol{P}_{W_{B}}+\boldsymbol{P}_{W_{I\wedge C}}+\boldsymbol{P}_{W_{T}}+\boldsymbol{P}_{W_{I\wedge B}}+\boldsymbol{P}_{W_{C\wedge B}}+\boldsymbol{P}_{W_{I\wedge T}}+\boldsymbol{P}_{W_{I\wedge C\wedge B}}+\boldsymbol{P}_{W_{T\wedge B}}+\boldsymbol{P}_{W_{I\wedge T\wedge B}}+\boldsymbol{P}_{W_{E}})$$

$$={\left( \sigma_{0}^{2}n_{I}{n_{C}n_{B}n}_{T}n_{R}+\sigma_{C}^{2}n_{I}n_{B}n_{T}n_{R}+\sigma_{B}^{2}n_{I}{n_{C}n}_{T}n_{R}+\sigma_{I\wedge C}^{2}n_{B}n_{T}n_{R}+\sigma_{T}^{2}n_{I}n_{B}n_{R}+\sigma_{I\wedge B}^{2}{n_{C}n}_{T}n_{R}+\sigma_{C\wedge B}^{2}n_{I}n_{T}n_{R}+\sigma_{I\wedge T}^{2}n_{B}n_{R}+\sigma_{I\wedge C\wedge B}^{2}n_{T}n_{R}+\sigma_{T\wedge B}^{2}n_{I}n_{R}+\sigma_{I\wedge T\wedge B}^{2}n_{R}+\sigma^{2} \right)\boldsymbol{P}}_{W_{0}}$$

$$+{\left( {\sigma_{I\wedge C}^{2}n_{B}n_{T}n_{R}+\sigma_{I\wedge B}^{2}{n_{C}n}_{T}n_{R}+\sigma}_{I\wedge T}^{2}n_{B}n_{R}\boldsymbol{+}\sigma_{I\wedge C\wedge B}^{2}n_{T}n_{R}\boldsymbol{+}\sigma_{I\wedge T\wedge B}^{2}n_{R}\boldsymbol{+}\sigma^{2} \right)\boldsymbol{P}}_{W_{I}}$$

$$+{\left( {\sigma_{C}^{2}n_{I}n_{B}n_{T}n_{R}+\sigma_{I\wedge C}^{2}n_{B}n_{T}n_{R}+\sigma}_{T}^{2}n_{I}n_{B}n_{R}\boldsymbol{+}\sigma_{C\wedge B}^{2}n_{I}n_{T}n_{R}\boldsymbol{+}\sigma_{I\wedge T}^{2}n_{B}n_{R}\boldsymbol{+}\sigma_{I\wedge C\wedge B}^{2}n_{T}n_{R}\boldsymbol{+}\sigma_{T\wedge B}^{2}n_{I}n_{R}\boldsymbol{+}\sigma_{I\wedge T\wedge B}^{2}n_{R}\boldsymbol{+}\sigma^{2} \right)\boldsymbol{P}}_{W_{C}}$$

$$+\left( \sigma_{B}^{2}n_{I}{n_{C}n}_{T}n_{R}+\sigma_{I\wedge B}^{2}{n_{C}n}_{T}n_{R}+\sigma_{C\wedge B}^{2}n_{I}n_{T}n_{R}+\sigma_{I\wedge C\wedge B}^{2}n_{T}n_{R}+\sigma_{T\wedge B}^{2}n_{I}n_{R}+\sigma_{I\wedge T\wedge B}^{2}n_{R}+\sigma^{2} \right)\boldsymbol{P}_{W_{B}}$$

$$+\left( \sigma_{I\wedge C}^{2}n_{B}n_{T}n_{R}+\sigma_{I\wedge T}^{2}n_{B}n_{R}+\sigma_{I\wedge C\wedge B}^{2}n_{T}n_{R}+\sigma_{I\wedge T\wedge B}^{2}n_{R}+\sigma^{2} \right)\boldsymbol{P}_{W_{I\wedge C}}$$

$$+\left( \sigma_{T}^{2}n_{I}n_{B}n_{R}+\sigma_{I\wedge T}^{2}n_{B}n_{R}+\sigma_{T\wedge B}^{2}n_{I}n_{R}+\sigma_{I\wedge T\wedge B}^{2}n_{R}+\sigma^{2} \right)\boldsymbol{P}_{W_{T}}$$

$$+\left( \sigma_{I\wedge B}^{2}{n_{C}n}_{T}n_{R}+\sigma_{I\wedge C\wedge B}^{2}n_{T}n_{R}+\sigma_{I\wedge T\wedge B}^{2}n_{R}+\sigma^{2} \right)\boldsymbol{P}_{W_{I\wedge B}}$$

$$+\left( \sigma_{C\wedge B}^{2}n_{I}n_{T}n_{R}+\sigma_{I\wedge C\wedge B}^{2}n_{T}n_{R}+\sigma_{T\wedge B}^{2}n_{I}n_{R}+\sigma_{I\wedge T\wedge B}^{2}n_{R}+\sigma^{2} \right)\boldsymbol{P}_{W_{C\wedge B}}$$

$$+\left( \sigma_{I\wedge T}^{2}n_{B}n_{R}+\sigma_{I\wedge T\wedge B}^{2}n_{R}+\sigma^{2} \right)\boldsymbol{P}_{W_{I\wedge T}}$$

$$+\left( \sigma_{I\wedge C\wedge B}^{2}n_{T}n_{R}+\sigma_{I\wedge T\wedge B}^{2}n_{R}+\sigma^{2} \right)\boldsymbol{P}_{W_{I\wedge C\wedge B}}+\left( \sigma_{T\wedge B}^{2}n_{I}n_{R}+\sigma_{I\wedge T\wedge B}^{2}n_{R}+\sigma^{2} \right)\boldsymbol{P}_{W_{T\wedge B}}$$

$$+\left( \sigma_{I\wedge T\wedge B}^{2}n_{R}+\sigma^{2} \right)\boldsymbol{P}_{W_{I\wedge T\wedge B}}$$

$$+\sigma^{2}\boldsymbol{P}_{W_{E}}$$

$$=\xi_{0}\boldsymbol{P}_{W_{0}}+\xi_{I}\boldsymbol{P}_{W_{I}}+\xi_{C}\boldsymbol{P}_{W_{C}}+\xi_{B}\boldsymbol{P}_{W_{B}}+\xi_{I\wedge C}\boldsymbol{P}_{W_{I\wedge C}}+\xi_{T}\boldsymbol{P}_{W_{T}}+\xi_{I\wedge B}\boldsymbol{P}_{W_{I\wedge B}}+\xi_{C\wedge B}\boldsymbol{P}_{W_{C\wedge B}}+$$

$\xi_{I\wedge T}\boldsymbol{P}_{W_{I\wedge T}}+\xi_{I\wedge C\wedge B}\boldsymbol{P}_{W_{I\wedge C\wedge B}}+\xi_{T\wedge B}\boldsymbol{P}_{W_{T\wedge B}}+\xi_{I\wedge T\wedge B}\boldsymbol{P}_{W_{I\wedge T\wedge B}}+\xi_{E}\boldsymbol{P}_{W_{E}}$	(D.6)

where the $\xi$ are the thirteen eigenvalues of $Cov\left( \boldsymbol{Y} \right)$: $\xi_{0}$, $\xi_{I}$, $\xi_{C}$, $\xi_{B}$, $\xi_{I\wedge C}$, $\xi_{T}$, $\xi_{I\wedge B}$, $\xi_{C\wedge B}$, $\xi_{I\wedge T}$, $\xi_{I\wedge C\wedge B}$, $\xi_{T\wedge B}$, $\xi_{I\wedge T\wedge B}$ and $\xi_{E}$ relating to the mean, to interventions, centres, batches, intervention-by-centre combinations, therapists, intervention-by-batch combinations, centre-by-batch combinations, treatments, intervention-by-centre-by-batch combinations, therapist-by-batch combinations, intervention-by-therapist-by-batch combinations and patients, respectively.
