# Supplementary material for: Orthogonal Factorial Designs for Trials of Therapist‐Delivered Interventions: Randomizing Intervention‐Therapist Combinations to Patients
Source: Stat Med. 2026 Nov 10;45(25-27):e70813. doi: 10.1002/sim.70813 (PMC13749633; doi:10.1002/sim.70813)
Supplement: Supplementary file 3 [file SIM-45-0-s003.docx]

**Supplementary File C – Combined Hasse Diagrams**

**Figure C1: Combined Hasse diagram for the completely randomised factorial design**

$U$	1

$I$	$n_{I}$	$T$	$n_{T}$

$I\wedge T$	$n_{I}n_{T}$

$E$	$n_{I}n_{T}n_{R}$

**Figure C2: Combined Hasse diagram for the randomised block factorial design**

$U$	1

$I$	$n_{I}$	$T$	$n_{T}$	$B n_{B}$

$I\wedge T$	$n_{I}n_{T}$	$I\wedge B n_{I}n_{B} T\wedge B$	$n_{T}n_{B}$

$I\wedge T\wedge B$	$n_{I}n_{T}n_{B}$

$E$	$n_{I}n_{T}{n_{B}n}_{R}$

**Figure C3: Combined Hasse diagram for the multicentre randomised block factorial design**

$U$	1

$I$	$n_{I}$	$C$	$n_{C}$	$B$	$n_{B}$

$I\wedge C$	$T$	$I\wedge B$	$C\wedge B$

$n_{I}n_{C}$	${n_{C}n}_{T}$	$n_{I}n_{B}$	$n_{C}n_{B}$

$I\wedge T$	$I\wedge C\wedge B T\wedge B$

$n_{I}n_{C}n_{T} n_{I}{n_{C}n}_{B}$	$n_{C}n_{T}n_{B}$

$I\wedge T\wedge B$	$n_{I}n_{C}n_{T}n_{B}$

$E$	$n_{I}{n_{C}n}_{T}n_{B}n_{R}$

Note that the Universal factor $U$ is given a special symbol in the combined Hasse diagrams to reflect it appearing in both the fixed and random structures. Factor $I$ is given a special symbol in Figures C2 and C3 to distinguish it both from a more traditional random or fixed factor.
