# Supplementary material for: Orthogonal Factorial Designs for Trials of Therapist‐Delivered Interventions: Randomizing Intervention‐Therapist Combinations to Patients
Source: Stat Med. 2026 Nov 10;45(25-27):e70813. doi: 10.1002/sim.70813 (PMC13749633; doi:10.1002/sim.70813)
Supplement: Supplementary file 4 [file SIM-45-0-s004.docx]

**Supplementary File A – Hasse diagrams for the random and fixed structures**

**Figure A1: Completely-randomised factorial design.**

$U$	1, 1	$U$	1, 1

$T$	$n_{T}, n_{T}-1$

$I\wedge T$	$n_{I}n_{T}, n_{T}(n_{I}-1)$	$I$	$n_{I}, n_{I}-1$

$E$	$n_{I}n_{T}n_{R}$, $n_{I}n_{T}(n_{R}-1)$

(i) Random structure	(ii) Fixed structure

**Figure A2: Randomised block factorial design.**

$U$	1, 1	$U$	1, 1

$I$	$T$	$n_{T}, n_{T}-1$	$B$

$n_{I}, n_{I}-1$	$n_{B}, n_{B}-1$

$I\wedge T$	$I\wedge B n_{I}n_{B},$	$T\wedge B$	$I$	$n_{I}, n_{I}-1$

$n_{I}n_{T},$	$\left( n_{I}-1 \right)\left( n_{B}-1 \right)$	$n_{T}n_{B},$

$(n_{I}-1)(n_{T}-1)$	$(n_{T}-1)(n_{B}-1)$

$I\wedge T\wedge B$

$n_{I}n_{T}n_{B},$ $(n_{I}-1)(n_{T}-1)(n_{B}-1)$

$E$	$n_{I}n_{T}{n_{B}n}_{R},$

$n_{I}n_{T}n_{B}(n_{R}-1)$

(i) Random structure	(ii) Fixed structure

**Figure A3: Multicentre randomised block factorial design.**

$U$	1	$U$	1

$I$	$n_{I}$	$C$	$n_{C}$	$B$	$n_{B}$

$I\wedge C$	$T$	${n_{C}n}_{T} I\wedge B n_{I}n_{B}$	$C\wedge B$

$n_{I}n_{C}$	$n_{C}n_{B}$	$I$	$n_{I}$

$I\wedge T$	$I\wedge C\wedge B T\wedge B$

$n_{I}n_{C}n_{T} n_{I}{n_{C}n}_{B}$	$n_{C}n_{T}n_{B}$

$I\wedge T\wedge B$

$n_{I}n_{C}n_{T}n_{B}$

$E$	$n_{I}{n_{C}n}_{T}n_{B}n_{R}$

(i) Random structure	(ii) Fixed structure

1 ***Hasse Diagrams***

See Bailey [42] for more on factors, finer and coarser relationships between factors, infima and suprema, notation, and Hasse diagrams. The technical detail is included here to highlight how we have adapted the approach in [42]. Specifically, we recommend that, where the treatment factors contribute fixed and random effects, it is helpful to give separate Hasse diagrams for fixed and random factors, rather than for unit and treatment structures. Figures A1, A2 and A3 above are the Hasse diagrams for our proposed designs. To clearly distinguish these Hasse diagrams from those in [42], we recommend using white diamonds to represent fixed factors and black diamonds for random factors, whether they are part of the unit or treatment structure. Note Bailey [42] defines two special factors, the Universal factor $U$ and the Equality factor $E$. The Universal factor is constant, making no distinctions between the units. It has a single level, for all of the units, and serves as the basis for estimating the overall mean. The Equality factor is different for each unit, so it has as many levels as there are units.

We will make frequent use of the concept of the infimum of two factors. Given two factors $F$ and $G$ on the same set, their ‘infimum’ is the factor denoted $F\wedge G$ each of whose levels is a combination of a level of $F$ with a level of $G$. The supremum $F\vee G$ of factors $F$ and $G$ is more complicated to define. If two units $\alpha$ and $\beta$ have the same level of $F$ or the same level of $G$ then they must have the same level of $F\vee G$. The supremum $F\vee G$ is the unique factor with the smallest number of levels that satisfies this criterion. It is apparent from Figure A1 above that the random factors are all nested and that the fixed factors are unstructured in our completely randomised factorial design.

In our randomised block factorial design, in addition to the infimum $I\wedge T$ of factors $I$ and $T$, we consider infima of the treatment and blocking factors, $I\wedge B$, $T\wedge B$ and $I\wedge T\wedge B$, because Batches constitute part of the complex intervention design, making interactions between treatment and blocking factors plausible. Factors $B$, $I\wedge B$, $T\wedge B$ and $I\wedge T\wedge B$ all contribute random effects. Next, we consider suprema of all pairs of random factors because the supremum of any two random factors must be included in the list of random factors used to identify the eigenspaces of the variance-covariance matrix $Cov\left( \boldsymbol{Y} \right)$ of patient outcomes. Unusually, this shows that the supremum of the random factors $I\wedge T$ and $I\wedge B$ is aliased with the fixed factor $I$. Yet, factor $I$ does not contribute a new variance parameter. We call its contribution a “dependent random effect”. As a Hasse diagram of the random structure should include all suprema of the random factors, we include factor $I$ in Figure A2(i) but give it a special symbol to distinguish it both from more traditional random factors and from the factor $I$ considered a fixed factor.

In our multicentre randomised block factorial design, in addition to $I\wedge T$, $I\wedge B$, $T\wedge B$ and $I\wedge T\wedge B$ we consider the infima $I\wedge C$, $C\wedge B$ and $I\wedge C\wedge B$, since Centre also constitutes part of the complex intervention design, making interactions plausible. Here, factors $C$, $I\wedge C$, $C\wedge B$ and $I\wedge C\wedge B$ all contribute random effects. Next, we consider suprema of all pairs of random factors and find that the supremum of the random factors $I\wedge C$ and $I\wedge B$ and the random factors $I\wedge B$ and $I\wedge T$ is aliased with the fixed factor $I$. This explains why we have included factor $I$ in Figure A3(i).

2 ***Analysis of Variance (ANOVA)***
  2.1 **Strata**

Denote by $V$ the $N$-dimensional vector space associated with the set of patients. If $F$ is a factor on the set of patients, denote by $V_{F}$ the vector space whose vectors are constant on each level of $F$. If $F$ is random, let $W_{F}$ be the subspace of $V_{F}$ which is orthogonal to all subspaces $V_{G}$ for random factors $G$ for which each level of $F$ is contained in a single level of $G$ but is not the whole of it. Under our orthogonality assumptions, $V$ is the orthogonal direct sum of these $W$ subspaces. Moreover, the number of degrees of freedom for each $W$ subspace is equal to its dimension.

Figures A1(i), A2(i) and A3(i) were used to obtain the eigenspaces of $Cov\left( \boldsymbol{Y} \right)$. These are the “strata” in Table 1 (first column). There is a stratum for each factor in Figures A1(i), A2(i) and A3(i). The strata are therefore precisely the $W$ subspaces found above ([42] gives more details on vector spaces and the null ANOVA).

Our completely randomised factorial design is relatively straightforward, since it has an orthogonal block structure in the sense defined by Houtman and Speed [56]. In contrast, the random structures in our randomised block and multicentre randomised block factorial designs are not orthogonal block structures under this [56] definition, because factor $I$ contributes a dependent random effect in each case. Specifically, for the randomised block factorial design, the intersection between the vector spaces $V_{I\wedge T}$and $V_{I\wedge B}$ is the vector space $V_{I}$. Also, $V_{I}$, $V_{I\wedge T}$and $V_{I\wedge B}$ are contained in the vector space $V_{I\wedge T\wedge B}$. As such, $V_{I\wedge T}$, $V_{I\wedge B}$ and $V_{I\wedge T\wedge B}$ are not orthogonal to one another. Similarly, for the multi-centre randomised block factorial design, the intersection between the vector spaces $V_{I\wedge C}$and $V_{I\wedge B}$ is the vector space $V_{I}$. Also, $V_{I\wedge C}$ is contained in $V_{I\wedge T}$, $V_{I\wedge B}$ is contained in $V_{I\wedge C\wedge B}$, and $V_{I\wedge C}$and $V_{I\wedge B}$are both contained in $V_{I\wedge T\wedge B}$. As such, here, $V_{I\wedge C}$, $V_{I\wedge B}$, $V_{I\wedge T}$, $V_{I\wedge C\wedge B}$ and $V_{I\wedge T\wedge B}$ are not orthogonal to one another (more detail on this can be found in Supplementary File B).

2.2 **Sources of variation**

Under reasonable orthogonality conditions, the collection of fixed factors gives a decomposition of the treatment space into orthogonal $W$ subspaces, just like that for the random factors. These subspaces are called *sources of variation*. Each source of variation is contained in a single stratum. The source of variation for fixed factor $F$ is contained in the stratum defined by the supremum of all random factors which occur below $F$ in the combined Hasse diagram (see Supplementary File C). In the completely randomised factorial design, the appropriate stratum is that associated with factor $I\wedge T$. That is, $W_{I\wedge T}$. This stratum is subdivided into a subspace $W_{I}$ associated with the fixed factor $I$ and a residual, the latter sometimes being referred to as the intervention-by-therapist interaction.

In our example, these sources of variation are obtained from Figures A1(ii), A2(ii) and A3(ii), and are shown in Table 1. If there is a stratum which is larger than the direct sum of these subspaces, then their orthogonal complement in that stratum is called Residual. In the randomised block factorial design, the relevant stratum for fixed factor $I$ is now the $W$-subspace $W_{I}$ associated with factor $I$. However, the fixed effect of $I$ takes up the whole of $W_{I}$, so no part of $W_{I}$ can give a residual mean square whose value can be used to provide an unbiased estimator of the appropriate residual. Instead, a linear combination of random factors is indicated. This is the case for the multicentre randomised block factorial design as well, except that the linear combination of random factors indicated differs.

2.3 **Degrees of Freedom**

The degrees of freedom are given in the third column in Table 1. The degrees of freedom associated with the strata are given on the right-hand side, and those relating to the fixed structure and residuals (where applicable) are given on the left-hand side. Any residual degrees of freedom are calculated by subtraction from the relevant stratum total.

2.4 **Expected Mean Squares**

The final column of Table 1 gives the expected mean squares. As $I$ is the only non-trivial factor with fixed effects, we assume there are unknown real numbers $\tau_{i}$ for each level $i$ of $I$ so the expectation of the random outcome variable $Y_{\omega}$ on patient $\omega$ is $\tau_{i}$ if factor $I$ takes level $i$ on patient $\omega$. Note that the vector $\boldsymbol{\tau}_{0}$ has the overall mean in every position, and the vector $\boldsymbol{\tau}_{I}+\boldsymbol{\tau}_{0}$ has the relevant treatment mean in every position. Then $\left\| \boldsymbol{\tau}_{0} \right\|^{2}$ and $\left\| \boldsymbol{\tau}_{I} \right\|^{2}$are the sums of squares of the entries in $\boldsymbol{\tau}_{0}$ and $\boldsymbol{\tau}_{I}$respectively. The $\xi$ are the eigenvalues of $Cov\left( \boldsymbol{Y} \right)$ (see Supplementary File D for details). These make it clear which residual is appropriate for testing the fixed effect of $I$ and what the appropriate F tests of the random effects are (for the latter, see Supplementary File E).

In a completely-randomised factorial design, the appropriate residual for testing the fixed effect of $I$ is the one whose expected mean square is $\xi_{I\wedge T}.$Importantly, it is not the residual whose expected mean square is $\xi_{E}$or $\left( {15\xi}_{I\wedge T}+{288\xi}_{E} \right)/303$, both of which are smaller than $\xi_{I\wedge T}$. If the therapist variance $\sigma_{T}^{2}=0$ then $\xi_{T}=\xi_{I\wedge T}$ and so ${MS}_{T}/R{MS}_{I\wedge T}$ has an F-distribution on 15 and 15 degrees of freedom in the running example, where ${MS}_{T}$ is the observed mean square for $T$ and ${RMS}_{I\wedge T}$ the observed residual mean square for $I\wedge T$. If the therapist variance in the intervention effect $\sigma_{I\wedge T}^{2}=0$, then $\xi_{I\wedge T}=\xi_{E}$ and so $R{MS}_{I\wedge T}/{MS}_{E}$ has an F-distribution on 15 and 288 degrees of freedom in the running example, where ${MS}_{E}$ is the observed mean square for $E$.

In a randomised block factorial design, the appropriate residual for testing the fixed effect of $I$ is one whose expected mean square is a linear combination of eigenvalues of $Cov\left( \boldsymbol{Y} \right)$, $\xi_{I\wedge T}+\xi_{I\wedge B}-\xi_{I\wedge T\wedge B}$. As such, the eigenvalues of $Cov\left( \boldsymbol{Y} \right)$ are linearly dependent. That is, the eigenvalue for $W_{I}$ is now a linear combination of those for subspaces $W_{I\wedge T},$ $W_{I\wedge B}$ and $W_{I\wedge T\wedge B}$. Although the random structure in Figure A2 is not an orthogonal block structure, the linear dependence on three other eigenvalues means that a linear combination of the mean squares for $W_{I\wedge T}$, $W_{I\wedge B}$ and $W_{I\wedge T\wedge B}$ from the ANOVA can be used to provide an unbiased estimator of the appropriate residual mean square. Satterthwaite [57] proposed that if a sample variance is a linear combination of independent mean squares, its exact distribution can be approximated by a chi-squaredistribution whose effective degrees of freedom are given by a specified formula involving the relevant mean squares and their degrees of freedom. The degrees of freedom for the linear combination of mean squares estimating the appropriate residual $\xi_{I\wedge T}+\xi_{I\wedge B}-\xi_{I\wedge T\wedge B}$ can be approximated by

${df}_{I}=\frac{\left[ {MS}_{I\wedge T} + {MS}_{I\wedge B} - {MS}_{I\wedge T\wedge B} \right]^{2}}{\frac{\left[ {MS}_{I\wedge T} \right]^{2}}{(n_{I}-1)(n_{T}-1)} + \frac{\left[ {MS}_{I\wedge B} \right]^{2}}{(n_{I}-1)(n_{B}-1)} + \frac{\left[ {MS}_{I\wedge T\wedge B} \right]^{2}}{(n_{I}-1)(n_{T}-1)(n_{B}-1)}}$	(A.1)

where ${MS}_{I\wedge T}$, ${MS}_{I\wedge B}$ and ${MS}_{I\wedge T\wedge B}$ are the observed mean squares for factors $I\wedge T$, $I\wedge B$ and $I\wedge T\wedge B$, respectively. This result can be used to provide an approximate F-test for the effect of $I$.

A similar situation arises in a multicentre randomised block factorial design, where the appropriate residual for testing the fixed effect of $I$ is one whose expected mean square is $\xi_{I\wedge C}+\xi_{I\wedge B}-\xi_{I\wedge C\wedge B}$. The eigenvalues of $Cov\left( \boldsymbol{Y} \right)$ are again linearly dependent, with the eigenvalue for $W_{I}$ a linear combination of those for subspaces $W_{I\wedge C},$ $W_{I\wedge B}$ and $W_{I\wedge C\wedge B}$. The degrees of freedom for the linear combination of mean squares estimating the appropriate residual $\xi_{I\wedge C}+\xi_{I\wedge B}-\xi_{I\wedge C\wedge B}$ can be approximated by

${df}_{I}=\frac{\left[ {MS}_{I\wedge C} + {MS}_{I\wedge B} - {MS}_{I\wedge C\wedge B} \right]^{2}}{\frac{\left[ {MS}_{I\wedge C} \right]^{2}}{(n_{I}-1)(n_{C}-1)} + \frac{\left[ {MS}_{I\wedge B} \right]^{2}}{(n_{I}-1)(n_{B}-1)} + \frac{\left[ {MS}_{I\wedge C\wedge B} \right]^{2}}{(n_{I}-1)(n_{C}-1)(n_{B}-1)}}$	(A.2)

where ${MS}_{I\wedge C}$, ${MS}_{I\wedge B}$ and ${MS}_{I\wedge C\wedge B}$ are the observed mean squares for factors $I\wedge C$, $I\wedge B$ and $I\wedge C\wedge B$. This can be used to provide an approximate F-test for the fixed effect of $I$.
