# Supplementary material for: Orthogonal Factorial Designs for Trials of Therapist‐Delivered Interventions: Randomizing Intervention‐Therapist Combinations to Patients
Source: Stat Med. 2026 Nov 10;45(25-27):e70813. doi: 10.1002/sim.70813 (PMC13749633; doi:10.1002/sim.70813)
Supplement: Supplementary file 5 [file SIM-45-0-s005.docx]

**Supplementary File B – Strata and the Importance of the Supremum**

Let us first consider the general situation. Let $F$and $G$ be two random factors on the set of units $\Omega$, with $n_{F}$ and $n_{G}$ levels respectively. Factor $E$ is the Equality factor, which is different for each unit, having as many levels as there are units. Following Bailey [42], $V$ is the $N$-dimensional vector space associated with $\Omega$ and we define vector subspaces $V_{F}$ and $V_{G}$ for each random factor. $V_{F}$ consists of all vectors which are constant on levels of factor $F$, $V_{G}$ consists of all vectors that are constant on levels of factor $G$, and $V$ consists of all vectors that are constant on levels of $E$. Figure B1 gives a diagrammatic representation of the vector spaces associated with these random factors.

**Figure B1: Vector subspaces defined by two random factors on the set of patients**

$\left( V_{F}+V_{G} \right)^{\perp}$	$V$

$V_{F}\cap V_{F\vee G}^{\perp}$	$V_{F\vee G}$	$V_{G}\cap V_{F\vee G}^{\perp}$

$V_{F} V_{G}$

Factors $F$and $G$ have a supremum $F\vee G$. The intersection of the subspaces $V_{F}$ and $V_{G}$ of $V$ is equal to the subspace of the supremum. That is, $V_{F}\cap V_{G}=V_{F\vee G}$ (see Proposition 10.2 in [42]). Bailey [42] defines factors $F$and $G$ as orthogonal to each other if the subspace ${W_{F}=V}_{F}\cap V_{F\vee G}^{\perp}$ is orthogonal to the subspace ${W_{G}=V}_{G}\cap V_{F\vee G}^{\perp}$ (p.179). Let ${W_{E}=\left( V_{F}+V_{G} \right)}^{\perp}$. Here, $V$ is the orthogonal direct sum of $W_{F}$, $W_{G},V_{F\vee G}$ and $W_{E}$ (i.e., $V=W_{F}\bigoplus W_{G}\bigoplus V_{F\vee G}\bigoplus W_{E}).$ This means that given any vector $\boldsymbol{v}$ $\boldsymbol{\in}$ $V$, there are unique vectors$\boldsymbol{v}_{F}\boldsymbol{\in}W_{F},$ $\boldsymbol{v}_{G}\boldsymbol{\in}W_{G}$, $\boldsymbol{x}$ $\boldsymbol{\in}V_{F\vee G}$ and $\boldsymbol{w}_{E}$ $\boldsymbol{\in}$ $W_{E}$ such that $\boldsymbol{v=}\boldsymbol{v}_{F}\boldsymbol{+}\boldsymbol{v}_{G}\boldsymbol{+x+}\boldsymbol{w}_{E}$ (see Theorems 2.4 and 10.3 in [42]). Thus, $\boldsymbol{v}_{F}$ can be considered a linear transformation of $\boldsymbol{v}$ onto $W_{F}$, and is referred to as the orthogonal projection of $\boldsymbol{v}$ onto $W_{F}$, or $\boldsymbol{P}_{W_{F}}\boldsymbol{v}$, where $\boldsymbol{P}_{W_{F}}$ is a projection matrix. Likewise, $\boldsymbol{v}_{G}$ is the orthogonal projection of $\boldsymbol{v}$ onto $W_{G}$ (i.e., $\boldsymbol{P}_{W_{G}}\boldsymbol{v}$), $\boldsymbol{x}$ is the orthogonal projection of $\boldsymbol{v}$ onto $V_{F\vee G}$ (i.e., $\boldsymbol{P}_{V_{F\vee G}}\boldsymbol{v}$) and $\boldsymbol{w}_{E}$ is the orthogonal projection of $\boldsymbol{v}$ onto $W_{E}$ (i.e., $\boldsymbol{P}_{W_{E}}\boldsymbol{v}$). Bailey [42] shows that when factors $F$and $G$ are orthogonal, their projection matrices commute. That is, $\boldsymbol{P}_{V_{F}}\boldsymbol{P}_{V_{G}}=\boldsymbol{P}_{V_{G}}\boldsymbol{P}_{V_{F}}=\boldsymbol{P}_{V_{F\vee G}}$ (see Theorem 10.3 in [42]).

Now for factor $F$on $\Omega$, we define $\boldsymbol{J}_{F}$as an $N\times N$ matrix whose $\left( \alpha,\beta\right)$ entry for any two units, $\alpha$ and $\beta$, is equal to

$\left\{ \begin{aligned} 1 \mathrm{if} F\left( \alpha\right)=F\left( \beta\right) \\ 0 \mathrm{otherwise} \end{aligned} \right.$ .

Likewise, for factor $G$on $\Omega$, we define $\boldsymbol{J}_{G}$as an $N\times N$ matrix whose $\left( \alpha,\beta\right)$ entry for any two units, $\alpha$ and $\beta$, is equal to

$\left\{ \begin{aligned} 1 \mathrm{if} G\left( \alpha\right)=G\left( \beta\right) \\ 0 \mathrm{otherwise} \end{aligned} \right.$ .

The levels of orthogonal factors $F$ and $G$ contribute random effects, respectively with variances $\sigma_{F}^{2}$ and $\sigma_{G}^{2}$. $\boldsymbol{Y}$ is a random vector of continuous outcomes, so the variance-covariance matrix of **Y** is given by

$Cov\left( \boldsymbol{Y} \right)=\sigma_{F}^{2}\boldsymbol{J}_{F}+\sigma_{G}^{2}\boldsymbol{J}_{G}+\sigma^{2}\boldsymbol{I}$	(B.1)

where $\sigma^{2}$ is the unit-level variance and $\boldsymbol{I}$ is the $N\times N$ identity matrix. Here, $\sigma_{F}^{2}\boldsymbol{J}_{F}+\sigma_{G}^{2}\boldsymbol{J}_{G}+\sigma^{2}\boldsymbol{I}$ can be rewritten as ${n_{F}\sigma}_{F}^{2}\boldsymbol{P}_{V_{F}}+{n_{G}\sigma}_{G}^{2}\boldsymbol{P}_{V_{G}}+\sigma^{2}\boldsymbol{P}_{V}$. Since $V_{F}=W_{F}\bigoplus V_{F\vee G},$ $V_{G}=W_{G}\bigoplus V_{F\vee G}$ and $V=W_{F}\bigoplus W_{G}\bigoplus V_{F\vee G}\bigoplus W_{E},$ $\boldsymbol{P}_{V_{F}}$ is equal to $\boldsymbol{P}_{W_{F}}+\boldsymbol{P}_{V_{F\vee G}},$ $\boldsymbol{P}_{V_{G}}$ is equal to $\boldsymbol{P}_{W_{G}}+\boldsymbol{P}_{V_{F\vee G}}$ and $\boldsymbol{P}_{V}$ is equal to $\boldsymbol{P}_{W_{F}}+\boldsymbol{P}_{W_{G}}+\boldsymbol{P}_{V_{F\vee G}}+\boldsymbol{P}_{W_{E}}$. Therefore, the variance-covariance matrix of **Y** can be re-written as

$Cov\left( \boldsymbol{Y} \right)={n_{F}\sigma}_{F}^{2}\left( \boldsymbol{P}_{W_{F}}+\boldsymbol{P}_{V_{F\vee G}} \right)+{n_{G}\sigma}_{G}^{2}\left( \boldsymbol{P}_{W_{G}}+\boldsymbol{P}_{V_{F\vee G}} \right)+\sigma^{2}\left( \boldsymbol{P}_{W_{F}}+\boldsymbol{P}_{W_{G}}+\boldsymbol{P}_{V_{F\vee G}}+\boldsymbol{P}_{W_{E}} \right)$. (B.2)

It is clear from this that the subspace for the supremum $V_{F\vee G}$is crucial when writing out the variance-covariance matrix in terms of mutually orthogonal projection matrices.

Equation (B.2) can then be re-written in terms of eigenvalues $\xi$ as

$$Cov\left( \boldsymbol{Y} \right)=\left( {n_{F}\sigma}_{F}^{2}+\sigma^{2} \right)\boldsymbol{P}_{W_{F}}+ {\left( {n_{G}\sigma}_{G}^{2}+\sigma^{2} \right)\boldsymbol{P}}_{W_{G}}+\left( {n_{F}\sigma}_{F}^{2}{{+n}_{G}\sigma}_{G}^{2}+\sigma^{2} \right)\boldsymbol{P}_{V_{F\vee G}}+{\sigma^{2}\boldsymbol{P}}_{W_{E}}$$

$=\xi_{F}\boldsymbol{P}_{W_{F}}+\xi_{G}\boldsymbol{P}_{W_{G}}+\xi_{F\vee G}\boldsymbol{P}_{V_{F\vee G}}+\xi_{E}\boldsymbol{P}_{W_{E}}$. (B.3)

This shows that the matrix $Cov\left( \boldsymbol{Y} \right)$ has *four* eigenspaces $W_{F}, W_{G},$ $V_{F\vee G}$ and $W_{E}$. Importantly, there is an eigenspace for each random factor but also one for the supremum. If the supremum contributes a dependent random effect, the eigenvalue $\xi_{F\vee G}$ is a linear combination of the eigenvalues associated with the random factors $F$, $G$ and $E$. Here, $\xi_{F\vee G}=\xi_{F}+\xi_{G}-\xi_{E}.$

Now let’s consider the more complex case with three random factors, $F,$ $G$ and their infimum $F\wedge G$ on $\Omega$, where $F\wedge G$ is not equal to $E$ (Figure A2). Here, $F,$ $G$ and $F\wedge G$ have $n_{F}$, $n_{G}$ and $n_{F}n_{G}$levels, respectively. Here,

$Cov\left( \boldsymbol{Y} \right)=\sigma_{F}^{2}\boldsymbol{J}_{F}+\sigma_{G}^{2}\boldsymbol{J}_{G}+\sigma_{F\wedge G}^{2}\boldsymbol{J}_{F\wedge G}+\sigma^{2}\boldsymbol{I}$	(B.4)

which, when rewritten in terms of projection matrices, gives

$$Cov\left( \boldsymbol{Y} \right)={n_{F}\sigma}_{F}^{2}\left( \boldsymbol{P}_{W_{F}}+\boldsymbol{P}_{V_{F\vee G}} \right)+{n_{G}\sigma}_{G}^{2}\left( \boldsymbol{P}_{W_{G}}+\boldsymbol{P}_{V_{F\vee G}} \right)+{n_{F}n_{G}\sigma}_{F\wedge G}^{2}\left( \boldsymbol{P}_{W_{F}}+\boldsymbol{P}_{W_{G}}+\boldsymbol{P}_{V_{F\vee G}}+\boldsymbol{P}_{W_{F\wedge G}} \right)+\sigma^{2}\left( \boldsymbol{P}_{W_{F}}+\boldsymbol{P}_{W_{G}}+\boldsymbol{P}_{V_{F\vee G}}+\boldsymbol{P}_{W_{F\wedge G}}+\boldsymbol{P}_{W_{E}} \right)$$

$$=\left( {n_{F}\sigma}_{F}^{2}+{n_{F}n_{G}\sigma}_{F\wedge G}^{2}+\sigma^{2} \right)\boldsymbol{P}_{W_{F}}+ {\left( {n_{G}\sigma}_{G}^{2}+{n_{F}n_{G}\sigma}_{F\wedge G}^{2}+\sigma^{2} \right)\boldsymbol{P}}_{W_{G}}+\left( {n_{F}\sigma}_{F}^{2}{{+n}_{G}\sigma}_{G}^{2}+{n_{F}n_{G}\sigma}_{F\wedge G}^{2}+\sigma^{2} \right)\boldsymbol{P}_{V_{F\vee G}}+\left( {n_{F}n_{G}\sigma}_{F\wedge G}^{2}+\sigma^{2} \right)\boldsymbol{P}_{W_{F\wedge G}}+{\sigma^{2}\boldsymbol{P}}_{W_{E}}$$

$=\xi_{F}\boldsymbol{P}_{W_{F}}+\xi_{G}\boldsymbol{P}_{W_{G}}+\xi_{F\vee G}\boldsymbol{P}_{V_{F\vee G}}+\xi_{F\wedge G}\boldsymbol{P}_{W_{F\wedge G}}+\xi_{V_{F\wedge G}^{\perp}}\boldsymbol{P}_{W_{E}}$. (B.5)

Again, the resulting matrix has an eigenspace for each factor and another for the supremum. If as before, the supremum contributes a dependent random effect, $\xi_{F\vee G}$ is a linear combination of the eigenvalues associated with random factors $F$, $G$ and $F\wedge G$. Here, $\xi_{F\vee G}=\xi_{F}+\xi_{G}-\xi_{F\wedge G}.$

**Figure B2: Vector subspaces defined by three random factors on the set of patients**

$V_{F\wedge G}^{\perp}$

$V_{F\wedge G}$	$V$

$V_{F}\cap V_{F\vee G}^{\perp}$	$V_{F\vee G}$	$V_{G}\cap V_{F\vee G}^{\perp}$

$V_{F} V_{G}$

$V_{F\wedge G}\cap\left( V_{F}+V_{G} \right)^{\perp}$

This has two general consequences. The first is that, to find the eigenspaces of $Cov\left( \boldsymbol{Y} \right)$, we need to include the supremum of any pair of factors which have random effects. The second consequence is that where the supremum contributes dependent random effects, we do not have an orthogonal block structure in the sense defined by Houtman and Speed [56] because the eigenvalue for the subspace associated with the supremum is a linear combination of those for the subspaces associated with other random factors.

**APPENDIX**

1. **Completely Randomised Factorial Design**

Figure B3 below gives a diagrammatic representation of the vector spaces associated with the random factors in our completely randomised factorial design.

**Figure B3: Vector subspaces associated with the random factors in a completely randomised factorial design**

$W_{E}$	$V$

$W_{I\wedge T}$	$W_{0}$	$W_{T}$

$V_{I\wedge T}$	$V_{T}$

Here, $V_{0}$ consists of all constant vectors, $V_{T}$ consists of all vectors constant on levels of $T$, and $V_{I\wedge T}$ consists of all vectors constant on levels of $I\wedge T$. The vector space $V$ can be expressed as the orthogonal direct sum of a series of $W$ subspaces (the “strata”). The first stratum, the “mean”, is the subspace of $V$ associated with the Universal factor $U$. This subspace is $W_{0}$, which here is equal to $V_{0}$. As $U$ has a single level, $W_{0}$ consists of all constant vectors. The second stratum, “therapists”, is the subspace $W_{T}$, which consists of all vectors in the subspace $V_{T}$ that are orthogonal to $W_{0}$ (i.e., $W_{T}=V_{T}\cap W_{0}^{\perp}$). The third stratum, “treatments”, is the subspace $W_{I\wedge T}$, consisting of all vectors in the subspace $V_{I\wedge T}$ orthogonal to $V_{T}$ ($W_{I\wedge T}=V_{I\wedge T}\cap V_{T}^{\perp}$). And finally, the fourth stratum, “patients”, is the subspace $W_{E}$, which consists of all vectors in $V$ that are orthogonal to $V_{I\wedge T}$ ($W_{E}=V_{I\wedge T}^{\perp}$). Then, $V_{T}$is the orthogonal direct sum of $W_{0}$ and $W_{T}$ ($V_{T}=W_{0}\bigoplus W_{T})$, $V_{I\wedge T}$ is the orthogonal direct sum of $W_{0}$, $W_{T}$ and $W_{I\wedge T}$ ($V_{I\wedge T}=$ $W_{0}\bigoplus W_{T}\bigoplus W_{I\wedge T}$) and $V$ is the orthogonal direct sum of $W_{0}$, $W_{T},$ $W_{I\wedge T}$ and $W_{E}$ ($V=W_{0}\bigoplus W_{T}\bigoplus W_{I\wedge T}\bigoplus W_{E}).$ This design has an orthogonal block structure in the sense defined by Houtman and Speed [56].

2. **Randomised Block Factorial Design**

Figure B4 gives a diagrammatic representation of the vector spaces associated with the random factors in our randomised block factorial design. Here, $V_{0}=W_{0}$ consists of all constant vectors, $V_{I}$ of all vectors constant on levels of $I$, $V_{T}$ of all vectors constant on levels of $T$, $V_{B}$ of all vectors constant on levels of $B$, $V_{I\wedge T}$ of all vectors constant on levels of $I\wedge T$, $V_{I\wedge B}$ of all vectors constant on levels of $I\wedge B$, $V_{T\wedge B}$ of all vectors constant on levels of $T\wedge B$, $V_{I\wedge T\wedge B}$ of all vectors constant on levels of $I\wedge T\wedge B$ and $V$ of all vectors constant on levels of $E$. The subspace $W_{I}$consists of all vectors in the subspace $V_{I}$ orthogonal to $W_{0}$ ($W_{I}=V_{I}\cap W_{0}^{\perp}). W_{T} \mathrm{and}W_{B}$ consist of all vectors in the subspaces $V_{T}$ and $V_{B}$ respectively, that are orthogonal to $W_{0}$ ($W_{T}=V_{T}\cap W_{0}^{\perp},$ $W_{B}=V_{B}\cap W_{0}^{\perp}).$ Here (contrary to before), $W_{I\wedge T}$ consists of all vectors in $V_{I\wedge T}$ that are orthogonal to $V_{I}$ and $V_{T}$ ($W_{I\wedge T}=V_{I\wedge T}\cap\left( V_{I}+V_{T} \right)^{\perp}$), $W_{I\wedge B}$ of all vectors in $V_{I\wedge B}$ orthogonal to $V_{I}$ and $V_{B} \left( W_{I\wedge B}={V_{I\wedge B}\cap\left( V_{I}+V_{B} \right)}^{\perp} \right),$ $W_{T\wedge B}$of all vectors in $V_{T\wedge B}$ orthogonal to $V_{T}$ and $V_{B}$ ($W_{T\wedge B}={V_{T\wedge B}\cap\left( V_{T}+V_{B} \right)}^{\perp}$) and $W_{I\wedge T\wedge B}$ of all vectors in $V_{I\wedge T\wedge B}$ orthogonal to $V_{I\wedge T}, V_{I\wedge B}$ and $V_{T\wedge B}$ ($W_{I\wedge T\wedge B}={V_{I\wedge T\wedge B}\cap\left( V_{I\wedge T}+V_{I\wedge B}+V_{T\wedge B} \right)}^{\perp}$). Finally, $W_{E}$ consists of all vectors in $V$ that are orthogonal to $V_{I\wedge T\wedge B}$ ($W_{E}=V\cap V_{I\wedge T\wedge B}^{\perp}).$

**Figure B4: Vector subspaces associated with the random factors in a randomised block factorial design**

$W_{E}$

$W_{I\wedge B}$	$W_{I}$	$W_{I\wedge T}$

$V_{I\wedge B}$	$V_{I}$	$V_{I\wedge T}$

$W_{0}$	$V$

$V_{B}$	$V_{T}$

$W_{B}$	$W_{T}$

$V_{T\wedge B}$	$W_{T\wedge B}$

$V_{I\wedge T\wedge B}$

$$W_{I\wedge T\wedge B}$$

Importantly, $V_{I\wedge T}$ and $V_{I\wedge B}$ overlap not just with $W_{0}$ but also with $W_{I},$ the subspace of $V$ associated with factor $I$ (the intersection between $V_{I\wedge T}$ and $V_{I\wedge B}$ is $V_{I}$). Also, $V_{I\wedge T}$ and $V_{I\wedge B}$ are contained in $V_{I\wedge T\wedge B}.$As such, $V_{I\wedge T}$, $V_{I\wedge B}$ and $V_{I\wedge T\wedge B}$ are not orthogonal to one another. However,

$V=W_{0}\bigoplus W_{I}\bigoplus W_{T}\bigoplus W_{B}\bigoplus W_{I\wedge T}\bigoplus W_{I\wedge B}\bigoplus W_{T\wedge B}\bigoplus W_{I\wedge T\wedge B}\bigoplus W_{E},$ $V_{I}=W_{0}\bigoplus W_{I}, V_{T}=W_{0}\bigoplus W_{T}$, $V_{B}=W_{0}\bigoplus W_{B}$, $V_{I\wedge T}=W_{0}\bigoplus W_{I}\bigoplus W_{T}\bigoplus W_{I\wedge T}, V_{I\wedge B}=W_{0}\bigoplus W_{I}\bigoplus W_{B}\bigoplus W_{I\wedge B},$

$V_{T\wedge B}=W_{0}\bigoplus W_{T}\bigoplus W_{B}\bigoplus W_{T\wedge B}$, and

$V_{I\wedge T\wedge B}=W_{0}\bigoplus W_{I}\bigoplus W_{T}\bigoplus W_{B}\bigoplus W_{I\wedge T}\bigoplus W_{I\wedge B}\bigoplus W_{T\wedge B}\bigoplus W_{I\wedge T\wedge B}$. (B.6)

3. **Multicentre Randomised Block Factorial Design**

Figure B5 gives a diagrammatic representation of the vector spaces associated with the random factors in our multicentre randomised block factorial design. As before, $V_{0}=W_{0}$ consists of all constant vectors, and $V$ consists of all vectors that are constant on levels of $E$. Here, $V_{I}, {V_{C}, V}_{B}$ and $V_{T}$consist of all vectors constant on levels of $I$, $C$, $B$ and $T$, respectively, and $V_{I\wedge C},$ ${V_{I\wedge B}, V}_{C\wedge B}, V_{I\wedge T},$

$V_{I\wedge C\wedge B}, V_{T\wedge B},$and $V_{I\wedge T\wedge B}$ consist of all vectors that are constant on levels of $I\wedge C, I\wedge B, C\wedge B,$

$I\wedge T, I\wedge C\wedge B$, $T\wedge B$ and $I\wedge T\wedge B$, respectively.

Here,

$W_{I}=V_{I}\cap W_{0}^{\perp},$

$W_{C}=V_{C}\cap W_{0}^{\perp}$,

$W_{B}=V_{B}\cap W_{0}^{\perp}$,

$W_{I\wedge C}=$ $V_{I\wedge C}\cap\left( V_{I}+V_{C} \right)^{\perp}$,

$W_{T}=V_{T}\cap V_{C}^{\perp},$

$W_{I\wedge B}=V_{I\wedge B}\cap\left( V_{I}+V_{B} \right)^{\perp}$,

$W_{C\wedge B}=V_{C\wedge B}\cap\left( V_{C}+V_{B} \right)^{\perp}$,

$W_{I\wedge T}=V_{I\wedge T}\cap\left( V_{I\wedge C}+V_{T} \right)^{\perp}$,

$W_{I\wedge C\wedge B}={V_{I\wedge C\wedge B}\cap\left( V_{I\wedge C}+V_{I\wedge B}+V_{C\wedge B} \right)}^{\perp}$,

$W_{T\wedge B}={V_{T\wedge B}\cap\left( V_{T}+V_{C\wedge B} \right)}^{\perp}$,

$W_{I\wedge T\wedge B}={V_{I\wedge T\wedge B}\cap\left( V_{I\wedge T}+V_{I\wedge C\wedge B}+V_{T\wedge B} \right)}^{\perp}$, and finally

$W_{E}=V\cap V_{I\wedge T\wedge B}^{\perp}$.

**Figure B5: Vector subspaces associated with the random factors in a multicentre randomised block factorial design**

$W_{E}$

$W_{I}$

$W_{I\wedge B}$	$V_{I}$	$W_{I\wedge C}$

$W_{I\wedge C\wedge B}$	$W_{I\wedge T}$

$V_{I\wedge B}V_{I\wedge C}$

$V_{I\wedge C\wedge B}$	$W_{0}$	$V$

$V_{B}$	$V_{C}$	$V_{I\wedge T}$

$V_{C\wedge B}$

$W_{B}$	$W_{C}$

$W_{C\wedge B}$

$W_{I\wedge T\wedge B}$	$V_{T}$

$W_{T}$

$V_{I\wedge T\wedge B}$	$W_{T\wedge B}$	$V_{T\wedge B}$

Importantly, $V_{I\wedge C}$ and $V_{I\wedge B}$ overlap not just with $W_{0}$ but also with $W_{I},$ the subspace of $V$ associated with fixed factor $I$ (the intersection between $V_{I\wedge C}$ and $V_{I\wedge B}$ is $V_{I}$). $V_{I\wedge C}$ is contained in $V_{I\wedge T}$, $V_{I\wedge B}$ is contained in $V_{I\wedge C\wedge B}$, and $V_{I\wedge C}$ and $V_{I\wedge B}$ are both contained in $V_{I\wedge T\wedge B}$. As such, $V_{I\wedge C}, V_{I\wedge B}$, $V_{I\wedge T}, V_{I\wedge C\wedge B}$ and $V_{I\wedge T\wedge B}$ are not orthogonal to one another. Here,

$$V=W_{0}\bigoplus W_{I}\bigoplus W_{C}\bigoplus W_{B}\bigoplus W_{I\wedge C}\bigoplus{W_{T}\bigoplus W}_{I\wedge B}\bigoplus W_{C\wedge B}\bigoplus W_{I\wedge T}\bigoplus W_{I\wedge C\wedge B}\bigoplus W_{T\wedge B}\bigoplus W_{I\wedge T\wedge B}\bigoplus W_{E},$$

$V_{I}=W_{0}\bigoplus W_{I}, V_{C}=W_{0}\bigoplus W_{C}$, $V_{B}=W_{0}\bigoplus W_{B}, {V_{I\wedge C}=W_{0}\bigoplus W_{I}\bigoplus W_{C}\bigoplus W_{I\wedge C}, V}_{T}=W_{0}\bigoplus W_{C}\bigoplus W_{T}$, $V_{I\wedge B}=W_{0}\bigoplus W_{I}\bigoplus W_{B}\bigoplus W_{I\wedge B},$ $V_{C\wedge B}=W_{0}\bigoplus W_{C}\bigoplus W_{B}\bigoplus W_{C\wedge B}$,

$V_{I\wedge T}=W_{0}\bigoplus W_{I}\bigoplus W_{C}\bigoplus W_{I\wedge C}\bigoplus W_{T}\bigoplus W_{I\wedge T},$

$V_{I\wedge C\wedge B}=W_{0}\bigoplus W_{I}\bigoplus W_{C}\bigoplus W_{B}\bigoplus W_{I\wedge C}\bigoplus W_{I\wedge B}\bigoplus W_{C\wedge B}\bigoplus W_{I\wedge C\wedge B}$,

$V_{T\wedge B}=W_{0}\bigoplus W_{C}\bigoplus W_{B}\bigoplus W_{T}\bigoplus W_{C\wedge B}\bigoplus W_{T\wedge B},$ and

$V_{I\wedge T\wedge B}=W_{0}\bigoplus W_{I}\bigoplus W_{C}\bigoplus W_{B}\bigoplus W_{I\wedge C}\bigoplus W_{T}\bigoplus W_{I\wedge B}\bigoplus W_{C\wedge B}\bigoplus W_{I\wedge T}\bigoplus W_{I\wedge C\wedge B}\bigoplus W_{T\wedge B}\bigoplus W_{I\wedge T\wedge B}$. (B.7)
