# Supplementary material for: Orthogonal Factorial Designs for Trials of Therapist‐Delivered Interventions: Randomizing Intervention‐Therapist Combinations to Patients
Source: Stat Med. 2026 Nov 10;45(25-27):e70813. doi: 10.1002/sim.70813 (PMC13749633; doi:10.1002/sim.70813)
Supplement: Supplementary file 6 [file SIM-45-0-s006.docx]

**Supplementary File F – Relationships between Eigenvalues and Variance Components**

1. **Randomised Block Factorial Design**

It is possible to write the eigenvalues $\xi_{E}$, $\xi_{I\wedge T\wedge B}, \xi_{T\wedge B}, \xi_{I\wedge B}, \xi_{I\wedge T}$, $\xi_{B}$, $\xi_{T}, \xi_{I}$ and $\xi_{0}$ in (D.4) and the ANOVA table (Table 1b) in terms of $\sigma_{e}^{2}$, $\sigma_{u1}^{2}, \sigma_{u2}^{2}, \sigma_{u3}^{2}, \sigma_{v1}^{2}, \sigma_{v2}^{2}$ and $\sigma_{v3}^{2}$from the regression model in (8) as follows,

$\xi_{E} =$ $\sigma_{e}^{2}$	(F.1)

$\xi_{I\wedge T\wedge B} =\sigma_{v3}^{2}n_{R}+\sigma_{e}^{2}$	(F.2)

$\xi_{T\wedge B}$ $=\sigma_{u3}^{2}n_{I}n_{R}+\sigma_{v3}^{2}n_{R}+\sigma_{e}^{2}$	(F.3)

$\xi_{I\wedge B}$ $=\sigma_{v2}^{2}n_{T}n_{R}+\sigma_{v3}^{2}n_{R}+\sigma_{e}^{2}$	(F.4)

$\xi_{I\wedge T}$ $=\sigma_{v1}^{2}n_{B}n_{R}+\sigma_{v3}^{2}n_{R}+\sigma_{e}^{2}$	(F.5)

$\xi_{B} =\sigma_{u2}^{2}n_{I}n_{T}n_{R}+\sigma_{u3}^{2}n_{I}n_{R}+\sigma_{v2}^{2}n_{T}n_{R}+\sigma_{v3}^{2}n_{R}+\sigma_{e}^{2}$	(F.6)

$\xi_{T} =\sigma_{u1}^{2}n_{I}n_{B}n_{R}\boldsymbol{+}\sigma_{u3}^{2}n_{I}n_{R}\boldsymbol{+}\sigma_{v1}^{2}n_{B}n_{R}+\sigma_{v3}^{2}n_{R}+\sigma_{e}^{2}$	(F.7)

$\xi_{I} =\sigma_{v1}^{2}n_{B}n_{R}\boldsymbol{+}\sigma_{v2}^{2}n_{T}n_{R}+\sigma_{v3}^{2}n_{R}+\sigma_{e}^{2}$	(F.8)

$\xi_{0} ={0+\sigma}_{u1}^{2}n_{I}n_{B}n_{R}+\sigma_{u2}^{2}n_{I}n_{T}n_{R}\boldsymbol{+}\sigma_{u3}^{2}n_{I}n_{R}\boldsymbol{+}\sigma_{v1}^{2}n_{B}n_{R}\boldsymbol{+}\sigma_{v2}^{2}n_{T}n_{R}+\sigma_{v3}^{2}n_{R}+\sigma_{e}^{2}$	(F.9)

So, $\sigma_{e}^{2}=\sigma^{2}$, $\sigma_{u1}^{2}=\sigma_{T}^{2}, \sigma_{u2}^{2}=\sigma_{B}^{2}, \sigma_{u3}^{2}=\sigma_{T\wedge B}^{2}, \sigma_{v1}^{2}=\sigma_{I\wedge T}^{2}, \sigma_{v2}^{2}=\sigma_{I\wedge B}^{2}$, $\sigma_{v3}^{2}=\sigma_{I\wedge T\wedge B}^{2}$, and $\sigma_{0}^{2}=0$.

2. **Multicentre Randomised Block Factorial Design**

The eigenvalues $\xi_{E}$, $\xi_{I\wedge T\wedge B}, \xi_{T\wedge B},\xi_{I\wedge C\wedge B}, {\xi_{I\wedge T},\xi_{C\wedge B}, \xi}_{I\wedge B}, \xi_{T}, \xi_{I\wedge C}, {\xi_{B}, \xi}_{C}, \xi_{I}$ and $\xi_{0}$ in (D.6) and the ANOVA table (Table 1c) can be written in terms of $\sigma_{e}^{2}$, $\sigma_{u1}^{2}, \sigma_{u2}^{2}, \sigma_{u3}^{2}, {\sigma_{u4}^{2}, \sigma_{u5}^{2}, \sigma}_{v1}^{2}, \sigma_{v2}^{2}, \sigma_{v3}^{2}, \sigma_{v4}^{2}$ and $\sigma_{v5}^{2}$from the regression model in (9) as follows,

$\xi_{E} =$ $\sigma_{e}^{2}$	(F.10)

$\xi_{I\wedge T\wedge B} =\sigma_{v3}^{2}n_{R}+\sigma_{e}^{2}$	(F.11)

$\xi_{T\wedge B}$ $=\sigma_{u3}^{2}n_{I}n_{R}+\sigma_{v3}^{2}n_{R}+\sigma_{e}^{2}$	(F.12)

$\xi_{I\wedge C\wedge B} =\sigma_{v3}^{2}n_{R}+\sigma_{v5}^{2}n_{T}n_{R}+\sigma_{e}^{2}$	(F.13)

$\xi_{I\wedge T}$ $=\sigma_{v1}^{2}n_{B}n_{R}+\sigma_{v3}^{2}n_{R}+\sigma_{e}^{2}$	(F.14)

$\xi_{C\wedge B}=\sigma_{u3}^{2}n_{I}n_{R}+\sigma_{u5}^{2}n_{I}n_{T}n_{R}+\sigma_{v3}^{2}n_{R}+\sigma_{v5}^{2}n_{T}n_{R}+\sigma_{e}^{2}$	(F.15)

$\xi_{I\wedge B}$ $=\sigma_{v2}^{2}{n_{C}n}_{T}n_{R}+\sigma_{v3}^{2}n_{R}+\sigma_{v5}^{2}n_{T}n_{R}+\sigma_{e}^{2}$	(F.16)

$\xi_{T} =\sigma_{u1}^{2}n_{I}n_{B}n_{R}+\sigma_{u3}^{2}n_{I}n_{R}+\sigma_{v1}^{2}n_{B}n_{R}+\sigma_{v3}^{2}n_{R}+\sigma_{e}^{2}$	(F.17)

$\xi_{I\wedge C}=\sigma_{v1}^{2}n_{B}n_{R}+\sigma_{v3}^{2}n_{R}+\sigma_{v4}^{2}n_{B}n_{T}n_{R}+\sigma_{v5}^{2}n_{T}n_{R}+\sigma_{e}^{2}$	(F.18)

$\xi_{B} =\sigma_{u2}^{2}n_{I}{n_{C}n}_{T}n_{R}+\sigma_{u3}^{2}n_{I}n_{R}+\sigma_{u5}^{2}n_{I}n_{T}n_{R}+\sigma_{v2}^{2}{n_{C}n}_{T}n_{R}+\sigma_{v3}^{2}n_{R}+\sigma_{v5}^{2}n_{T}n_{R}+\sigma_{e}^{2}$	(F.19)

$$\xi_{C} =\sigma_{u1}^{2}n_{I}n_{B}n_{R}\boldsymbol{+}\sigma_{u3}^{2}n_{I}n_{R}\boldsymbol{+}\sigma_{u4}^{2}n_{I}n_{B}n_{T}n_{R}\boldsymbol{+}\sigma_{u5}^{2}n_{I}n_{T}n_{R}\boldsymbol{+}\sigma_{v1}^{2}n_{B}n_{R}\boldsymbol{+}\sigma_{v3}^{2}n_{R}\boldsymbol{+}\sigma_{v4}^{2}n_{B}n_{T}n_{R}\boldsymbol{+}$$

$\sigma_{v5}^{2}n_{T}n_{R}\boldsymbol{+}\sigma_{e}^{2}$	(F.20)

$\xi_{I} =\sigma_{v1}^{2}n_{B}n_{R}\boldsymbol{+}\sigma_{v2}^{2}{n_{C}n}_{T}n_{R}+\sigma_{v3}^{2}n_{R}\boldsymbol{+}\sigma_{v4}^{2}n_{B}n_{T}n_{R}\boldsymbol{+}\sigma_{v5}^{2}n_{T}n_{R}\boldsymbol{+}\sigma_{e}^{2}$	(F.21)

$$\xi_{0} ={{0+\sigma}_{u1}^{2}n_{I}n_{B}n_{R}+\sigma_{u2}^{2}n_{I}{n_{C}n}_{T}n_{R}+\sigma_{u3}^{2}n_{I}n_{R}+\sigma}_{u4}^{2}n_{I}n_{B}n_{T}n_{R}+\sigma_{u5}^{2}n_{I}n_{T}n_{R}+\sigma_{v1}^{2}n_{B}n_{R}+$$

$\sigma_{v2}^{2}{n_{C}n}_{T}n_{R}+\sigma_{v3}^{2}n_{R}+\sigma_{v4}^{2}n_{B}n_{T}n_{R}+\sigma_{v5}^{2}n_{T}n_{R}+\sigma_{e}^{2}$	(F.22)

As such, $\sigma_{e}^{2}=\sigma^{2}$, $\sigma_{u1}^{2}=\sigma_{T}^{2}, \sigma_{u2}^{2}=\sigma_{B}^{2}, \sigma_{u3}^{2}=\sigma_{T\wedge B}^{2}, {\sigma_{u4}^{2}=\sigma_{C}^{2}, \sigma_{u5}^{2}=\sigma_{C\wedge B}^{2}, \sigma}_{v1}^{2}=\sigma_{I\wedge T}^{2}, \sigma_{v2}^{2}=\sigma_{I\wedge B}^{2},$

$\sigma_{v3}^{2}=\sigma_{I\wedge T\wedge B}^{2}, \sigma_{v4}^{2}=\sigma_{I\wedge C}^{2}, \sigma_{v5}^{2}=\sigma_{I\wedge C\wedge B}^{2}$ and $\sigma_{0}^{2}=0.$
