# Supplementary material for: Orthogonal Factorial Designs for Trials of Therapist‐Delivered Interventions: Randomizing Intervention‐Therapist Combinations to Patients
Source: Stat Med. 2026 Nov 10;45(25-27):e70813. doi: 10.1002/sim.70813 (PMC13749633; doi:10.1002/sim.70813)
Supplement: Supplementary file 7 [file SIM-45-0-s007.docx]

**Supplementary File E – Appropriate F tests for the random effects being equal to zero**

1. **Completely Randomised Factorial Design**

It can be seen from Equation (D.2) and Table 1a) that if $\sigma_{T}^{2}=0$ then $\xi_{T}=\xi_{I\wedge T}$ and so ${MS}_{T}/R{MS}_{I\wedge T}$ has an F-distribution on 15 and 15 degrees of freedom, where ${MS}_{T}$ is the observed mean square for $T$ and ${RMS}_{I\wedge T}$is the observed residual mean square for factor $I\wedge T$. This provides an exact F test for whether the random effect of $T$ is zero. If $\sigma_{I\wedge T}^{2}=0$ then $\xi_{I\wedge T}=\xi_{E}$ and so ${RMS}_{I\wedge T}/{MS}_{E}$ has an F-distribution on 15 and 288 degrees of freedom, which provides an exact F test for whether the random effect of $I\wedge T$ is zero where ${MS}_{E}$ is the observed mean square for $E$. These are summarised below in Table E1.

**Table E1: F-tests for the random effects in the**

**completely randomised factorial design**

| **Random Effect** | **F Statistic** | **Degrees of Freedom** | |
| --- | --- | --- | --- |
|  |  | **Numerator** | **Denominator** |
| $\sigma_{T}^{2}$ | ${MS}_{T}/R{MS}_{I\wedge T}$ | 15 | 15 |
| $\sigma_{I\wedge T}^{2}$ | ${RMS}_{I\wedge T}/{MS}_{E}$ | 15 | 288 |

2. **Randomised Block Factorial Design**

The corresponding F tests for the random effects in the randomised block factorial design are taken from Equation (D.4) and Table 1b) and summarised below in Table E2. Note that the F tests are exact for testing whether $\sigma_{I\wedge T}^{2}=0$, $\sigma_{I\wedge B}^{2}=0$, $\sigma_{T\wedge B}^{2}=0$ and $\sigma_{I\wedge T\wedge B}^{2}=0$. However, they are only approximate for testing whether $\sigma_{T}^{2}=0$ or $\sigma_{B}^{2}=0$.

**Table E2: F-tests for the random effects in the randomised block factorial design**

| **Random Effect** | **F Statistic** | **Degrees of Freedom** | |
| --- | --- | --- | --- |
|  |  | **Numerator** | **Denominator** |
| $\sigma_{T}^{2}$ | ${MS}_{T}/{(MS}_{I\wedge T}+{MS}_{T\wedge B}-{MS}_{I\wedge T\wedge B})$ | 15 | ${df}_{T}$ |
| $\sigma_{B}^{2}$ | ${MS}_{B}/{(MS}_{I\wedge B}+{MS}_{T\wedge B}-{MS}_{I\wedge T\wedge B})$ | 4 | ${df}_{B}$ |
| $\sigma_{I\wedge T}^{2}$ | ${MS}_{I\wedge T}/{MS}_{I\wedge T\wedge B}$ | 15 | 60 |
| $\sigma_{I\wedge B}^{2}$ | ${MS}_{I\wedge B}/{MS}_{I\wedge T\wedge B}$ | 4 | 60 |
| $\sigma_{T\wedge B}^{2}$ | ${MS}_{T\wedge B}/{MS}_{I\wedge T\wedge B}$ | 60 | 60 |
| $\sigma_{I\wedge T\wedge B}^{2}$ | ${MS}_{I\wedge T\wedge B}/{MS}_{E}$ | 60 | 160 |

Note: $MS$ are observed mean squares associated with $T$, $B$, $I\wedge T$, $I\wedge B$, $T\wedge B$, $I\wedge T\wedge B$ or$E$.

In this table,

${df}_{T}=\frac{\left[ {MS}_{I\wedge T} + {MS}_{T\wedge B} -{MS}_{I\wedge T\wedge B} \right]^{2}}{\frac{\left[ {MS}_{I\wedge T} \right]^{2}}{(n_{I}-1)(n_{T}-1)} + \frac{\left[ {MS}_{T\wedge B} \right]^{2}}{(n_{T}-1)(n_{B}-1)} + \frac{\left[ {MS}_{I\wedge T\wedge B} \right]^{2}}{(n_{I}-1)(n_{T}-1)(n_{B}-1)}}$	(E.1)

and

${df}_{B}=\frac{\left[ {MS}_{I\wedge B} + {MS}_{T\wedge B}- {MS}_{I\wedge T\wedge B} \right]^{2}}{\frac{\left[ {MS}_{I\wedge B} \right]^{2}}{(n_{I}-1)(n_{B}-1)} + \frac{\left[ {MS}_{T\wedge B} \right]^{2}}{(n_{T}-1)(n_{B}-1)} + \frac{\left[ {MS}_{I\wedge T\wedge B} \right]^{2}}{(n_{I}-1)(n_{T}-1)(n_{B}-1)}}$.	(E.2)

4. **Multicentre Randomised Block Factorial Design**

The corresponding F tests for the random effects in the randomised block factorial design are taken from Equation (D.6) and Table 1c) and summarised below in Table E3. Note that the F tests are exact for testing whether $\sigma_{I\wedge B}^{2}=0$, $\sigma_{I\wedge T}^{2}=0$, $\sigma_{I\wedge C\wedge B}^{2}=0$, $\sigma_{T\wedge B}^{2}=0$ and $\sigma_{I\wedge T\wedge B}^{2}=0$. They are only approximate for testing whether $\sigma_{C}^{2}=0,$ $\sigma_{B}^{2}=0, \sigma_{I\wedge C}^{2}=0,$ $\sigma_{T}^{2}=0$ or $\sigma_{C\wedge B}^{2}=0$.

**Table E3: F-tests for the random effects in the multicentre randomised block factorial design**

| **Random Effect** | **F Statistic** | **Degrees of Freedom** | |
| --- | --- | --- | --- |
|  |  | **Numerator** | **Denominator** |
| $\sigma_{C}^{2}$ | ${MS}_{C}/{(MS}_{T}+{MS}_{I\wedge C}+{MS}_{C\wedge B}+{MS}_{I\wedge T\wedge B}-{MS}_{I\wedge T}-{MS}_{T\wedge B}{-MS}_{I\wedge C\wedge B})$ | 5 | ${df}_{C}$ |
| $\sigma_{B}^{2}$ | ${MS}_{B}/{(MS}_{I\wedge B}+{MS}_{C\wedge B}-{MS}_{I\wedge C\wedge B})$ | 4 | ${df}_{B}$ |
| $\sigma_{I\wedge C}^{2}$ | ${MS}_{I\wedge C}/{(MS}_{I\wedge T}+{MS}_{I\wedge C\wedge B}-{MS}_{I\wedge T\wedge B})$ | 5 | ${df}_{I\wedge C}$ |
| $\sigma_{T}^{2}$ | ${MS}_{T}/{(MS}_{I\wedge T}+{MS}_{T\wedge B}-{MS}_{I\wedge T\wedge B})$ | 42 | ${df}_{T}$ |
| $\sigma_{I\wedge B}^{2}$ | ${MS}_{I\wedge B}/{MS}_{I\wedge C\wedge B}$ | 4 | 20 |
| $\sigma_{C\wedge B}^{2}$ | ${MS}_{C\wedge B}/{(MS}_{T\wedge B}+{MS}_{I\wedge C\wedge B}-{MS}_{I\wedge T\wedge B})$ | 20 | ${df}_{C\wedge B}$ |
| $\sigma_{I\wedge T}^{2}$ | ${MS}_{I\wedge T}/{MS}_{I\wedge T\wedge B}$ | 42 | 168 |
| $\sigma_{I\wedge C\wedge B}^{2}$ | ${MS}_{I\wedge C\wedge B}/{MS}_{I\wedge T\wedge B}$ | 20 | 168 |
| $\sigma_{T\wedge B}^{2}$ | ${MS}_{T\wedge B}/{MS}_{I\wedge T\wedge B}$ | 168 | 168 |
| $\sigma_{I\wedge T\wedge B}^{2}$ | ${MS}_{I\wedge T\wedge B}/{MS}_{E}$ | 168 | 480 |

Note: $MS$ are observed mean squares associated with $C$, $B$, $I\wedge C$, $T$, $I\wedge B$, $C\wedge B$, $I\wedge T$, $I\wedge C\wedge B$, $T\wedge B$, $I\wedge T\wedge B$or$E$.

In this table,

${df}_{C}=\frac{\left[ {MS}_{T} + {MS}_{I\wedge C} + {MS}_{C\wedge B} + {MS}_{I\wedge T\wedge B} - {MS}_{I\wedge T} - {MS}_{T\wedge B} {- MS}_{I\wedge C\wedge B} \right]^{2}}{\frac{\left[ {MS}_{T} \right]^{2}}{n_{C}\left( n_{T}-1 \right)} + \frac{\left[ {MS}_{I\wedge C} \right]^{2}}{\left( n_{I}-1 \right)\left( n_{C}-1 \right)} + \frac{\left[ {MS}_{C\wedge B} \right]^{2}}{\left( n_{C}-1 \right)\left( n_{B}-1 \right)} + \frac{\left[ {MS}_{I\wedge T\wedge B} \right]^{2}}{\left( n_{I}-1 \right)n_{C}\left( n_{T}-1 \right)\left( n_{B}-1 \right)} + \frac{\left[ {MS}_{I\wedge T} \right]^{2}}{\left( n_{I}-1 \right)n_{C}\left( n_{T}-1 \right)} + \frac{\left[ {MS}_{T\wedge B} \right]^{2}}{n_{C}\left( n_{T}-1 \right)\left( n_{B}-1 \right)} + \frac{\left[ {MS}_{I\wedge C\wedge B} \right]^{2}}{\left( n_{I}-1 \right)\left( n_{C}-1 \right)\left( n_{B}-1 \right)}}$, (E.3)

${df}_{B}=\frac{\left[ {MS}_{I\wedge B} +{MS}_{C\wedge B} -{MS}_{I\wedge C\wedge B} \right]^{2}}{\frac{\left[ {MS}_{I\wedge B} \right]^{2}}{(n_{I}-1)(n_{B}-1)} + \frac{\left[ {MS}_{C\wedge B} \right]^{2}}{(n_{C}-1)(n_{B}-1)} + \frac{\left[ {MS}_{I\wedge C\wedge B} \right]^{2}}{(n_{I}-1)(n_{C}-1)(n_{B}-1)}}$ ,	(E.4)

${df}_{I\wedge C}=\frac{\left[ {MS}_{I\wedge T} +{MS}_{I\wedge C\wedge B} - {MS}_{I\wedge T\wedge B} \right]^{2}}{\frac{\left[ {MS}_{I\wedge T} \right]^{2}}{(n_{I}-1)n_{C}(n_{T}-1)} + \frac{\left[ {MS}_{I\wedge C\wedge B} \right]^{2}}{(n_{I}-1)(n_{C}-1)(n_{B}-1)} + \frac{\left[ {MS}_{I\wedge T\wedge B} \right]^{2}}{(n_{I}-1)n_{C}(n_{T}-1)(n_{B}-1)}}$ ,	(E.5)

${df}_{T}=\frac{\left[ {MS}_{I\wedge T} + {MS}_{T\wedge B}- {MS}_{I\wedge T\wedge B} \right]^{2}}{\frac{\left[ {MS}_{I\wedge T} \right]^{2}}{(n_{I}-1)n_{C}(n_{T}-1)} + \frac{\left[ {MS}_{T\wedge B} \right]^{2}}{n_{C}(n_{T}-1)(n_{B}-1)} + \frac{\left[ {MS}_{I\wedge T\wedge B} \right]^{2}}{(n_{I}-1)n_{C}(n_{T}-1)(n_{B}-1)}}$ ,	(E.6)

and

${df}_{C\wedge B}=\frac{\left[ {MS}_{T\wedge B} +{MS}_{I\wedge C\wedge B} - {MS}_{I\wedge T\wedge B} \right]^{2}}{\frac{\left[ {MS}_{T\wedge B} \right]^{2}}{n_{C}(n_{T}-1)(n_{B}-1)} + \frac{\left[ {MS}_{I\wedge C\wedge B} \right]^{2}}{(n_{I}-1)(n_{C}-1)(n_{B}-1)} + \frac{\left[ {MS}_{I\wedge T\wedge B} \right]^{2}}{(n_{I}-1)n_{C}(n_{T}-1)(n_{B}-1)}}$ .	(E.7)
