# Supplementary material for: Orthogonal Factorial Designs for Trials of Therapist‐Delivered Interventions: Randomizing Intervention‐Therapist Combinations to Patients
Source: Stat Med. 2026 Nov 10;45(25-27):e70813. doi: 10.1002/sim.70813 (PMC13749633; doi:10.1002/sim.70813)
Supplement: Supplementary file 8 [file SIM-45-0-s008.html]

Supplementary File G - Example R Output


# Supplementary File G - Example R Output

Author

Rebecca Walwyn

A single dataset for each case below was generated for illustrative purposes only.

## **1. Completely Randomised Factorial Design**

### a) Case One - Two Interventions Only

```
CrossedDataset1 <- readRDS("P:/CTRU/Projects/Methodology/2_Statistical Methodology/2022_Rebecca Walwyn_NIHR Advanced Fellowship/7_Papers/2A_Crossed Therapist Designs/4_Submitted_20250930/3_Uploaded to GitHub/CrossedDataset1.rds")

# ANOVA

aov1 <- aov(y~I+Error(T+I:T), contrasts=list(I=contr.sum), data=CrossedDataset1)
summary(aov1)
```

```
Error: T
          Df Sum Sq Mean Sq F value Pr(>F)
Residuals 15  232.9   15.53               

Error: T:I
          Df Sum Sq Mean Sq F value   Pr(>F)    
I          1 127.31  127.31   21.07 0.000354 ***
Residuals 15  90.63    6.04                     
---
Signif. codes:  0 '***' 0.001 '**' 0.01 '*' 0.05 '.' 0.1 ' ' 1

Error: Within
           Df Sum Sq Mean Sq F value Pr(>F)
Residuals 288  919.6   3.193
```

```
coefficients(aov1)
```

```
(Intercept) :
(Intercept) 
    6.50708 

T :
numeric(0)

T:I :
       I1 
0.6307375 

Within :
numeric(0)
```

```
print(model.tables(aov1,"means"))
```

```
Tables of means
Grand mean
        
6.50708 

 I 
I
    1     2 
7.138 5.876
```

```
# Note that the fixed effect of factor I appears in the W_I∧T stratum, where the 
# residual degrees of freedom are 15. Note also, for the fixed effect of factor I, 
# F = 21.07 and p = 0.000354.  It can be  seen that the estimates of ξ_T, ξ_I∧T and 
# ξ_E are 15.53, 6.04 and 3.193 respectively and that δ_0= 6.50708 and δ_1= 
# 0.6307375. Finally note that 0.6307375 is half the difference between the mean of
# all outcomes on level 1 of factor I (i.e. 7.138) and the mean of all outcomes on 
# level 2 of factor I (i.e. 5.876).

# For the random effect of factor T, F = 15.53/6.04 = 2.57119205298 on 15 and 15 
# degrees of freedom

pf(2.57119205298, 15, 15, lower.tail=F)
```

```
[1] 0.03859187
```

```
# For the random effect of factor I∧T, F = 6.04/3.193 = 1.89163795803 on 15 and 288
# degrees of freedom

pf(1.89163795803, 15, 288, lower.tail=F)
```

```
[1] 0.02376003
```

```
# Regression

library(lmerTest)
```

```
Loading required package: lme4
```

```
Loading required package: Matrix
```

```
Attaching package: 'lmerTest'
```

```
The following object is masked from 'package:lme4':

    lmer
```

```
The following object is masked from 'package:stats':

    step
```

```
reg1 <- lmer(y~I+(1|T)+(1|I:T), contrasts=list(I=contr.sum), data=CrossedDataset1)
summary(reg1)
```

```
Linear mixed model fit by REML. t-tests use Satterthwaite's method [
lmerModLmerTest]
Formula: y ~ I + (1 | T) + (1 | I:T)
   Data: CrossedDataset1

REML criterion at convergence: 1316.5

Scaled residuals: 
    Min      1Q  Median      3Q     Max 
-3.2374 -0.6473 -0.0273  0.6261  3.5190 

Random effects:
 Groups   Name        Variance Std.Dev.
 I:T      (Intercept) 0.2848   0.5337  
 T        (Intercept) 0.4743   0.6887  
 Residual             3.1932   1.7869  
Number of obs: 320, groups:  I:T, 32; T, 16

Fixed effects:
            Estimate Std. Error      df t value Pr(>|t|)    
(Intercept)   6.5071     0.2203 14.9988   29.54 1.05e-14 ***
I1            0.6307     0.1374 15.0013    4.59 0.000354 ***
---
Signif. codes:  0 '***' 0.001 '**' 0.01 '*' 0.05 '.' 0.1 ' ' 1

Correlation of Fixed Effects:
   (Intr)
I1 0.000
```

```
ls_means(reg1, which = "I", pairwise = TRUE)
```

```
Least Squares Means table:

        Estimate Std. Error df t value   lower   upper  Pr(>|t|)    
I1 - I2  1.26148    0.27481 15  4.5904 0.67574 1.84721 0.0003536 ***
---
Signif. codes:  0 '***' 0.001 '**' 0.01 '*' 0.05 '.' 0.1 ' ' 1

  Confidence level: 95%
  Degrees of freedom method: Satterthwaite
```

```
ranova(reg1)
```

```
ANOVA-like table for random-effects: Single term deletions

Model:
y ~ I + (1 | T) + (1 | I:T)
          npar  logLik    AIC    LRT Df Pr(>Chisq)  
<none>       5 -658.24 1326.5                       
(1 | T)      4 -659.85 1327.7 3.2237  1    0.07258 .
(1 | I:T)    4 -660.00 1328.0 3.5294  1    0.06029 .
---
Signif. codes:  0 '***' 0.001 '**' 0.01 '*' 0.05 '.' 0.1 ' ' 1
```

```
# Note this analysis could have been done using the lme4 command rather than the 
# lmer command used here. The Satterthwaite degrees of freedom for the fixed effect
# of factor I are 15.0013 which is approximately 15 as expected. Note that t = 4.59
# (=sqrt(21.07)) and p = 0.000354 as before. The estimates of σ_u1^2,σ_v1^2 and 
# σ_e^2 are 0.4743, 0.2848 and 3.1932 respectively. Using Equations(4) (5) and (6),
# these give the estimates of ξ_T, ξ_I∧T and ξ_E  above precisely. As before, it can
# be seen that δ_0= 6.5071 and δ_1= 0.6307, and that 0.6307 is half the difference 
# in the mean of all outcomes for levels 1 and 2 of factor I given as 1.26148. Note
# that the standard error of δ_0 is 0.1374 which is also half of 0.27481.

# Note: using the following code to force a specific contrast gives the same output
reg1_contrasts <- lmer(y~contrast1+(1|T)+(1|I:T), data=CrossedDataset1)
summary(reg1_contrasts)
```

```
Linear mixed model fit by REML. t-tests use Satterthwaite's method [
lmerModLmerTest]
Formula: y ~ contrast1 + (1 | T) + (1 | I:T)
   Data: CrossedDataset1

REML criterion at convergence: 1316.5

Scaled residuals: 
    Min      1Q  Median      3Q     Max 
-3.2374 -0.6473 -0.0273  0.6261  3.5190 

Random effects:
 Groups   Name        Variance Std.Dev.
 I:T      (Intercept) 0.2848   0.5337  
 T        (Intercept) 0.4743   0.6887  
 Residual             3.1932   1.7869  
Number of obs: 320, groups:  I:T, 32; T, 16

Fixed effects:
            Estimate Std. Error      df t value Pr(>|t|)    
(Intercept)   6.5071     0.2203 14.9988   29.54 1.05e-14 ***
contrast1     0.6307     0.1374 15.0013    4.59 0.000354 ***
---
Signif. codes:  0 '***' 0.001 '**' 0.01 '*' 0.05 '.' 0.1 ' ' 1

Correlation of Fixed Effects:
          (Intr)
contrast1 0.000
```

```
ranova(reg1_contrasts)
```

```
ANOVA-like table for random-effects: Single term deletions

Model:
y ~ contrast1 + (1 | T) + (1 | I:T)
          npar  logLik    AIC    LRT Df Pr(>Chisq)  
<none>       5 -658.24 1326.5                       
(1 | T)      4 -659.85 1327.7 3.2237  1    0.07258 .
(1 | I:T)    4 -660.00 1328.0 3.5294  1    0.06029 .
---
Signif. codes:  0 '***' 0.001 '**' 0.01 '*' 0.05 '.' 0.1 ' ' 1
```

### b) Case Two - Three Interventions

```
CrossedDataset1_3Ints <- readRDS("P:/CTRU/Projects/Methodology/2_Statistical Methodology/2022_Rebecca Walwyn_NIHR Advanced Fellowship/7_Papers/2A_Crossed Therapist Designs/4_Submitted_20250930/3_Uploaded to GitHub/CrossedDataset1_3Ints.rds")

# ANOVA – Overall

aov1_3ints_1 <- aov(y~I+Error(T+I:T), data=CrossedDataset1_3Ints)
summary(aov1_3ints_1)
```

```
Error: T
          Df Sum Sq Mean Sq F value Pr(>F)
Residuals 15  127.7   8.516               

Error: T:I
          Df Sum Sq Mean Sq F value   Pr(>F)    
I          2  323.5  161.75   28.96 9.89e-08 ***
Residuals 30  167.6    5.59                     
---
Signif. codes:  0 '***' 0.001 '**' 0.01 '*' 0.05 '.' 0.1 ' ' 1

Error: Within
           Df Sum Sq Mean Sq F value Pr(>F)
Residuals 432   1377   3.187
```

```
print(model.tables(aov1_3ints_1,"means"))
```

```
Tables of means
Grand mean
         
7.891846 

 I 
I
    1     2     3 
8.836 6.835 8.005
```

```
# Note that the fixed effect of factor I appears in the W_I∧T stratum, where the 
# residual degrees of freedom are 30. Note also, for the overall fixed effect of 
# factor I, F = 28.96 on 2 and 30 degrees of freedom, and p = 0.0000000989. It can 
# be seen that the estimates of ξ_T, ξ_I∧T and ξ_E  are 8.516, 5.59 and 3.187 
# respectively.

# For the random effect of factor T, F = 8.516/5.59 = 1.52343470483 on 15 and 30 
# degrees of freedom

pf(1.52343470483, 15, 30, lower.tail=F)
```

```
[1] 0.1588289
```

```
# For the random effect of factor I∧T, F = 5.59/3.187 = 1.75400062754 on 30 and 432
# degrees of freedom

pf(1.75400062754, 30, 432, lower.tail=F)
```

```
[1] 0.009190573
```

```
# Note that no specific contrasts are given in the output. The coefficients command
# does not give the orthogonal contrasts of interest so are not provided here.

# It may be desirable to fit specific contrasts of interest – first contrast1 and 
# contrast2

aov1_3ints_2 <- aov(y~contrast1+contrast2+Error(T+I:T), data=CrossedDataset1_3Ints)
summary(aov1_3ints_2)
```

```
Error: T
          Df Sum Sq Mean Sq F value Pr(>F)
Residuals 15  127.7   8.516               

Error: T:I
          Df Sum Sq Mean Sq F value   Pr(>F)    
contrast1  1    3.1     3.1    0.55    0.464    
contrast2  1  320.4   320.4   57.37 1.91e-08 ***
Residuals 30  167.6     5.6                     
---
Signif. codes:  0 '***' 0.001 '**' 0.01 '*' 0.05 '.' 0.1 ' ' 1

Error: Within
           Df Sum Sq Mean Sq F value Pr(>F)
Residuals 432   1377   3.187
```

```
#Then contrast2 and contrast3

aov1_3ints_3 <- aov(y~contrast2+contrast3+Error(T+I:T), data=CrossedDataset1_3Ints)
summary(aov1_3ints_3)
```

```
Error: T
          Df Sum Sq Mean Sq F value Pr(>F)
Residuals 15  127.7   8.516               

Error: T:I
          Df Sum Sq Mean Sq F value   Pr(>F)    
contrast2  1  320.4   320.4   57.37 1.91e-08 ***
contrast3  1    3.1     3.1    0.55    0.464    
Residuals 30  167.6     5.6                     
---
Signif. codes:  0 '***' 0.001 '**' 0.01 '*' 0.05 '.' 0.1 ' ' 1

Error: Within
           Df Sum Sq Mean Sq F value Pr(>F)
Residuals 432   1377   3.187
```

```
# Note whether contrast1 or contrast3 is included makes no difference in the ANOVA 
# model. Note also that the sums of squares for the two orthogonal contrasts (i.e.,
# 320.4 and 3.1) add up to the sum of squares for the overall effect of factor I 
# (i.e., 323.5).

#Regression - first contrast1 and contrast2

library(lmerTest)
reg1_3ints_1 <- lmer(y~contrast1+contrast2+(1|T)+(1|I:T), data
                     =CrossedDataset1_3Ints)
summary(reg1_3ints_1)
```

```
Linear mixed model fit by REML. t-tests use Satterthwaite's method [
lmerModLmerTest]
Formula: y ~ contrast1 + contrast2 + (1 | T) + (1 | I:T)
   Data: CrossedDataset1_3Ints

REML criterion at convergence: 1957

Scaled residuals: 
    Min      1Q  Median      3Q     Max 
-2.7031 -0.6476 -0.0205  0.6627  3.4115 

Random effects:
 Groups   Name        Variance Std.Dev.
 I:T      (Intercept) 0.23978  0.4897  
 T        (Intercept) 0.09769  0.3126  
 Residual             3.18725  1.7853  
Number of obs: 480, groups:  I:T, 48; T, 16

Fixed effects:
            Estimate Std. Error       df t value Pr(>|t|)    
(Intercept)  7.89185    0.13320 14.99999  59.249  < 2e-16 ***
contrast1   -0.05658    0.07627 30.00004  -0.742    0.464    
contrast2    1.00067    0.13211 30.00004   7.575 1.91e-08 ***
---
Signif. codes:  0 '***' 0.001 '**' 0.01 '*' 0.05 '.' 0.1 ' ' 1

Correlation of Fixed Effects:
          (Intr) cntrs1
contrast1 0.000        
contrast2 0.000  0.000
```

```
ranova(reg1_3ints_1)
```

```
ANOVA-like table for random-effects: Single term deletions

Model:
y ~ contrast1 + contrast2 + (1 | T) + (1 | I:T)
          npar  logLik    AIC    LRT Df Pr(>Chisq)  
<none>       6 -978.48 1969.0                       
(1 | T)      5 -978.95 1967.9 0.9265  1    0.33578  
(1 | I:T)    5 -981.09 1972.2 5.2072  1    0.02249 *
---
Signif. codes:  0 '***' 0.001 '**' 0.01 '*' 0.05 '.' 0.1 ' ' 1
```

```
# Note, as in the two interventions case, this analysis could have been done using 
# the lme4 command rather than the lmer command used here. The Satterthwaite 
# degrees of freedom for the orthogonal contrasts of factor I are 30.00004 which is
# approximately 30 as expected. The estimates of σ_u1^2, σ_v1^2 and σ_e^2 are 
# 0.09769, 0.23978 and 3.18725 respectively. Using Equations (4), (5) and (6), 
# these give the estimates of ξ_T, ξ_I∧T and ξ_E above precisely. It can be seen 
# that δ_0=7.89185 (the grand mean). 

# The estimate for contrast1 is given by 1/6*[1*(8.836)+1*(6.835)-2*(8.005)]=-0.339
# /6=-0.0565. Equivalently, it is half the difference between the grand mean 
# (7.89185) and the mean of all outcomes for level 3 of factor I (8.005), which 
# equals -0.056575. The estimate of contrast2 is given by 1/2*[1*(8.836)-1*(6.835)
# +0*(8.005)]=2.001/2=1.00005. This is half the difference between the mean of all 
# outcomes for levels 1 (8.836) and 2 (6.835) of factor I. 

# We then fit the regression model using contrast2 and contrast3

reg1_3ints_2 <- lmer(y~contrast2+contrast3+(1|T)+(1|I:T), data
                     =CrossedDataset1_3Ints)
summary(reg1_3ints_2)
```

```
Linear mixed model fit by REML. t-tests use Satterthwaite's method [
lmerModLmerTest]
Formula: y ~ contrast2 + contrast3 + (1 | T) + (1 | I:T)
   Data: CrossedDataset1_3Ints

REML criterion at convergence: 1955.6

Scaled residuals: 
    Min      1Q  Median      3Q     Max 
-2.7031 -0.6476 -0.0205  0.6627  3.4115 

Random effects:
 Groups   Name        Variance Std.Dev.
 I:T      (Intercept) 0.23978  0.4897  
 T        (Intercept) 0.09769  0.3126  
 Residual             3.18725  1.7853  
Number of obs: 480, groups:  I:T, 48; T, 16

Fixed effects:
            Estimate Std. Error      df t value Pr(>|t|)    
(Intercept)   7.8918     0.1332 15.0000  59.249  < 2e-16 ***
contrast2     1.0007     0.1321 30.0000   7.575 1.91e-08 ***
contrast3    -0.1132     0.1525 30.0000  -0.742    0.464    
---
Signif. codes:  0 '***' 0.001 '**' 0.01 '*' 0.05 '.' 0.1 ' ' 1

Correlation of Fixed Effects:
          (Intr) cntrs2
contrast2 0.000        
contrast3 0.000  0.000
```

```
# Note that the model is unchanged. The only difference is the estimate for 
# contrast3, which  is given by 3/2*[0.5*(8.836)+0.5*(6.835)-1*(8.005)]=-0.1695/1.5
# =-0.113. Equivalently, this is the difference between the grand mean (7.89185) 
# and the mean of all outcomes for level 3 of  factor I (8.005), which equals 
# -0.11315.

# Note: where factor I has 3 levels, the following code gives i) the difference 
# between the mean of all outcomes for level 1 of factor I (8.836) and the grand 
# mean (7.891846), which equals 0.944154, and ii) the difference between the mean 
# of all outcomes for level 2 of factor I (6.835) and the grand mean (7.891846), 
# which equals -1.056846. Note that these contrasts are not orthogonal and are 
# hence not recommended unless specifically of interest.

reg1_3ints_3<-lmer(y~I+(1|T)+(1|I:T),contrasts=list(I=contr.sum),data
                   =CrossedDataset1_3Ints)
summary(reg1_3ints_3)
```

```
Linear mixed model fit by REML. t-tests use Satterthwaite's method [
lmerModLmerTest]
Formula: y ~ I + (1 | T) + (1 | I:T)
   Data: CrossedDataset1_3Ints

REML criterion at convergence: 1955.6

Scaled residuals: 
    Min      1Q  Median      3Q     Max 
-2.7031 -0.6476 -0.0205  0.6627  3.4115 

Random effects:
 Groups   Name        Variance Std.Dev.
 I:T      (Intercept) 0.23978  0.4897  
 T        (Intercept) 0.09769  0.3126  
 Residual             3.18725  1.7853  
Number of obs: 480, groups:  I:T, 48; T, 16

Fixed effects:
            Estimate Std. Error      df t value Pr(>|t|)    
(Intercept)   7.8918     0.1332 15.0000  59.249  < 2e-16 ***
I1            0.9441     0.1525 30.0000   6.189 8.23e-07 ***
I2           -1.0572     0.1525 30.0000  -6.931 1.07e-07 ***
---
Signif. codes:  0 '***' 0.001 '**' 0.01 '*' 0.05 '.' 0.1 ' ' 1

Correlation of Fixed Effects:
   (Intr) I1    
I1  0.000       
I2  0.000 -0.500
```

```
# Hence, we recommended that the specific contrasts of interest are entered into 
# the ANOVA or regression model directly.
```

## **2. Randomised Block Factorial Design**

### a) Case One - Two Interventions Only

```
CrossedDataset2 <- readRDS("P:/CTRU/Projects/Methodology/2_Statistical Methodology/2022_Rebecca Walwyn_NIHR Advanced Fellowship/7_Papers/2A_Crossed Therapist Designs/4_Submitted_20250930/3_Uploaded to GitHub/CrossedDataset2.rds")

# ANOVA

aov2 <- aov(y~I+Error(I*T*B), contrasts=list(I=contr.sum), data=CrossedDataset2)
summary(aov2)
```

```
Error: I
  Df Sum Sq Mean Sq
I  1  231.9   231.9

Error: T
          Df Sum Sq Mean Sq F value Pr(>F)
Residuals 15  432.8   28.85               

Error: B
          Df Sum Sq Mean Sq F value Pr(>F)
Residuals  4  316.5   79.13               

Error: I:T
          Df Sum Sq Mean Sq F value Pr(>F)
Residuals 15  83.53   5.569               

Error: I:B
          Df Sum Sq Mean Sq F value Pr(>F)
Residuals  4  100.3   25.08               

Error: T:B
          Df Sum Sq Mean Sq F value Pr(>F)
Residuals 60  260.3   4.338               

Error: I:T:B
          Df Sum Sq Mean Sq F value Pr(>F)
Residuals 60  213.2   3.553               

Error: Within
           Df Sum Sq Mean Sq F value Pr(>F)
Residuals 160  466.5   2.916
```

```
coefficients(aov2)
```

```
(Intercept) :
(Intercept) 
   19.42835 

I :
       I1 
-0.851298 

T :
numeric(0)

B :
numeric(0)

I:T :
numeric(0)

I:B :
numeric(0)

T:B :
numeric(0)

I:T:B :
numeric(0)

Within :
numeric(0)
```

```
print(model.tables(aov2,"means"))
```

```
Tables of means
Grand mean
         
19.42835 

 I 
I
     1      2 
18.577 20.280
```

```
# Note that the fixed effect of factor I appears in the W_I stratum, where there 
# are no residual degrees of freedom. It can be seen that estimates of ξ_T,ξ_B,ξ_I∧T
# ξ_I∧B,ξ_T∧B,ξ_I∧T∧B and ξ_E are 28.85, 79.13, 5.569, 25.08, 4.338, 3.553 and 2.916
# respectively. As such, the estimate of ξ_I = 5.569+25.08-3.553=27.0983 so the 
# approximate F is 231.9/27.0983=8.558. Using Equation (1), the denominator 
# degrees of freedom for this approximate F test are 4.6022. The p-value can be    
# obtained using

pf(8.557732404, 1, 4.602238732, lower.tail=F)
```

```
[1] 0.03625654
```

```
# Note that the estimates of δ_0=19.42835 and δ_1=-0.851298. Finally note that 
# -0.851298 is half the difference between the mean of all outcomes on level 1 of 
# factor I (18.577) and the mean of all outcomes on level 2 of factor I (20.280).

# For the random effect of factor T, F = 28.85/6.354 = 12.45357255 on 15 and 
# 15.57841919 degrees of freedom, so the p-value is

pf(12.45357255, 15, 15.57841919, lower.tail=F)
```

```
[1] 5.264814e-06
```

```
# For the random effect of factor B, F = 79.13/25.865 = 3.059346607 on 4 and 
# 4.240187202 degrees of freedom, so the p-value is

pf(3.059346607, 4, 4.240187202, lower.tail=F)
```

```
[1] 0.1448388
```

```
# For the random effect of factor I∧T, F = 5.569/3.553 = 1.567407824 on 15 and 60 
# degrees of freedom, so the p-value is

pf(1.567407824, 15, 60, lower.tail=F)
```

```
[1] 0.1109303
```

```
# For the random effect of factor I∧B, F = 25.08/3.553 = 7.058823529 on 4 and 60 
# degrees of freedom, so the p-value is

pf(7.058823529, 4, 60, lower.tail=F)
```

```
[1] 0.0001001321
```

```
# For the random effect of factor T∧B, F = 4.338/3.553 = 1.220940051 on 60 and 60 
# degrees of freedom, so the p-value is

pf(1.220940051, 60, 60, lower.tail=F)
```

```
[1] 0.2208637
```

```
# For the random effect of factor I∧T∧B, F = 3.553/2.916 = 1.218449931 on 60 and 
# 160 degrees of freedom, so the p-value is

pf(1.218449931, 60, 160, lower.tail=F)
```

```
[1] 0.1669236
```

```
#Regression

library(lmerTest)
reg2 <- lmer(y~I+(1|T)+(1|B)+(1|T:B)+(1|I:T)+(1|I:B)+(1|I:T:B), 
             contrasts=list(I=contr.sum), data=CrossedDataset2)
summary(reg2)
```

```
Linear mixed model fit by REML. t-tests use Satterthwaite's method [
lmerModLmerTest]
Formula: y ~ I + (1 | T) + (1 | B) + (1 | T:B) + (1 | I:T) + (1 | I:B) +  
    (1 | I:T:B)
   Data: CrossedDataset2

REML criterion at convergence: 1355.9

Scaled residuals: 
     Min       1Q   Median       3Q      Max 
-2.62524 -0.62184 -0.02473  0.60456  2.68687 

Random effects:
 Groups   Name        Variance Std.Dev.
 I:T:B    (Intercept) 0.3185   0.5644  
 T:B      (Intercept) 0.1963   0.4431  
 I:T      (Intercept) 0.2016   0.4490  
 T        (Intercept) 1.1249   1.0606  
 I:B      (Intercept) 0.6728   0.8203  
 B        (Intercept) 0.8322   0.9123  
 Residual             2.9157   1.7075  
Number of obs: 320, groups:  I:T:B, 160; T:B, 80; I:T, 32; T, 16; I:B, 10; B, 5

Fixed effects:
            Estimate Std. Error      df t value Pr(>|t|)    
(Intercept)  19.4283     0.5691  6.6256  34.138 1.07e-08 ***
I1           -0.8513     0.2910  4.6021  -2.925   0.0363 *  
---
Signif. codes:  0 '***' 0.001 '**' 0.01 '*' 0.05 '.' 0.1 ' ' 1

Correlation of Fixed Effects:
   (Intr)
I1 0.000
```

```
ls_means(reg2, which = "I", pairwise = TRUE)
```

```
Least Squares Means table:

        Estimate Std. Error  df t value    lower    upper Pr(>|t|)  
I1 - I2 -1.70260    0.58202 4.6 -2.9253 -3.23848 -0.16671  0.03626 *
---
Signif. codes:  0 '***' 0.001 '**' 0.01 '*' 0.05 '.' 0.1 ' ' 1

  Confidence level: 95%
  Degrees of freedom method: Satterthwaite
```

```
ranova(reg2)
```

```
boundary (singular) fit: see help('isSingular')
```

```
ANOVA-like table for random-effects: Single term deletions

Model:
y ~ I + (1 | T) + (1 | B) + (1 | T:B) + (1 | I:T) + (1 | I:B) + (1 | I:T:B)
            npar  logLik    AIC     LRT Df Pr(>Chisq)    
<none>         9 -677.94 1373.9                          
(1 | T)        8 -682.24 1380.5  8.6062  1  0.0033502 ** 
(1 | B)        8 -678.55 1373.1  1.2214  1  0.2690822    
(1 | T:B)      8 -678.23 1372.5  0.5974  1  0.4395841    
(1 | I:T)      8 -678.60 1373.2  1.3206  1  0.2504858    
(1 | I:B)      8 -684.42 1384.8 12.9659  1  0.0003172 ***
(1 | I:T:B)    8 -678.37 1372.8  0.8768  1  0.3490691    
---
Signif. codes:  0 '***' 0.001 '**' 0.01 '*' 0.05 '.' 0.1 ' ' 1
```

```
# Note: this analysis should be done with the lmer command used here. Satterthwaite
# degrees of  freedom for the fixed effect of I are 4.6021 as expected. Note that t
# =-2.925 (=sqrt(8.558)) and p=0.0363 as before. It can be seen that estimates of 
# σ_u1^2, σ_u2^2, σ_u3^2, σ_v1^2, σ_v2^2, σ_v3^2 and σ_e^2 are 1.1249, 0.8322, 
# 0.1963, 0.2016, 0.6728, 0.3185 and 2.9157, respectively. Using Equations (E.1) to
# (E.7), these give the estimates of ξ_T,ξ_B,ξ_I∧T,ξ_I∧B,ξ_T∧B,ξ_I∧T∧B and ξ_E  
# above precisely. As before, it can be seen that estimates of δ_0=19.4283 and δ_1= 
# -0.8513, and that -0.8513 is half the difference between the means of all 
# outcomes on levels 1 and 2 of factor I, given as -1.70260. Note that the standard
# error of the estimate of δ_0 is 0.2910 which is also half 0.58202.

# ANOVA (again)

# It is of interest to see what R does if factor I is only included as a fixed 
# effect and the  order of the random effects is varied. See below.    

aov2a<-aov(y~I+Error(T*B+I:T+I:B+I:T:B), contrasts=list(I=contr.sum),
           data=CrossedDataset2)
summary(aov2a)
```

```
Error: T
          Df Sum Sq Mean Sq F value Pr(>F)
Residuals 15  432.8   28.85               

Error: B
          Df Sum Sq Mean Sq F value Pr(>F)
Residuals  4  316.5   79.13               

Error: T:B
          Df Sum Sq Mean Sq F value Pr(>F)
Residuals 60  260.3   4.338               

Error: T:I
          Df Sum Sq Mean Sq F value   Pr(>F)    
I          1 231.91  231.91   41.65 1.09e-05 ***
Residuals 15  83.53    5.57                     
---
Signif. codes:  0 '***' 0.001 '**' 0.01 '*' 0.05 '.' 0.1 ' ' 1

Error: B:I
          Df Sum Sq Mean Sq F value Pr(>F)
Residuals  4  100.3   25.08               

Error: T:B:I
          Df Sum Sq Mean Sq F value Pr(>F)
Residuals 60  213.2   3.553               

Error: Within
           Df Sum Sq Mean Sq F value Pr(>F)
Residuals 160  466.5   2.916
```

```
aov2b<-aov(y~I+Error(T+B+I:B+I:T:B+T:B+I:T),contrasts=list(I=contr.sum),
           data=CrossedDataset2)
summary(aov2b)
```

```
Error: T
          Df Sum Sq Mean Sq F value Pr(>F)
Residuals 15  432.8   28.85               

Error: B
          Df Sum Sq Mean Sq F value Pr(>F)
Residuals  4  316.5   79.13               

Error: B:I
          Df Sum Sq Mean Sq F value Pr(>F)  
I          1  231.9  231.91   9.246 0.0384 *
Residuals  4  100.3   25.08                 
---
Signif. codes:  0 '***' 0.001 '**' 0.01 '*' 0.05 '.' 0.1 ' ' 1

Error: T:B
          Df Sum Sq Mean Sq F value Pr(>F)
Residuals 60  260.3   4.338               

Error: T:I
          Df Sum Sq Mean Sq F value Pr(>F)
Residuals 15  83.53   5.569               

Error: T:B:I
          Df Sum Sq Mean Sq F value Pr(>F)
Residuals 60  213.2   3.553               

Error: Within
           Df Sum Sq Mean Sq F value Pr(>F)
Residuals 160  466.5   2.916
```

```
# Note: the output here shows that the fixed effect of factor I incorrectly appears
# in the W_I∧T or W_I∧C stratum, depending on whether I:T or I:C appears in the 
# random part of the model statement first, respectively.
```

### b) Case Two - Three Interventions

```
CrossedDataset2_3Ints <- readRDS("P:/CTRU/Projects/Methodology/2_Statistical Methodology/2022_Rebecca Walwyn_NIHR Advanced Fellowship/7_Papers/2A_Crossed Therapist Designs/4_Submitted_20250930/3_Uploaded to GitHub/CrossedDataset2_3Ints.rds")

# ANOVA

aov2_3ints_1<-aov(y~I+Error(I*T*B), data=CrossedDataset2_3Ints)
summary(aov2_3ints_1)
```

```
Error: I
  Df Sum Sq Mean Sq
I  2   59.4    29.7

Error: T
          Df Sum Sq Mean Sq F value Pr(>F)
Residuals 15  133.6   8.907               

Error: B
          Df Sum Sq Mean Sq F value Pr(>F)
Residuals  4  128.2   32.04               

Error: I:T
          Df Sum Sq Mean Sq F value Pr(>F)
Residuals 30  156.7   5.222               

Error: I:B
          Df Sum Sq Mean Sq F value Pr(>F)
Residuals  8    130   16.25               

Error: T:B
          Df Sum Sq Mean Sq F value Pr(>F)
Residuals 60  377.1   6.286               

Error: I:T:B
           Df Sum Sq Mean Sq F value Pr(>F)
Residuals 120  510.7   4.256               

Error: Within
           Df Sum Sq Mean Sq F value Pr(>F)
Residuals 240  996.5   4.152
```

```
print(model.tables(aov2_3ints_1,"means"))
```

```
Tables of means
Grand mean
         
18.64557 

 I 
I
     1      2      3 
19.141 18.363 18.432
```

```
# Note that the fixed effect of factor I appears in the W_I stratum, where there 
# are no residual degrees of freedom. It can be seen that estimates of ξ_T, ξ_B, 
# ξ_I∧T, ξ_I∧B, ξ_T∧B, ξ_I∧T∧B and ξ_E are 8.907, 32.04, 5.222, 16.25, 6.286, 4.256 
# and 4.152 respectively. As such, the estimate of ξ_I = 5.222+16.25-4.256=17.21797 
# so the approximate F is 29.7/17.218=1.725. Using Equation (1), the denominator 
# degrees of freedom for this approximate F test are 8.700. The p-value can be
# obtained using

pf(1.724942023, 2, 8.700040024, lower.tail=F)
```

```
[1] 0.2338942
```

```
# For the random effect of factor T, F = 8.907/7.252 = 1.228212907 on 15 and 
# 30.60340223 degrees of freedom, so the p-value is

pf(1.228212907, 15, 30.60340223, lower.tail=F)
```

```
[1] 0.30431
```

```
# For the random effect of factor B, F = 32.04/18.28 = 1.75273523 on 4 and 
# 9.881279215 degrees of freedom, so the p-value is

pf(1.75273523, 4, 9.881279215, lower.tail=F)
```

```
[1] 0.2156927
```

```
# For the random effect of factor I∧T, F = 5.222/4.256 = 1.226973684 on 30 and 120 
# degrees of freedom, so the p-value is

pf(1.226973684, 30, 120, lower.tail=F)
```

```
[1] 0.218337
```

```
# For the random effect of factor I∧B, F = 16.25/4.256 = 3.818139098 on 8 and 120 
# degrees of freedom, so the p-value is

pf(3.818139098, 8, 120, lower.tail=F)
```

```
[1] 0.0004985334
```

```
# For the random effect of factor T∧B, F = 6.286/4.256 = 1.476973684 on 60 and 120 
# degrees of freedom, so the p-value is

pf(1.476973684, 60, 120, lower.tail=F)
```

```
[1] 0.03616006
```

```
# For the random effect of factor I∧T∧B, F = 4.256/4.152 = 1.02504817 on 120 and 
# 240 degrees of freedom, so the p-value is

pf(1.02504817, 120, 240, lower.tail=F)
```

```
[1] 0.430919
```

```
# Note that no specific contrasts are given in the output. The coefficients command
# does not  give the orthogonal contrasts of interest so are not provided here. 

# It may be desirable to fit specific contrasts of interest – first contrast1 and 
# contrast2

aov2_3ints_2<-aov(y~contrast1+contrast2+Error(I*T*B), data=CrossedDataset2_3Ints)
summary(aov2_3ints_2)
```

```
Error: I
          Df Sum Sq Mean Sq
contrast1  1  10.96   10.96
contrast2  1  48.44   48.44

Error: T
          Df Sum Sq Mean Sq F value Pr(>F)
Residuals 15  133.6   8.907               

Error: B
          Df Sum Sq Mean Sq F value Pr(>F)
Residuals  4  128.2   32.04               

Error: I:T
          Df Sum Sq Mean Sq F value Pr(>F)
Residuals 30  156.7   5.222               

Error: I:B
          Df Sum Sq Mean Sq F value Pr(>F)
Residuals  8    130   16.25               

Error: T:B
          Df Sum Sq Mean Sq F value Pr(>F)
Residuals 60  377.1   6.286               

Error: I:T:B
           Df Sum Sq Mean Sq F value Pr(>F)
Residuals 120  510.7   4.256               

Error: Within
           Df Sum Sq Mean Sq F value Pr(>F)
Residuals 240  996.5   4.152
```

```
# Then contrast2 and contrast3

aov2_3ints_3<-aov(y~contrast2+contrast3+Error(I*T*B), data=CrossedDataset2_3Ints)
summary(aov2_3ints_3)
```

```
Error: I
          Df Sum Sq Mean Sq
contrast2  1  48.44   48.44
contrast3  1  10.96   10.96

Error: T
          Df Sum Sq Mean Sq F value Pr(>F)
Residuals 15  133.6   8.907               

Error: B
          Df Sum Sq Mean Sq F value Pr(>F)
Residuals  4  128.2   32.04               

Error: I:T
          Df Sum Sq Mean Sq F value Pr(>F)
Residuals 30  156.7   5.222               

Error: I:B
          Df Sum Sq Mean Sq F value Pr(>F)
Residuals  8    130   16.25               

Error: T:B
          Df Sum Sq Mean Sq F value Pr(>F)
Residuals 60  377.1   6.286               

Error: I:T:B
           Df Sum Sq Mean Sq F value Pr(>F)
Residuals 120  510.7   4.256               

Error: Within
           Df Sum Sq Mean Sq F value Pr(>F)
Residuals 240  996.5   4.152
```

```
# Note whether contrast1 or contrast3 is included makes no difference to the ANOVA 
# model. The sums of squares for the two orthogonal contrasts (i.e., 48.44 and 
# 10.96) add up to the sum of squares for the overall effect of factor I (i.e., 
# 59.4).

# For the fixed effect of contrast1, F = 10.96/17.21797 = 0.63654426160 on 1 and 
# 8.700040024 degrees of freedom, so the p-value is

pf(0.63654426160, 1, 8.700040024, lower.tail=F)
```

```
[1] 0.4461939
```

```
# For the fixed effect of contrast2, F = 48.44/17.21797 = 2.81333978395 on 1 and 
# 8.700040024 degrees of freedom, so the p-value is

pf(2.81333978395, 1, 8.700040024, lower.tail=F)
```

```
[1] 0.1289596
```

```
# Regression – first contrast1 and contrast2

library(lmerTest)
reg2_3ints_1<-lmer(y~contrast1+contrast2+(1|T)+(1|B)+(1|T:B)+(1|I:T)+(1|I:B)
                   +(1|I:T:B), data=CrossedDataset2_3Ints)
summary(reg2_3ints_1)
```

```
Linear mixed model fit by REML. t-tests use Satterthwaite's method [
lmerModLmerTest]
Formula: y ~ contrast1 + contrast2 + (1 | T) + (1 | B) + (1 | T:B) + (1 |  
    I:T) + (1 | I:B) + (1 | I:T:B)
   Data: CrossedDataset2_3Ints

REML criterion at convergence: 2116.8

Scaled residuals: 
     Min       1Q   Median       3Q      Max 
-2.16739 -0.66409 -0.04268  0.58173  2.88999 

Random effects:
 Groups   Name        Variance Std.Dev.
 I:T:B    (Intercept) 0.05181  0.2276  
 T:B      (Intercept) 0.33835  0.5817  
 I:T      (Intercept) 0.09663  0.3109  
 T        (Intercept) 0.05517  0.2349  
 I:B      (Intercept) 0.37488  0.6123  
 B        (Intercept) 0.14332  0.3786  
 Residual             4.15189  2.0376  
Number of obs: 480, groups:  I:T:B, 240; T:B, 80; I:T, 48; T, 16; I:B, 15; B, 5

Fixed effects:
            Estimate Std. Error      df t value Pr(>|t|)    
(Intercept)  18.6456     0.2687  4.5754  69.385  4.3e-08 ***
contrast1     0.1068     0.1339  8.7002   0.798    0.446    
contrast2     0.3891     0.2320  8.7002   1.677    0.129    
---
Signif. codes:  0 '***' 0.001 '**' 0.01 '*' 0.05 '.' 0.1 ' ' 1

Correlation of Fixed Effects:
          (Intr) cntrs1
contrast1 0.000        
contrast2 0.000  0.000
```

```
ranova(reg2_3ints_1)
```

```
ANOVA-like table for random-effects: Single term deletions

Model:
y ~ contrast1 + contrast2 + (1 | T) + (1 | B) + (1 | T:B) + (1 | I:T) + (1 | I:B) + (1 | I:T:B)
            npar  logLik    AIC     LRT Df Pr(>Chisq)   
<none>        10 -1058.4 2136.8                         
(1 | T)        9 -1058.5 2135.0  0.2207  1   0.638537   
(1 | B)        9 -1058.6 2135.2  0.4767  1   0.489911   
(1 | T:B)      9 -1060.0 2137.9  3.1602  1   0.075453 . 
(1 | I:T)      9 -1058.7 2135.3  0.5230  1   0.469551   
(1 | I:B)      9 -1063.4 2144.8 10.0514  1   0.001522 **
(1 | I:T:B)    9 -1058.4 2134.8  0.0244  1   0.875897   
---
Signif. codes:  0 '***' 0.001 '**' 0.01 '*' 0.05 '.' 0.1 ' ' 1
```

```
# Note: this analysis should be done with the lmer command used here. Satterthwaite
# degrees of  freedom for the fixed effect of I are 8.7002 as expected. Note that 
# for contrast1 t=0.798 (=sqrt(0.63654426160)) and p=0.446 as before. Note that for
# contrast2 t=1.677(=sqrt(2.81333978395)) and p=0.129 as before.

# It can be seen that estimates of σ_u1^2, σ_u2^2, σ_u3^2, σ_v1^2, σ_v2^2, σ_v3^2 
# and σ_e^2 are 0.05517, 0.14332, 0.33835, 0.09663, 0.37488, 0.05181 and 4.15189 
# respectively. Using Equations (E.1) to (E.7), these give the estimates of ξ_T, 
# ξ_B,ξ_I∧T,ξ_I∧B,ξ_T∧B,ξ_I∧T∧B and ξ_E above precisely.
 
# It can be seen that δ_0=18.6456 (the grand mean). The estimate for contrast1 is 
# given by 1/6*[1*(19.141)+1*(18.363)-2*(18.432)]=0.64/6=0.10667. Equivalently, it 
# is half the difference between the grand mean (18.6456) and the mean of all 
# outcomes for level 3 of factor I (18.432), which equals 0.1068. The estimate of 
# contrast2 is given by 1/2*[1*(19.141)-1*(18.363)+0*(18.432)]=0.778/2=0.389. This 
# is half the difference between the mean of all outcomes for levels 1 (19.141) and
# 2 (18.363) of factor I. 

# We then fit the regression model using contrast2 and contrast3

reg2_3ints_2<-lmer(y~contrast2+contrast3+(1|T)+(1|B)+(1|T:B)+(1|I:T)+(1|I:B)
                   +(1|I:T:B), data=CrossedDataset2_3Ints)
summary(reg2_3ints_2)
```

```
Linear mixed model fit by REML. t-tests use Satterthwaite's method [
lmerModLmerTest]
Formula: y ~ contrast2 + contrast3 + (1 | T) + (1 | B) + (1 | T:B) + (1 |  
    I:T) + (1 | I:B) + (1 | I:T:B)
   Data: CrossedDataset2_3Ints

REML criterion at convergence: 2115.4

Scaled residuals: 
     Min       1Q   Median       3Q      Max 
-2.16739 -0.66409 -0.04268  0.58173  2.88999 

Random effects:
 Groups   Name        Variance Std.Dev.
 I:T:B    (Intercept) 0.05181  0.2276  
 T:B      (Intercept) 0.33835  0.5817  
 I:T      (Intercept) 0.09663  0.3109  
 T        (Intercept) 0.05517  0.2349  
 I:B      (Intercept) 0.37488  0.6123  
 B        (Intercept) 0.14332  0.3786  
 Residual             4.15189  2.0376  
Number of obs: 480, groups:  I:T:B, 240; T:B, 80; I:T, 48; T, 16; I:B, 15; B, 5

Fixed effects:
            Estimate Std. Error      df t value Pr(>|t|)    
(Intercept)  18.6456     0.2687  4.5753  69.385  4.3e-08 ***
contrast2     0.3891     0.2320  8.7002   1.677    0.129    
contrast3     0.2137     0.2678  8.7002   0.798    0.446    
---
Signif. codes:  0 '***' 0.001 '**' 0.01 '*' 0.05 '.' 0.1 ' ' 1

Correlation of Fixed Effects:
          (Intr) cntrs2
contrast2 0.000        
contrast3 0.000  0.000
```

```
# Note that the model is unchanged. The only difference is the estimate for 
# contrast3, which is given by 3/2*[0.5*(19.141)+0.5*(18.363)-1*(18.432)]=0.32/1.5
# =0.21333. Equivalently, this  is the difference between the grand mean (18.6456) 
# and the mean of all outcomes for level 3  of factor I (18.432), which equals 
# 0.2136.

# Note: where factor I has 3 levels, the following code gives i) the difference 
# between the mean of all outcomes for level 1 of factor I (19.141) and the grand 
# mean (18.6456), which equals 0.4954, and ii) the difference between the mean of 
# all outcomes for level 2 of factor I (18.363) and the grand mean (18.6456), which
# equals -0.2826. Note that these contrasts are not orthogonal and are hence not 
# recommended unless specifically of interest.  

reg2_3ints_3<-lmer(y~I+(1|T)+(1|B)+(1|T:B)+(1|I:T)+(1|I:B)+(1|I:T:B),
                   contrasts=list(I=contr.sum),data=CrossedDataset2_3Ints)
summary(reg2_3ints_3)
```

```
Linear mixed model fit by REML. t-tests use Satterthwaite's method [
lmerModLmerTest]
Formula: y ~ I + (1 | T) + (1 | B) + (1 | T:B) + (1 | I:T) + (1 | I:B) +  
    (1 | I:T:B)
   Data: CrossedDataset2_3Ints

REML criterion at convergence: 2115.4

Scaled residuals: 
     Min       1Q   Median       3Q      Max 
-2.16739 -0.66409 -0.04268  0.58173  2.88999 

Random effects:
 Groups   Name        Variance Std.Dev.
 I:T:B    (Intercept) 0.05181  0.2276  
 T:B      (Intercept) 0.33835  0.5817  
 I:T      (Intercept) 0.09663  0.3109  
 T        (Intercept) 0.05517  0.2349  
 I:B      (Intercept) 0.37488  0.6123  
 B        (Intercept) 0.14332  0.3786  
 Residual             4.15189  2.0376  
Number of obs: 480, groups:  I:T:B, 240; T:B, 80; I:T, 48; T, 16; I:B, 15; B, 5

Fixed effects:
            Estimate Std. Error      df t value Pr(>|t|)    
(Intercept)  18.6456     0.2687  4.5754  69.385  4.3e-08 ***
I1            0.4959     0.2678  8.7002   1.851   0.0983 .  
I2           -0.2822     0.2678  8.7002  -1.054   0.3204    
---
Signif. codes:  0 '***' 0.001 '**' 0.01 '*' 0.05 '.' 0.1 ' ' 1

Correlation of Fixed Effects:
   (Intr) I1    
I1  0.000       
I2  0.000 -0.500
```

```
# Hence, we recommended that the specific contrasts of interest are entered 
# directly.

# It is of interest to see what R does if factor I is only included as a fixed 
# effect and the  order of the random effects is varied. See below.    

aov2a_3ints<-aov(y~I+Error(T*B+I:T+I:B+I:T:B), contrasts=list(I=contr.sum),
                 data=CrossedDataset2_3Ints)
summary(aov2a_3ints)
```

```
Error: T
          Df Sum Sq Mean Sq F value Pr(>F)
Residuals 15  133.6   8.907               

Error: B
          Df Sum Sq Mean Sq F value Pr(>F)
Residuals  4  128.2   32.04               

Error: T:B
          Df Sum Sq Mean Sq F value Pr(>F)
Residuals 60  377.1   6.286               

Error: T:I
          Df Sum Sq Mean Sq F value  Pr(>F)   
I          2   59.4  29.698   5.687 0.00805 **
Residuals 30  156.7   5.222                   
---
Signif. codes:  0 '***' 0.001 '**' 0.01 '*' 0.05 '.' 0.1 ' ' 1

Error: B:I
          Df Sum Sq Mean Sq F value Pr(>F)
Residuals  8    130   16.25               

Error: T:B:I
           Df Sum Sq Mean Sq F value Pr(>F)
Residuals 120  510.7   4.256               

Error: Within
           Df Sum Sq Mean Sq F value Pr(>F)
Residuals 240  996.5   4.152
```

```
aov2b_3ints<-aov(y~I+Error(T+B+I:B+I:T:B+T:B+I:T),contrasts=list(I=contr.sum),
                 data=CrossedDataset2_3Ints)
summary(aov2b_3ints)
```

```
Error: T
          Df Sum Sq Mean Sq F value Pr(>F)
Residuals 15  133.6   8.907               

Error: B
          Df Sum Sq Mean Sq F value Pr(>F)
Residuals  4  128.2   32.04               

Error: B:I
          Df Sum Sq Mean Sq F value Pr(>F)
I          2   59.4   29.70   1.827  0.222
Residuals  8  130.0   16.25               

Error: T:B
          Df Sum Sq Mean Sq F value Pr(>F)
Residuals 60  377.1   6.286               

Error: T:I
          Df Sum Sq Mean Sq F value Pr(>F)
Residuals 30  156.7   5.222               

Error: T:B:I
           Df Sum Sq Mean Sq F value Pr(>F)
Residuals 120  510.7   4.256               

Error: Within
           Df Sum Sq Mean Sq F value Pr(>F)
Residuals 240  996.5   4.152
```

```
# Note: the output here shows that the fixed effect of factor I incorrectly appears
# in the W_I∧T or W_I∧C stratum, depending on whether I:T or I:C appears in the 
# random part of the model statement first, respectively.
```

## **3. Multicentre Randomised Block Factorial Design**

### a) Case One - Two Interventions Only

```
CrossedDataset3 <- readRDS("P:/CTRU/Projects/Methodology/2_Statistical Methodology/2022_Rebecca Walwyn_NIHR Advanced Fellowship/7_Papers/2A_Crossed Therapist Designs/4_Submitted_20250930/3_Uploaded to GitHub/CrossedDataset3.rds")

# ANOVA

aov3 <- aov(y~I+Error(B+I+C+T+I:B+C:B+T:B+I:C+I:T+I:C:B+I:T:B), 
            contrasts=list(I=contr.sum), data=CrossedDataset3)
```

```
Warning in aov(y ~ I + Error(B + I + C + T + I:B + C:B + T:B + I:C + I:T + :
Error() model is singular
```

```
summary(aov3)
```

```
Error: B
          Df Sum Sq Mean Sq F value Pr(>F)
Residuals  4  433.7   108.4               

Error: I
  Df Sum Sq Mean Sq
I  1  192.1   192.1

Error: C
          Df Sum Sq Mean Sq F value Pr(>F)
Residuals  5  641.3   128.3               

Error: T
          Df Sum Sq Mean Sq F value Pr(>F)
Residuals 42  585.4   13.94               

Error: B:I
          Df Sum Sq Mean Sq F value Pr(>F)
Residuals  4  48.81    12.2               

Error: B:C
          Df Sum Sq Mean Sq F value Pr(>F)
Residuals 20  272.5   13.63               

Error: B:T
           Df Sum Sq Mean Sq F value Pr(>F)
Residuals 168  654.8   3.898               

Error: I:C
          Df Sum Sq Mean Sq F value Pr(>F)
Residuals  5  99.92   19.98               

Error: I:T
          Df Sum Sq Mean Sq F value Pr(>F)
Residuals 42  288.9   6.878               

Error: B:I:C
          Df Sum Sq Mean Sq F value Pr(>F)
Residuals 20  139.5   6.976               

Error: B:I:T
           Df Sum Sq Mean Sq F value Pr(>F)
Residuals 168  611.1   3.638               

Error: Within
           Df Sum Sq Mean Sq F value Pr(>F)
Residuals 480   1426   2.971
```

```
coefficients(aov3)
```

```
(Intercept) :
(Intercept) 
    47.4406 

B :
numeric(0)

I :
        I1 
-0.4473807 

C :
numeric(0)

T :
numeric(0)

B:I :
numeric(0)

B:C :
numeric(0)

B:T :
numeric(0)

I:C :
numeric(0)

I:T :
numeric(0)

B:I:C :
numeric(0)

B:I:T :
numeric(0)

Within :
numeric(0)
```

```
print(model.tables(aov3,"means"))
```

```
Tables of means
Grand mean
        
47.4406 

 I 
I
    1     2 
46.99 47.89
```

```
# Note: the order of the terms in the error part of the model is important. If 
# Error(I+T+C+B+I:T+I:C+I:B+T:B+C:B+I:C:B+I:T:B) is used, not all of the terms 
# will appear in the output. The fixed effect of I appears in the W_I stratum, 
# where there are no residual degrees of freedom. It can be seen that estimates of
# ξ_B, ξ_C, ξ_T, ξ_I∧B, ξ_C∧B, ξ_T∧B, ξ_I∧C, ξ_I∧T, ξ_I∧C∧B, ξ_I∧T∧B and ξ_E are  
# 108.4, 128.3, 13.94, 12.2, 13.63, 3.898, 19.98, 6.878, 6.976, 3.638 and 2.971 
# respectively. As such, the estimate of ξ_I is 19.98+12.2-6.976=25.204 so the 
# approximate F is 192.1/25.204=7.6218. Using Equation (2), denominator degrees of 
# freedom for this F test are 5.3166. The p-value can be found using 

pf(7.62180606252, 1, 5.316572016, lower.tail=F)
```

```
[1] 0.03726727
```

```
# Note that estimates of δ_0=47.4406 and δ_1=-0.4473807. Finally, note that 
# -0.4473807 is half the difference between the mean of all outcomes on level 1 of
# factor I (46.99) and the mean of all outcomes on level 2 of factor I (47.89).

# For the random effect of factor T, F = 13.94/7.138 = 1.952927991 on 42 and 
# 39.32690436 degrees of freedom, so the p-value is

pf(1.952927991, 42, 39.32690436, lower.tail=F)
```

```
[1] 0.01831872
```

```
# For the random effect of factor C, F = 128.3/33.436 = 3.837181481 on 5 and 
# 11.46814402 degrees of freedom, so the p-value is

pf(3.837181481, 5, 11.46814402, lower.tail=F)
```

```
[1] 0.02781926
```

```
# For the random effect of factor B, F = 108.4/18.854 = 5.749443089 on 4 and 
# 7.264628053 degrees of freedom, so the p-value is

pf(5.749443089, 4, 7.264628053, lower.tail=F)
```

```
[1] 0.02110186
```

```
# For the random effect of factor I∧C, F = 19.98/10.216 = 1.955755677 on 5 and 
# 28.68605702 degrees of freedom, so the p-value is

pf(1.955755677, 5, 28.68505702, lower.tail=F)
```

```
[1] 0.1158165
```

```
# For the random effect of factor C∧B, F = 13.63/7.236 = 1.883637369 on 20 and 
# 20.11937278 degrees of freedom, so the p-value is

pf(1.883637369, 20, 20.11937278, lower.tail=F)
```

```
[1] 0.08226249
```

```
# For the random effect of factor I∧T, F = 6.878/3.638 = 1.89058823 on 42 and 168 
# degrees of freedom, so the p-value is

pf(1.89059923, 42, 168, lower.tail=F)
```

```
[1] 0.00247185
```

```
# For the random effect of factor I∧B, F = 12.2/6.976 = 1.748853211 on 4 and 20 
# degrees of freedom, so the p-value is

pf(1.748853211, 4, 20, lower.tail=F)
```

```
[1] 0.1789188
```

```
# For the random effect of factor T∧B, F = 3.898/3.638 = 1.072467839 on 168 and 168
# degrees of freedom, so the p-value is

pf(1.072467839, 168, 168, lower.tail=F)
```

```
[1] 0.3253868
```

```
# For the random effect of factor I∧C∧B, F = 6.976/3.638 = 1.917537108 on 20 and 
# 168 degrees of freedom, so the p-value is

pf(1.917537108, 20, 168, lower.tail=F)
```

```
[1] 0.0140142
```

```
# For the random effect of factor I∧T∧B, F = 3.638/2.971 = 1.224503534 on 168 and 
# 480 degrees of freedom, so the p-value is

pf(1.224503534, 168, 480, lower.tail=F)
```

```
[1] 0.05031243
```

```
# Regression

library(lmerTest)
reg3 <- lmer(y~I+(1|T)+(1|B)+(1|T:B)+(1|C)+(1|C:B)+(1|I:T)+(1|I:B)+(1|I:T:B)
             +(1|I:C)+(1|I:C:B), contrasts=list(I=contr.sum), data=CrossedDataset3)
summary(reg3)
```

```
Linear mixed model fit by REML. t-tests use Satterthwaite's method [
lmerModLmerTest]
Formula: y ~ I + (1 | T) + (1 | B) + (1 | T:B) + (1 | C) + (1 | C:B) +  
    (1 | I:T) + (1 | I:B) + (1 | I:T:B) + (1 | I:C) + (1 | I:C:B)
   Data: CrossedDataset3

REML criterion at convergence: 4051.4

Scaled residuals: 
     Min       1Q   Median       3Q      Max 
-2.88274 -0.61254 -0.00457  0.62238  2.63089 

Random effects:
 Groups   Name        Variance Std.Dev.
 I:T:B    (Intercept) 0.33309  0.5771  
 T:B      (Intercept) 0.06498  0.2549  
 I:T      (Intercept) 0.32403  0.5692  
 I:C:B    (Intercept) 0.20866  0.4568  
 T        (Intercept) 0.34002  0.5831  
 C:B      (Intercept) 0.19974  0.4469  
 I:C      (Intercept) 0.12209  0.3494  
 I:B      (Intercept) 0.05443  0.2333  
 C        (Intercept) 0.59252  0.7698  
 B        (Intercept) 0.46661  0.6831  
 Residual             2.97152  1.7238  
Number of obs: 960, groups:  
I:T:B, 480; T:B, 240; I:T, 96; I:C:B, 60; T, 48; C:B, 30; I:C, 12; I:B, 10; C, 6; B, 5

Fixed effects:
            Estimate Std. Error      df t value Pr(>|t|)    
(Intercept)  47.4406     0.4820  7.9757  98.420 1.37e-13 ***
I1           -0.4474     0.1620  5.3171  -2.761   0.0373 *  
---
Signif. codes:  0 '***' 0.001 '**' 0.01 '*' 0.05 '.' 0.1 ' ' 1

Correlation of Fixed Effects:
   (Intr)
I1 0.000
```

```
ls_means(reg3, which = "I", pairwise = TRUE)
```

```
Least Squares Means table:

         Estimate Std. Error  df t value     lower     upper Pr(>|t|)  
I1 - I2 -0.894761   0.324092 5.3 -2.7608 -1.713141 -0.076382  0.03726 *
---
Signif. codes:  0 '***' 0.001 '**' 0.01 '*' 0.05 '.' 0.1 ' ' 1

  Confidence level: 95%
  Degrees of freedom method: Satterthwaite
```

```
ranova(reg3)
```

```
boundary (singular) fit: see help('isSingular')
boundary (singular) fit: see help('isSingular')
```

```
ANOVA-like table for random-effects: Single term deletions

Model:
y ~ I + (1 | T) + (1 | B) + (1 | T:B) + (1 | C) + (1 | C:B) + (1 | I:T) + (1 | I:B) + (1 | I:T:B) + (1 | I:C) + (1 | I:C:B)
            npar  logLik    AIC    LRT Df Pr(>Chisq)   
<none>        13 -2025.7 4077.4                        
(1 | T)       12 -2028.0 4080.0 4.6307  1    0.03140 * 
(1 | B)       12 -2027.5 4079.0 3.6520  1    0.05600 . 
(1 | T:B)     12 -2025.8 4075.6 0.2000  1    0.65471   
(1 | C)       12 -2027.2 4078.5 3.0707  1    0.07971 . 
(1 | C:B)     12 -2026.7 4077.4 2.0083  1    0.15644   
(1 | I:T)     12 -2029.8 4083.5 8.0946  1    0.00444 **
(1 | I:B)     12 -2026.0 4076.0 0.5872  1    0.44349   
(1 | I:T:B)   12 -2027.0 4078.0 2.6274  1    0.10503   
(1 | I:C)     12 -2026.3 4076.5 1.1228  1    0.28932   
(1 | I:C:B)   12 -2028.0 4079.9 4.5163  1    0.03357 * 
---
Signif. codes:  0 '***' 0.001 '**' 0.01 '*' 0.05 '.' 0.1 ' ' 1
```

```
# Note: this analysis should be done with the lmer command here. Satterthwaite 
# degrees of freedom for the fixed effect of I are 5.3 as expected. Note that 
# t=-2.7606 (=sqrt(7.622)) and p=0.03727 as before. It can be seen that estimates 
# of σ_v1^2, σ_v2^2, σ_v3^2, σ_v4^2, σ_v5^2, σ_u1^2, σ_u2^2, σ_u3^2, σ_u4^2, σ_u5^2
# and σ_e^2 are 0.32402, 0.05444, 0.33310, 0.12211, 0.20867, 0.34001, 0.46652, 
# 0.06500, 0.59267, 0.19972 and 2.97150 respectively. Using Equations (E.10) to 
# (E.20), these give estimates of ξ_T, ξ_C, ξ_B, ξ_I∧T, ξ_I∧C, ξ_I∧B, ξ_T∧B, ξ_C∧B,
# ξ_I∧C∧B, ξ_I∧T∧B and ξ_E above precisely. As before, it can be seen that estimates
# of δ_0=47.4406 and δ_1=-0.4474, and that -0.4474 is half the difference between 
# the means of all outcomes on levels 1 and 2 of factor I, given as -0.89476. Note 
# that the standard error of the estimate of δ_0 is 0.1621 which is also half 
# 0.32411.

# It is of interest to see what R does if factor I is only included as a fixed 
# effect and the  order of the random effects is varied. See below.  

aov3a<-aov(y~I+Error(B+C+T+I:B+C:B+T:B+I:C+I:T+I:C:B+I:T:B),
           contrasts=list(I=contr.sum),data= CrossedDataset3)
```

```
Warning in aov(y ~ I + Error(B + C + T + I:B + C:B + T:B + I:C + I:T + I:C:B +
: Error() model is singular
```

```
summary(aov3a)
```

```
Error: B
          Df Sum Sq Mean Sq F value Pr(>F)
Residuals  4  433.7   108.4               

Error: C
          Df Sum Sq Mean Sq F value Pr(>F)
Residuals  5  641.3   128.3               

Error: T
          Df Sum Sq Mean Sq F value Pr(>F)
Residuals 42  585.4   13.94               

Error: B:I
          Df Sum Sq Mean Sq F value Pr(>F)  
I          1 192.14   192.1   15.75 0.0166 *
Residuals  4  48.81    12.2                 
---
Signif. codes:  0 '***' 0.001 '**' 0.01 '*' 0.05 '.' 0.1 ' ' 1

Error: B:C
          Df Sum Sq Mean Sq F value Pr(>F)
Residuals 20  272.5   13.63               

Error: B:T
           Df Sum Sq Mean Sq F value Pr(>F)
Residuals 168  654.8   3.898               

Error: C:I
          Df Sum Sq Mean Sq F value Pr(>F)
Residuals  5  99.92   19.98               

Error: T:I
          Df Sum Sq Mean Sq F value Pr(>F)
Residuals 42  288.9   6.878               

Error: B:C:I
          Df Sum Sq Mean Sq F value Pr(>F)
Residuals 20  139.5   6.976               

Error: B:T:I
           Df Sum Sq Mean Sq F value Pr(>F)
Residuals 168  611.1   3.638               

Error: Within
           Df Sum Sq Mean Sq F value Pr(>F)
Residuals 480   1426   2.971
```

```
aov3b<-aov(y~I+Error(B+C+T+C:B+T:B+I:C+I:T+I:B+I:C:B+I:T:B),
           contrasts=list(I=contr.sum),data=CrossedDataset3)
```

```
Warning in aov(y ~ I + Error(B + C + T + C:B + T:B + I:C + I:T + I:B + I:C:B +
: Error() model is singular
```

```
summary(aov3b)
```

```
Error: B
          Df Sum Sq Mean Sq F value Pr(>F)
Residuals  4  433.7   108.4               

Error: C
          Df Sum Sq Mean Sq F value Pr(>F)
Residuals  5  641.3   128.3               

Error: T
          Df Sum Sq Mean Sq F value Pr(>F)
Residuals 42  585.4   13.94               

Error: B:C
          Df Sum Sq Mean Sq F value Pr(>F)
Residuals 20  272.5   13.63               

Error: B:T
           Df Sum Sq Mean Sq F value Pr(>F)
Residuals 168  654.8   3.898               

Error: C:I
          Df Sum Sq Mean Sq F value Pr(>F)  
I          1 192.14  192.14   9.615 0.0268 *
Residuals  5  99.92   19.98                 
---
Signif. codes:  0 '***' 0.001 '**' 0.01 '*' 0.05 '.' 0.1 ' ' 1

Error: T:I
          Df Sum Sq Mean Sq F value Pr(>F)
Residuals 42  288.9   6.878               

Error: B:I
          Df Sum Sq Mean Sq F value Pr(>F)
Residuals  4  48.81    12.2               

Error: B:C:I
          Df Sum Sq Mean Sq F value Pr(>F)
Residuals 20  139.5   6.976               

Error: B:T:I
           Df Sum Sq Mean Sq F value Pr(>F)
Residuals 168  611.1   3.638               

Error: Within
           Df Sum Sq Mean Sq F value Pr(>F)
Residuals 480   1426   2.971
```

```
aov3c<-aov(y~I+Error(B+C+T+C:B+T:B+I:T+I:C+I:C:B+I:T:B+I:B),
           contrasts=list(I=contr.sum),data=CrossedDataset3)
```

```
Warning in aov(y ~ I + Error(B + C + T + C:B + T:B + I:T + I:C + I:C:B + :
Error() model is singular
```

```
summary(aov3c)
```

```
Error: B
          Df Sum Sq Mean Sq F value Pr(>F)
Residuals  4  433.7   108.4               

Error: C
          Df Sum Sq Mean Sq F value Pr(>F)
Residuals  5  641.3   128.3               

Error: T
          Df Sum Sq Mean Sq F value Pr(>F)
Residuals 42  585.4   13.94               

Error: B:C
          Df Sum Sq Mean Sq F value Pr(>F)
Residuals 20  272.5   13.63               

Error: B:T
           Df Sum Sq Mean Sq F value Pr(>F)
Residuals 168  654.8   3.898               

Error: T:I
          Df Sum Sq Mean Sq F value   Pr(>F)    
I          1  192.1  192.14   23.23 1.54e-05 ***
Residuals 47  388.8    8.27                     
---
Signif. codes:  0 '***' 0.001 '**' 0.01 '*' 0.05 '.' 0.1 ' ' 1

Error: B:I
          Df Sum Sq Mean Sq F value Pr(>F)
Residuals  4  48.81    12.2               

Error: B:C:I
          Df Sum Sq Mean Sq F value Pr(>F)
Residuals 20  139.5   6.976               

Error: B:T:I
           Df Sum Sq Mean Sq F value Pr(>F)
Residuals 168  611.1   3.638               

Error: Within
           Df Sum Sq Mean Sq F value Pr(>F)
Residuals 480   1426   2.971
```

```
# Note that the output here shows that the fixed effect of I incorrectly appears in
# the W_I∧T, W_I∧B or W_I∧C stratum, depending on whether I:T, I:B or I:C appears in
# the random part of the model statement first, respectively. Note also that when 
# I:C appears later in the random part of the model statement, it is not included 
# in the output.
```

### b) Case Two - Three Interventions

```
CrossedDataset3_3Ints <- readRDS("P:/CTRU/Projects/Methodology/2_Statistical Methodology/2022_Rebecca Walwyn_NIHR Advanced Fellowship/7_Papers/2A_Crossed Therapist Designs/4_Submitted_20250930/3_Uploaded to GitHub/CrossedDataset3_3Ints.rds")

# ANOVA

aov3_3ints_1<-aov(y~I+Error(B+I+C+T+I:B+C:B+T:B+I:C+I:T+I:C:B+I:T:B),
                  contrasts=list(I=contr.sum),data=CrossedDataset3_3Ints)
```

```
Warning in aov(y ~ I + Error(B + I + C + T + I:B + C:B + T:B + I:C + I:T + :
Error() model is singular
```

```
summary(aov3_3ints_1)
```

```
Error: B
          Df Sum Sq Mean Sq F value Pr(>F)
Residuals  4  442.1   110.5               

Error: I
  Df Sum Sq Mean Sq
I  2   46.8    23.4

Error: C
          Df Sum Sq Mean Sq F value Pr(>F)
Residuals  5  143.7   28.73               

Error: T
          Df Sum Sq Mean Sq F value Pr(>F)
Residuals 42  253.3   6.032               

Error: B:I
          Df Sum Sq Mean Sq F value Pr(>F)
Residuals  8    467   58.37               

Error: B:C
          Df Sum Sq Mean Sq F value Pr(>F)
Residuals 20  339.6   16.98               

Error: B:T
           Df Sum Sq Mean Sq F value Pr(>F)
Residuals 168   1361   8.102               

Error: I:C
          Df Sum Sq Mean Sq F value Pr(>F)
Residuals 10  140.5   14.05               

Error: I:T
          Df Sum Sq Mean Sq F value Pr(>F)
Residuals 84  596.3   7.099               

Error: B:I:C
          Df Sum Sq Mean Sq F value Pr(>F)
Residuals 40  379.6    9.49               

Error: B:I:T
           Df Sum Sq Mean Sq F value Pr(>F)
Residuals 336   1987   5.915               

Error: Within
           Df Sum Sq Mean Sq F value Pr(>F)
Residuals 720   4129   5.734
```

```
print(model.tables(aov3_3ints_1,"means"))
```

```
Tables of means
Grand mean
         
49.37575 

 I 
I
    1     2     3 
49.21 49.63 49.29
```

```
# Note: the fixed effect of I appears in the W_I stratum, where there are no 
# residual degrees of freedom. It can be seen that estimates of ξ_B, ξ_C, ξ_T, 
# ξ_I∧B, ξ_C∧B, ξ_T∧B, ξ_I∧C, ξ_I∧T, ξ_I∧C∧B, ξ_I∧T∧B and ξ_E are 100.5, 4.13, 3.15,
# 56.53, 14.54, 3.585, 16.51, 3.281, 3.119, 2.777 and 2.605 respectively. As such, 
# the estimate of ξ_I is 16.51+56.53-3.119=69.921 so the approximate F is 
# 51.75/69.921=0.7401. Using Equation (2), denominator degrees of freedom for this F
# test are 11.4507. The p-value can be found using 

pf(0.740120994, 2, 11.45069399, lower.tail=F)
```

```
[1] 0.4985541
```

```
# For the random effect of factor T, F = 3.15/4.089 = 0.770359501 on 42 and 
# 73.45953981 degrees of freedom, so the p-value is

pf(0.770359501, 42, 73.45953981, lower.tail=F)
```

```
[1] 0.8191667
```

```
# For the random effect of factor C, F = 4.13/26.992 = 0.153008299 on 5 and 
# 18.90633843 degrees of freedom, so the p-value is

pf(0.153008299, 5, 18.90633843, lower.tail=F)
```

```
[1] 0.9765481
```

```
# For the random effect of factor B, F = 100.5/67.951 = 1.479006931 on 4 and 
# 11.25441982 degrees of freedom, so the p-value is

pf(1.479006931, 4, 11.25441982, lower.tail=F)
```

```
[1] 0.2727823
```

```
# For the random effect of factor I∧C, F = 16.51/3.623 = 4.556996964 on 10 and 
# 33.28886518 degrees of freedom, so the p-value is

pf(4.556996964, 10, 33.28886518, lower.tail=F)
```

```
[1] 0.0004289431
```

```
# For the random effect of factor C∧B, F = 14.54/3.927 = 3.702571938 on 20 and 
# 45.00515606 degrees of freedom, so the p-value is

pf(3.702571938, 20, 45.00515606, lower.tail=F)
```

```
[1] 0.000133035
```

```
# For the random effect of factor I∧T, F = 3.281/2.777 = 1.181490817 on 84 and 336 
# degrees of freedom, so the p-value is

pf(1.181490817, 84, 336, lower.tail=F)
```

```
[1] 0.154859
```

```
# For the random effect of factor I∧B, F = 56.53/3.119 = 18.12439885 on 8 and 40 
# degrees of freedom, so the p-value is

pf(18.12439885, 8, 40, lower.tail=F)
```

```
[1] 4.42582e-11
```

```
# For the random effect of factor T∧B, F = 3.585/2.777 = 1.290961469 on 168 and 336
# degrees of freedom, so the p-value is

pf(1.290961469, 168, 336, lower.tail=F)
```

```
[1] 0.0256452
```

```
# For the random effect of factor I∧C∧B, F = 3.119/2.777 = 1.123154483 on 40 and 
# 336 degrees of freedom, so the p-value is

pf(1.123154483, 40, 336, lower.tail=F)
```

```
[1] 0.2878885
```

```
# For the random effect of factor I∧T∧B, F = 2.777/2.605 = 1.066026871 on 336 and 
# 720 degrees of freedom, so the p-value is

pf(1.066026871, 336, 720, lower.tail=F)
```

```
[1] 0.2426198
```

```
# Note that no specific contrasts are given in the output. The coefficients command
# does not give the orthogonal contrasts of interest so are not provided here. 

# It may be desirable to fit specific contrasts of interest – first contrast1 and contrast2

aov3_3ints_2<-aov(y~contrast1+contrast2+Error(B+I+C+T+I:B+C:B+T:B+I:C+I:T+I:C:B
                                              +I:T:B),data=CrossedDataset3_3Ints)
```

```
Warning in aov(y ~ contrast1 + contrast2 + Error(B + I + C + T + I:B + C:B + :
Error() model is singular
```

```
summary(aov3_3ints_2)
```

```
Error: B
          Df Sum Sq Mean Sq F value Pr(>F)
Residuals  4  442.1   110.5               

Error: I
          Df Sum Sq Mean Sq
contrast1  1   5.64    5.64
contrast2  1  41.16   41.16

Error: C
          Df Sum Sq Mean Sq F value Pr(>F)
Residuals  5  143.7   28.73               

Error: T
          Df Sum Sq Mean Sq F value Pr(>F)
Residuals 42  253.3   6.032               

Error: B:I
          Df Sum Sq Mean Sq F value Pr(>F)
Residuals  8    467   58.37               

Error: B:C
          Df Sum Sq Mean Sq F value Pr(>F)
Residuals 20  339.6   16.98               

Error: B:T
           Df Sum Sq Mean Sq F value Pr(>F)
Residuals 168   1361   8.102               

Error: I:C
          Df Sum Sq Mean Sq F value Pr(>F)
Residuals 10  140.5   14.05               

Error: I:T
          Df Sum Sq Mean Sq F value Pr(>F)
Residuals 84  596.3   7.099               

Error: B:I:C
          Df Sum Sq Mean Sq F value Pr(>F)
Residuals 40  379.6    9.49               

Error: B:I:T
           Df Sum Sq Mean Sq F value Pr(>F)
Residuals 336   1987   5.915               

Error: Within
           Df Sum Sq Mean Sq F value Pr(>F)
Residuals 720   4129   5.734
```

```
# Then contrast2 and contrast3

aov3_3ints_3<-aov(y~contrast2+contrast3+Error(B+I+C+T+I:B+C:B+T:B+I:C+I:T+I:C:B
                                              +I:T:B),data=CrossedDataset3_3Ints)
```

```
Warning in aov(y ~ contrast2 + contrast3 + Error(B + I + C + T + I:B + C:B + :
Error() model is singular
```

```
summary(aov3_3ints_3)
```

```
Error: B
          Df Sum Sq Mean Sq F value Pr(>F)
Residuals  4  442.1   110.5               

Error: I
          Df Sum Sq Mean Sq
contrast2  1  41.16   41.16
contrast3  1   5.64    5.64

Error: C
          Df Sum Sq Mean Sq F value Pr(>F)
Residuals  5  143.7   28.73               

Error: T
          Df Sum Sq Mean Sq F value Pr(>F)
Residuals 42  253.3   6.032               

Error: B:I
          Df Sum Sq Mean Sq F value Pr(>F)
Residuals  8    467   58.37               

Error: B:C
          Df Sum Sq Mean Sq F value Pr(>F)
Residuals 20  339.6   16.98               

Error: B:T
           Df Sum Sq Mean Sq F value Pr(>F)
Residuals 168   1361   8.102               

Error: I:C
          Df Sum Sq Mean Sq F value Pr(>F)
Residuals 10  140.5   14.05               

Error: I:T
          Df Sum Sq Mean Sq F value Pr(>F)
Residuals 84  596.3   7.099               

Error: B:I:C
          Df Sum Sq Mean Sq F value Pr(>F)
Residuals 40  379.6    9.49               

Error: B:I:T
           Df Sum Sq Mean Sq F value Pr(>F)
Residuals 336   1987   5.915               

Error: Within
           Df Sum Sq Mean Sq F value Pr(>F)
Residuals 720   4129   5.734
```

```
# Note whether contrast1 or contrast3 is included makes no difference in the ANOVA 
# model. The sums of squares for the two orthogonal contrasts (i.e., 65.66 and 
# 37.85) add up to the sum of squares for the overall effect of factor I (i.e., 
# 103.51).

# For the fixed effect of contrast1, F = 65.66/69.921 = 0.93905979605 on 1 and 
# 11.45069399 degrees of freedom, so the p-value is

pf(0.93905979605, 1, 11.45069399, lower.tail=F)
```

```
[1] 0.3525433
```

```
# For the fixed effect of contrast2, F = 37.85/69.921 = 0.54132520987 on 1 and 
# 11.45069399 degrees of freedom, so the p-value is

pf(0.54132520987, 1, 11.45069399, lower.tail=F)
```

```
[1] 0.4766853
```

```
#Regression – first contrast1 and contrast2

library(lmerTest)
reg3_3ints_1<-lmer(y~contrast1+contrast2+(1|T)+(1|B)+(1|T:B)+(1|C)+(1|C:B)
                   +(1|I:T)+(1|I:B)+(1|I:T:B)+(1|I:C)+(1|I:C:B),
                   data=CrossedDataset3_3Ints)
```

```
boundary (singular) fit: see help('isSingular')
```

```
summary(reg3_3ints_1)
```

```
Linear mixed model fit by REML. t-tests use Satterthwaite's method [
lmerModLmerTest]
Formula: y ~ contrast1 + contrast2 + (1 | T) + (1 | B) + (1 | T:B) + (1 |  
    C) + (1 | C:B) + (1 | I:T) + (1 | I:B) + (1 | I:T:B) + (1 |  
    I:C) + (1 | I:C:B)
   Data: CrossedDataset3_3Ints

REML criterion at convergence: 6790

Scaled residuals: 
     Min       1Q   Median       3Q      Max 
-2.85652 -0.62898 -0.07832  0.69345  2.40944 

Random effects:
 Groups   Name        Variance Std.Dev.
 I:T:B    (Intercept) 0.16129  0.4016  
 T:B      (Intercept) 0.26512  0.5149  
 I:T      (Intercept) 0.03952  0.1988  
 I:C:B    (Intercept) 0.21455  0.4632  
 T        (Intercept) 0.00000  0.0000  
 C:B      (Intercept) 0.12291  0.3506  
 I:C      (Intercept) 0.05204  0.2281  
 I:B      (Intercept) 0.50913  0.7135  
 C        (Intercept) 0.02997  0.1731  
 B        (Intercept) 0.15508  0.3938  
 Residual             5.73428  2.3946  
Number of obs: 1440, groups:  
I:T:B, 720; T:B, 240; I:T, 144; I:C:B, 90; T, 48; C:B, 30; I:C, 18; I:B, 15; C, 6; B, 5

Fixed effects:
            Estimate Std. Error       df t value Pr(>|t|)    
(Intercept) 49.37575    0.29139  4.62450 169.447 5.96e-10 ***
contrast1    0.04426    0.14781  8.84226   0.299    0.772    
contrast2   -0.20707    0.25602  8.84226  -0.809    0.440    
---
Signif. codes:  0 '***' 0.001 '**' 0.01 '*' 0.05 '.' 0.1 ' ' 1

Correlation of Fixed Effects:
          (Intr) cntrs1
contrast1 0.000        
contrast2 0.000  0.000 
optimizer (nloptwrap) convergence code: 0 (OK)
boundary (singular) fit: see help('isSingular')
```

```
ranova(reg3_3ints_1)
```

```
boundary (singular) fit: see help('isSingular')
boundary (singular) fit: see help('isSingular')
boundary (singular) fit: see help('isSingular')
boundary (singular) fit: see help('isSingular')
boundary (singular) fit: see help('isSingular')
boundary (singular) fit: see help('isSingular')
boundary (singular) fit: see help('isSingular')
boundary (singular) fit: see help('isSingular')
boundary (singular) fit: see help('isSingular')
```

```
ANOVA-like table for random-effects: Single term deletions

Model:
y ~ contrast1 + contrast2 + (1 | T) + (1 | B) + (1 | T:B) + (1 | C) + (1 | C:B) + (1 | I:T) + (1 | I:B) + (1 | I:T:B) + (1 | I:C) + (1 | I:C:B)
            npar  logLik    AIC     LRT Df Pr(>Chisq)    
<none>        14 -3395.0 6818.0                          
(1 | T)       13 -3395.0 6816.0  0.0000  1    1.00000    
(1 | B)       13 -3395.2 6816.4  0.4039  1    0.52508    
(1 | T:B)     13 -3396.8 6819.7  3.7281  1    0.05351 .  
(1 | C)       13 -3395.1 6816.1  0.1570  1    0.69197    
(1 | C:B)     13 -3395.7 6817.3  1.3516  1    0.24501    
(1 | I:T)     13 -3395.1 6816.1  0.1792  1    0.67205    
(1 | I:B)     13 -3402.7 6831.5 15.5184  1  8.171e-05 ***
(1 | I:T:B)   13 -3395.1 6816.3  0.3413  1    0.55908    
(1 | I:C)     13 -3395.2 6816.5  0.5191  1    0.47123    
(1 | I:C:B)   13 -3397.0 6820.0  4.0432  1    0.04435 *  
---
Signif. codes:  0 '***' 0.001 '**' 0.01 '*' 0.05 '.' 0.1 ' ' 1
```

```
# Note: this analysis should be done with the lmer command used here. Satterthwaite
# degrees of freedom for the fixed effect of I are 10.7830 rather than 11.4507 as 
# expected. For contrast1 t=-0.992 ≠ sqrt(0.93905979605) and p=0.343 rather than 
# 0.3525433. For contrast2 t=0.753 ≠ sqrt(0.54132520987) and p=0.753 rather than 
# 0.4766853.

# We then fit the regression model using contrast2 and contrast3

reg3_3ints_2<-lmer(y~contrast2+contrast3+(1|T)+(1|B)+(1|T:B)+(1|C)+(1|C:B)
                   +(1|I:T)+(1|I:B)+(1|I:T:B)+(1|I:C)+(1|I:C:B),
                   data=CrossedDataset3_3Ints)
```

```
boundary (singular) fit: see help('isSingular')
```

```
summary(reg3_3ints_2)
```

```
Linear mixed model fit by REML. t-tests use Satterthwaite's method [
lmerModLmerTest]
Formula: y ~ contrast2 + contrast3 + (1 | T) + (1 | B) + (1 | T:B) + (1 |  
    C) + (1 | C:B) + (1 | I:T) + (1 | I:B) + (1 | I:T:B) + (1 |  
    I:C) + (1 | I:C:B)
   Data: CrossedDataset3_3Ints

REML criterion at convergence: 6788.6

Scaled residuals: 
     Min       1Q   Median       3Q      Max 
-2.85653 -0.62899 -0.07832  0.69344  2.40944 

Random effects:
 Groups   Name        Variance Std.Dev.
 I:T:B    (Intercept) 0.16126  0.4016  
 T:B      (Intercept) 0.26512  0.5149  
 I:T      (Intercept) 0.03951  0.1988  
 I:C:B    (Intercept) 0.21454  0.4632  
 T        (Intercept) 0.00000  0.0000  
 C:B      (Intercept) 0.12293  0.3506  
 I:C      (Intercept) 0.05204  0.2281  
 I:B      (Intercept) 0.50917  0.7136  
 C        (Intercept) 0.02997  0.1731  
 B        (Intercept) 0.15510  0.3938  
 Residual             5.73431  2.3946  
Number of obs: 1440, groups:  
I:T:B, 720; T:B, 240; I:T, 144; I:C:B, 90; T, 48; C:B, 30; I:C, 18; I:B, 15; C, 6; B, 5

Fixed effects:
            Estimate Std. Error       df t value Pr(>|t|)    
(Intercept) 49.37575    0.29141  4.62412 169.439 5.97e-10 ***
contrast2   -0.20707    0.25603  8.84180  -0.809    0.440    
contrast3    0.08851    0.29563  8.84180   0.299    0.772    
---
Signif. codes:  0 '***' 0.001 '**' 0.01 '*' 0.05 '.' 0.1 ' ' 1

Correlation of Fixed Effects:
          (Intr) cntrs2
contrast2 0.000        
contrast3 0.000  0.000 
optimizer (nloptwrap) convergence code: 0 (OK)
boundary (singular) fit: see help('isSingular')
```

```
# Note: the estimates of σ_v1^2, σ_v2^2, σ_v3^2, σ_v4^2, σ_v5^2, σ_u1^2, σ_u2^2,
# σ_u3^2, σ_u4^2, σ_u5^2 and σ_e^2 are unstable, changing depending on whether 
# contrast1 or contrast3 is included in the model. They also do not give ξ_T, ξ_C,
# ξ_B, ξ_I∧T, ξ_I∧C, ξ_I∧B, ξ_T∧B, ξ_C∧B, ξ_I∧C∧B, ξ_I∧T∧B and ξ_E above using 
# Equations (E.10) to (E.20). The reason for this is the presence of some variance 
# components estimated to be zero. Under these circumstances, we would argue that 
# ANOVA is preferable to regression.

# It is of interest to see what R does if factor I is only included as a fixed 
# effect and the order of the random effects is varied. See below.    

aov3a_3ints<-aov(y~I+Error(B+C+T+I:B+C:B+T:B+I:C+I:T+I:C:B+I:T:B),
                 data=CrossedDataset3_3Ints)
```

```
Warning in aov(y ~ I + Error(B + C + T + I:B + C:B + T:B + I:C + I:T + I:C:B +
: Error() model is singular
```

```
summary(aov3a_3ints)
```

```
Error: B
          Df Sum Sq Mean Sq F value Pr(>F)
Residuals  4  442.1   110.5               

Error: C
          Df Sum Sq Mean Sq F value Pr(>F)
Residuals  5  143.7   28.73               

Error: T
          Df Sum Sq Mean Sq F value Pr(>F)
Residuals 42  253.3   6.032               

Error: B:I
          Df Sum Sq Mean Sq F value Pr(>F)
I          2   46.8   23.40   0.401  0.682
Residuals  8  467.0   58.37               

Error: B:C
          Df Sum Sq Mean Sq F value Pr(>F)
Residuals 20  339.6   16.98               

Error: B:T
           Df Sum Sq Mean Sq F value Pr(>F)
Residuals 168   1361   8.102               

Error: C:I
          Df Sum Sq Mean Sq F value Pr(>F)
Residuals 10  140.5   14.05               

Error: T:I
          Df Sum Sq Mean Sq F value Pr(>F)
Residuals 84  596.3   7.099               

Error: B:C:I
          Df Sum Sq Mean Sq F value Pr(>F)
Residuals 40  379.6    9.49               

Error: B:T:I
           Df Sum Sq Mean Sq F value Pr(>F)
Residuals 336   1987   5.915               

Error: Within
           Df Sum Sq Mean Sq F value Pr(>F)
Residuals 720   4129   5.734
```

```
aov3b_3ints<-aov(y~I+Error(B+C+T+C:B+T:B+I:C+I:T+I:B+I:C:B+I:T:B),
                 data=CrossedDataset3_3Ints)
```

```
Warning in aov(y ~ I + Error(B + C + T + C:B + T:B + I:C + I:T + I:B + I:C:B +
: Error() model is singular
```

```
summary(aov3b_3ints)
```

```
Error: B
          Df Sum Sq Mean Sq F value Pr(>F)
Residuals  4  442.1   110.5               

Error: C
          Df Sum Sq Mean Sq F value Pr(>F)
Residuals  5  143.7   28.73               

Error: T
          Df Sum Sq Mean Sq F value Pr(>F)
Residuals 42  253.3   6.032               

Error: B:C
          Df Sum Sq Mean Sq F value Pr(>F)
Residuals 20  339.6   16.98               

Error: B:T
           Df Sum Sq Mean Sq F value Pr(>F)
Residuals 168   1361   8.102               

Error: C:I
          Df Sum Sq Mean Sq F value Pr(>F)
I          2   46.8   23.40   1.666  0.237
Residuals 10  140.5   14.05               

Error: T:I
          Df Sum Sq Mean Sq F value Pr(>F)
Residuals 84  596.3   7.099               

Error: B:I
          Df Sum Sq Mean Sq F value Pr(>F)
Residuals  8    467   58.37               

Error: B:C:I
          Df Sum Sq Mean Sq F value Pr(>F)
Residuals 40  379.6    9.49               

Error: B:T:I
           Df Sum Sq Mean Sq F value Pr(>F)
Residuals 336   1987   5.915               

Error: Within
           Df Sum Sq Mean Sq F value Pr(>F)
Residuals 720   4129   5.734
```

```
aov3c_3ints<-aov(y~I+Error(B+C+T+C:B+T:B+I:T+I:C+I:C:B+I:T:B+I:B),
                 data=CrossedDataset3_3Ints)
```

```
Warning in aov(y ~ I + Error(B + C + T + C:B + T:B + I:T + I:C + I:C:B + :
Error() model is singular
```

```
summary(aov3c_3ints)
```

```
Error: B
          Df Sum Sq Mean Sq F value Pr(>F)
Residuals  4  442.1   110.5               

Error: C
          Df Sum Sq Mean Sq F value Pr(>F)
Residuals  5  143.7   28.73               

Error: T
          Df Sum Sq Mean Sq F value Pr(>F)
Residuals 42  253.3   6.032               

Error: B:C
          Df Sum Sq Mean Sq F value Pr(>F)
Residuals 20  339.6   16.98               

Error: B:T
           Df Sum Sq Mean Sq F value Pr(>F)
Residuals 168   1361   8.102               

Error: T:I
          Df Sum Sq Mean Sq F value Pr(>F)  
I          2   46.8  23.401   2.985 0.0553 .
Residuals 94  736.8   7.839                 
---
Signif. codes:  0 '***' 0.001 '**' 0.01 '*' 0.05 '.' 0.1 ' ' 1

Error: B:I
          Df Sum Sq Mean Sq F value Pr(>F)
Residuals  8    467   58.37               

Error: B:C:I
          Df Sum Sq Mean Sq F value Pr(>F)
Residuals 40  379.6    9.49               

Error: B:T:I
           Df Sum Sq Mean Sq F value Pr(>F)
Residuals 336   1987   5.915               

Error: Within
           Df Sum Sq Mean Sq F value Pr(>F)
Residuals 720   4129   5.734
```

```
# Note that the output here shows that the fixed effect of I incorrectly appears in
# the W_I∧T, W_I∧B or W_I∧C stratum, depending on whether I:T, I:B or I:C appears in
# the random part of the model statement first, respectively. Note also that when 
# I:C appears later in the random part of the model statement, it is not included 
# in the output.
```
